# Re-Evaluating Multi-Kinase Inhibitors Targeting FGFR and VEGFR Pathways for Combined Therapy in Colorectal Cancer: A Preclinical Study

**DOI:** 10.3390/cancers18193074

**Published:** 2026-09-22

**Authors:** Sergei Boichuk, Tatyana Gessel, Pavel Dunaev, Alina Galyautdinova, Emiliya S. Egorova, Pavel B. Kopnin, Natalya Andriutsa, Aigul Galembikova

**Affiliations:** 1 Department of Pathology, Kazan State Medical University, Kazan 420012, Russia; tatyana.gessel@kazangmu.ru (T.G.); pavel.dunaev@kazangmu.ru (P.D.); alina.galyautdinova@kazangmu.ru (A.G.); aigul.galembikova@kazangmu.ru (A.G.); 2 Department of Radiotherapy and Radiology, Russian Medical Academy of Continuous Professional Education, Moscow 125051, Russia; 3 Central Research Laboratory, Kazan State Medical University, Kazan 420012, Russia; emiliya.egorova@kazangmu.ru; 4 Division of Translational Oncology, N.N. Blokhin National Medical Research Center of Oncology, Moscow 115478, Russia; p.kopnin@ronc.ru; 5 Department of Pathological Physiology, Sechenov First Moscow State Medical University, Moscow 119019, Russia; andriutsa_n_s_@staff.sechenov.ru

**Keywords:** receptor tyrosine kinase inhibitors (RTKIs), colorectal cancer (CRC), resistance, ABC transporters, sensitization, apoptosis

## Abstract

**Background:** Overexpression and increased activity of ATP-binding cassette (ABC) transporters, such as P-glycoprotein (ABCB1/P-gp), breast cancer resistance protein (ABCG2/BCRP), and multidrug resistance-associated proteins (ABCC/MRPs), are known to be among the major contributors to multidrug resistance (MDR) in tumors to structurally similar or even different chemotherapeutic agents, thereby decreasing treatment efficacy. Thus, there is an urgent need to develop novel, effective therapies that overcome MDR and increase chemotherapy response rates. **Results:** We showed here that receptor tyrosine kinase inhibitors (RTKIs) designed to inhibit the overactivated FGFR and/or VEGFR signaling pathways exert an “off-target” effect on MDR proteins in colorectal cancer (CRC), thereby sensitizing CRC to certain chemotherapies. In particular, Pemigatinib and Infigratinib ranked among the top eight tested selective and non-selective FGFR/VEGFR inhibitors that effectively resensitized all types of CRCs to the DNA topoisomerase II inhibitor Doxorubicin and, in fewer experimental settings, to the antimetabolite 5-Fluorouracil. This was evidenced by a significant increase in apoptosis in CRCs treated with the aforementioned chemotherapeutic drugs in combination with RTKIs. Of note, despite the CRCs exhibiting signs of FGFR and VEGFR activation, RTKIs alone exhibited only minor pro-apoptotic and anti-proliferative activities. The enhancement of pro-apoptotic activity of Doxorubicin when co-treated with Pemigatinib, Lucitanib, and Regorafenib in HCT116 cells was consistent with a decrease in proliferative activity in CRCs treated with these RTKIs alone. This was also revealed by high synergy scores (SC) between several RTKIs (e.g., Pemigatinib, Lucitanib, and Regorafenib) and Doxorubicin in CRCs. The aforementioned effects of RTKIs were due to the inhibition of MDR-related ABC transporter activity, particularly ABCB1 and ABCG2. Indeed, in HCT116 cells, Lucitanib and, to a lesser extent, Pemigatinib impaired the efflux of the intrinsically fluorescent Doxorubicin and Mitoxantrone. Similarly, the efflux of Calcein AM, an ABCB1 substrate, was significantly impaired in Lucitanib- and Pemigatinib-treated CRCs, thereby revealing ABCB1 as a molecular target for these RTKIs. Consistent with SC data, Infigratinib and Cabozatinib effectively impaired the efflux of Doxorubicin from Colon-26 cells. Similarly, ABCB1 protein was found to be a primary target for Infigratinib in Colon-26 cancer cells, which was evidenced by competitive assay data based on the combined use of selective ABCB1 and ABCG2 inhibitors (e.g., Tariquidar and Ko-143, respectively). Importantly, ABC-inhibitory effects of RTKIs in CRCs were mediated solely by the impaired efflux of chemotherapeutic agents from CRCs and were not due to the decreased expression of ABC transporters or their subcellular distribution. Lastly, in the small-cohort allograft study, we found Infigratinib to be an effective chemosensitizing agent for the Colon-26 murine colon carcinoma in vivo. This was evidenced by the reduced growth of Colon-26 allografts in the experimental group receiving the combined therapy of Infigratinib and Doxorubicin compared with the single-treated groups. Consistent with these findings, immunohistochemical (IHC) staining revealed a substantial increase in apoptotic cells (e.g., cleaved caspase-3-positive) and a decrease in proliferative cells (e.g., Ki-67-positive) in allografts from animals treated with a combination of Infigratinib and Doxorubicin. **Conclusions:** We show here for the first time that some FGFR inhibitors (e.g., Pemigatinib and Infigratinib) can effectively potentiate cytotoxic and anti-proliferative activity against CRCs overexpressing MDR-related ABC transporters, thereby suggesting potential therapeutic avenues for treating CRC that progresses despite standard conventional therapies.

## Simple Summary

Given that overexpression and increased activity of ATP-binding cassette (ABC) transporters contribute to multidrug resistance (MDR) in human malignancies, there is an urgent need to develop novel, effective therapies that overcome MDR and increase chemotherapy response rates. We show here that some FGFR and VEGFR inhibitors can effectively potentiate the cytotoxic and anti-proliferative activity of certain chemotherapeutic agents, including topoisomerase II inhibitor Doxorubicin and antimetabolite 5-Fluorouracil, against colorectal cancer cells (CRCs) via targeting ABC transporters, thereby providing novel therapeutic avenues for treating CRCs that progress despite standard conventional therapies.

## 1. Introduction

Colorectal cancer (CRC) is one of the most commonly diagnosed human malignancies, which accounts for about 10% of all cancer incidence [1] and is the fifth leading cause of death worldwide [2,3]. Surgery and chemotherapy are currently established treatment options for colon cancer patients, depending upon the tumor location, size, and stage of cancer [4]. Chemotherapy may be given at any stage of the disease and is generally administered in an adjuvant setting (i.e., after surgery). Despite the increase in the overall survival (OS) of patients with colon cancer in recent decades, the vast majority of them eventually develop multidrug resistance (MDR), which decreases the efficacy of the chemotherapeutic agents and ultimately leads to chemotherapy failure [5].

The mechanisms of MDR in cancer are complex, including activation of DNA repair and drug-detoxifying enzymes [6,7], alterations in apoptosis signaling pathways [8,9], development of epithelial-to-mesenchymal transition (EMT) [10,11], presence of cancer stem cells (CSCs) [12], and enhanced efflux of anticancer agents by membrane transporters [13,14]. The last one is based on the enhanced expression and activity of ATP-binding cassette (ABC) superfamily proteins, mainly P-glycoprotein (P-gp/*ABCB1*/MDR1), multidrug resistance protein 1 (MRP-1/*ABCC1*), and breast cancer resistance protein (BCRP/*ABCG2*) [13,14]. P-glycoprotein, encoded by the multidrug resistance 1 (MDR1) gene, is the best-known ABC transporter mediating the efflux of chemotherapeutic agents from cancer cells in an ATP-dependent manner [14]. Indeed, a significant correlation was found between increased P-gp expression in cancer cells and their resistance to a broad spectrum of chemotherapeutic agents. In particular, this includes DNA topoisomerase II inhibitors (e.g., Doxorubicin and Etoposide), mitotic-poisoning agents (e.g., Paclitaxel and Vinblastine), and PARP inhibitors (e.g., Olaparib) [15,16,17,18]. As expected, the elevated expression of P-gp, MRP-1, and BCRP in cancer cells is often associated with reduced chemotherapeutic responses and OS across a broad spectrum of human malignancies, including both solid tumors and blood cancers [19,20,21,22].

Therefore, the development of reversal strategies or the discovery of novel reversal agents to overcome MDR-mediated mechanisms in cancer cells is currently the research focus of cancer therapy. Unfortunately, there is still no therapeutic agent approved by the U.S. Food and Drug Administration (FDA) for patients with multidrug-resistant cancers. Despite the potent synthetic inhibitors of ABCB1 and ABCG2 discovered in recent years [23], the lack of selectivity and unfavorable drug–drug interactions have hindered further development of these inhibitors [24,25]. As an alternative, a tremendous amount of effort, including our own, has recently been devoted to repurposing tyrosine kinase inhibitors (TKIs) as ABCB1 and ABCG2 modulators to enhance the sensitivity of multidrug-resistant cancer cells to conventional chemotherapeutic agents [13,26,27]. This includes Nilotinib, a selective BCR-ABL kinase inhibitor [28], Afatinib, a pan-ErbB inhibitor [29], Imatinib, targeting BCR-ABL and KIT tyrosine kinases [30,31], Dasatinib, a BCR-ABL kinase inhibitor [32], Gefitinib, an EGFR inhibitor [33], Lapatinib, a dual HER2/neu and EGFR inhibitor [34], Erlotinib, an EGFR inhibitor [35], Sunitinib, an inhibitor of PDGFR and VEGFR [36], etc. Moreover, several TKIs exhibited potent ABC-transporter inhibitory activity ex vivo. This was shown for Alectinib, an inhibitor of ALK [37]; Ibrutinib, an inhibitor of BTK [38]; Neratinib (Nerlynx, HKI-272), a dual inhibitor of EGFR and HER2 [39]; and Osimertinib (Tagrisso, Tagrix), a third-generation EGFR inhibitor [40].

We previously showed that BGJ-398 (Infigratinib), a well-known selective FGFR inhibitor, targets P-gp and increases chemotherapy-induced mortality in several multidrug-resistant tumor cell lines, including triple-negative breast cancer and gastrointestinal stromal tumor cells [41]. Given that the MDR phenotype is a common signature of a broad spectrum of human malignancies, including CRC, and taking into account activation of the FGFR and VEGFR pathways, which is frequently detected in CRC [42,43,44,45], we expanded our research to examine whether multi-kinase inhibitors targeting the aforementioned signaling pathways can also exhibit their “off-target” effect and increase the sensitivity of CRC to conventional chemotherapies. For this purpose, we used a panel of cancer cell lines, including HCT116 and RKO human colorectal carcinoma cell lines. The mouse adenocarcinoma cell line Colon-26 was used in both in vitro and in vivo studies to demonstrate enhanced antitumor activity of the chemotherapeutic agent Doxorubicin when combined with an RTKI. The ability of RTKIs to sensitize CRCs to Doxorubicin was examined for TKIs selectively targeting the FGFR pathway (e.g., Infigratinib and Pemigatinib), and multi-kinase RTKIs targeting VEGFR, FGFR, and other signaling pathways (e.g., Axitinib, Regorafenib, Sunitinib, Cabozantinib, Alofanib, and Lucitanib).

We found that Pemigatinib, Lucitanib, Regorafenib, and Infigratinib were the most potent RTKIs, effectively sensitizing CRCs to certain chemotherapeutic agents, including the DNA topoisomerase inhibitors Doxorubicin and Mitoxantrone, as well as the antimetabolite 5-Fluorouracil. Indeed, CRCs treated with chemotherapeutic agents in combination with Regorafenib exhibited a dramatic decrease in cellular viability and proliferative activity when compared with CRCs treated with chemotherapeutic agents or RTKI alone. This was also revealed by high values of synergy scores (SC) between RTKIs and Doxorubicin in cancer cell lines used in the present study. Despite the CRCs exhibiting signs of activation of FGFR and/or VEGFR signaling pathways, the chemosensitizing effects of RTKIs to Doxorubicin and Mitoxantrone were also due to their ability to promote retention within cancer cells. Importantly, by using competitive assays with selective ABCB1 and ABCG2 inhibitors, we identified ABCB1 as a primary target for Infigratinib. Molecular docking analysis revealed that the aforementioned “off-target” effect of this FGFR inhibitor was attributable to its effective binding to the ABCB1 transmembrane domain. Of note, RTKI’s ability was not associated with decreased expression and/or relocalization of the three major ABC transporters (e.g., P-gp, MRP-1, and BCRP). Lastly, we showed that Infigratinib, a selective FGFR inhibitor previously found to be the most effective chemosensitizing agent for Colon-26 murine carcinoma cells in vitro, also sensitized CRCs to Doxorubicin in vivo. This was evidenced by the reduced growth of Colon-26 colon cancer allografts when used in combination with Infigratinib and Doxorubicin. Immunohistochemical (IHC) staining revealed a substantial increase in apoptotic cells (e.g., cleaved caspase-3-positive) in allografts from animals receiving the combined therapy of Infigratinib and Doxorubicin compared with the single-treated groups. Conversely, the number of proliferative cells (e.g., Ki-67-positive cells) was significantly reduced after such combined therapy, suggesting a therapeutic rationale for the combined use of FGFR and/or VEGFR inhibitors with certain chemotherapies, including DNA topoisomerase II inhibitors such as Doxorubicin and Mitoxantrone.

## 2. Materials and Methods

### 2.1. Cell Lines and Culture Conditions

HCT 116 and RKO human colon carcinoma cell lines were purchased from the American Type Culture Collection (ATCC, Manassas, VA, USA), whereas the Colon-26 murine colon adenocarcinoma cell line was obtained from CLS Cell Lines Service GmbH (Eppelheim, Germany). The parental SaOS-2 osteosarcoma and HCC1806 triple-negative breast cancer cells were obtained from the American Type Culture Collection (ATCC, Manassas, VA, USA), whereas Doxorubicin-resistant subline SaOS-2 DoxR and Paclitaxel-resistant HCC1806 Tx-R sublines, as well as Imatinib-resistant gastrointestinal stromal tumor subline, GIST T-1R, were generated previously and characterized in our lab ([46,47,48], respectively). RPMI-1640 medium (Paneco, Moscow, Russia) was used for culturing all the aforementioned cancer cell lines. Fetal bovine serum 15% (Gibco; Thermo Fisher Scientific, Inc., Waltham, MA, USA) and 50 U/mL penicillin with 50 μg/mL streptomycin (Paneco, Moscow, Russia) were added to the medium. A temperature of 37 °C and a humidified atmosphere with 5% CO_2_ were maintained in the CO_2_ incubator (Binder GmbH, Tuttlingen, Germany) for culturing the cancer cells.

### 2.2. Chemical Compounds

Most of the chemical compounds were purchased from SelleckChem (Houston, TX, USA). This included inhibitors of receptor tyrosine kinases (e.g., Infigratinib, Pemigatinib, Alofanib, Axitinib, Lucitanib, Regorafenib, Cabozantinib, and Sunitinib), inhibitors of ABC transporters (e.g., Tariquidar, Mk-571, and Ko-143), and the chemotherapeutic drugs Doxorubicin and 5-Fluorouracil. Mitoxantrone dihydrochloride was acquired from Sigma-Aldrich (Merck KGaA, Darmstadt, Germany). Dimethyl sulfoxide (DMSO) was used for dissolving all compounds in accordance with the manufacturer’s recommendations.

### 2.3. Antibodies

The primary antibodies were purchased from Cell Signaling Technology Inc., Danvers, MA, USA (phospho-FGF Receptor (Tyr653/654)—cat. no. 3471S, FGF Receptor 1 (D8E4)—cat. no. 9740S, FGF Receptor 2 (D4L2V)—cat. no. 23328S, phospho-FRS2-alpha (Tyr196)—cat. no. 3864S, VEGF Receptor 1 (E7T9H)—cat. no. 64094T, phospho-VEGF Receptor 2 (Tyr996)—cat. no. 2474T, VEGF Receptor 2 (D5B1)—cat. no. 9698T, cleaved PARP—cat. no. 5625T, cleaved caspase-3—cat. no. 9661T, actin—cat. no. 4970S); Santa Cruz Biotechnology, Dallas, TX, USA (FRS2 (A-5)—cat. no. sc-17841, MDR1/ABCB1—cat. no. sc-55510, BCRP1/ABCG2—cat. no. sc-58222); Sigma-Aldrich, St. Louis, MO, USA (Anti-phospho-VEGFR1 (pTyr1333)—cat. no. SAB4504006); Abcam plc., Cambridge, UK (anti-MRP1—cat. no. ab260038). The secondary antibodies were obtained from Santa Cruz Biotechnology, Dallas, TX, USA (mouse anti-rabbit IgG-HRP—cat. no. sc-2357, m-IgGκ BP-HRP—cat. no. sc-516102). The antibody concentrations were determined according to the manufacturer’s instructions.

### 2.4. MTS-Based Assay of Interactions Between Chemotherapeutic Drugs

CRCs were seeded into 96-well plates (Corning Inc., Corning, NY, USA). After 24 h of incubation, the culture medium was replaced with a new medium containing DMSO (as a control) and chemotherapeutic compounds alone or in combinations for 72 h. To determine cellular viability, the culture medium was supplemented with MTS reagent (Promega, Madison, WI, USA) and phenazine methosulfate (Sigma-Aldrich, St. Louis, MO, USA) at a 20:1 ratio for 1 h. Detection was performed at 492 nm using a MultiScan FC plate reader (Thermo Fisher Scientific, Waltham, MA, USA). Results were normalized to the untreated control.

Half-inhibitory concentration (IC_50_) was calculated for CRCs treated with Doxorubicin, 5-Fluorouracil (concentrations from 100 to 0.5 μM) alone and with 10 μM of RTKIs, as well as monotherapy with all these inhibitors. The concentration that reduced cellular viability by up to 50% was determined using the online Very Simple IC50 Tool Kit (https://ic50.org/, accessed on 21 March 2026).

For the assessment of interaction type between chemotherapeutic drugs, cells were incubated in culture medium with Doxorubicin (concentrations from 1.0 to 0.125 μM for HCT116, from 2.0 to 0.125 μM for Colon-26 and RKO), 5-Fluorouracil (concentrations from 2.0 to 0.125 μM for HCT116 and Colon-26, from 4.0 to 0.125 μM for RKO), and RTKIs (concentrations from 10 to 1.25 μM) alone and in combination in duplicates. After that, the mean cell viability of CRCs was calculated, normalized to the untreated control, and the data were uploaded to the online SynergyFinder+ Tool (https://synergyfinder.org/#!/, accessed on 8 June 2026). The resulting synergy score (SC) value indicated an antagonistic effect (SC < −10), an additive effect (−10 < SC < 10), or a synergistic effect (SC > 10).

### 2.5. Crystal Violet Staining

Colorectal cancer cells cultured according to the study design, without chemotherapeutic drugs (control), with RTKIs, Doxorubicin, or their combination, were fixed and stained using crystal violet solution (25 mg crystal violet, 1% formaldehyde solution, 1% methanol in 50 mL 1X PBS). The cells were kept in this solution for 20 min at room temperature in the dark and then washed out with running cold water for 2 min and dried on filter paper overnight at room temperature. To extract the crystal violet-fixed solution from cells, 1% SDS was added to the cells, and the cells were incubated for 1 hour at room temperature on a shaker. The optical density of the resulting solution was measured at 540 nm using a MultiScan FC plate reader (Thermo Fisher Scientific, Waltham, MA, USA) in triplicate.

### 2.6. Western Blotting

The protein expression levels were assessed by Western blotting. For this examination, apoptotic cells were separated from the culture medium by centrifugation in phosphate-buffered saline (PBS), and the resulting pellet was added to cells scraped from the dish surface. The cells were incubated in radio-immunoprecipitation (RIPA) buffer (25 mM Tris-HCl, pH 7.6, 5 mM EDTA, 150 mM NaCl, 0.1% SDS, 1% NP-40, 1% sodium deoxycholate, and a cocktail of protease and phosphatase inhibitors) for 20 min on ice. After that, the proteins were separated through centrifugation for 30 min at 11,400 rpm at 2 °C. Their amount was calculated using the Bradford assay. Lysates were diluted in additional RIPA buffer until equal protein concentrations (20 µg) were reached in each sample and then separated by electrophoresis on 4–12% Bis-Tris or 3–8% Tris-acetate NuPAGE gels (Invitrogen, Carlsbad, CA, USA). Then, proteins from gels were transferred to a nitrocellulose membrane (Bio-Rad, Hercules, CA, USA), which was further incubated with primary and secondary antibodies for 12 h at 4 °C and 1 h at room temperature, respectively. For visualization, Western Lightning Plus-ECL reagent (PerkinElmer, Waltham, MA, USA) was used. Western blotting images were analyzed using densitometry analysis in NIH ImageJ software, Version 1.49 (Bethesda, MD, USA).

### 2.7. RNA Extraction and Real-Time Quantitative PCR

Total RNA was extracted from colorectal cancer cell lines according to a standard protocol [40]. In brief, the PCR reaction mix for real-time quantitative PCR (qPCR) was composed of the following components: 1 µL of synthesized cDNA, 2× HS-qPCR SYBR Blue (MHC030-400, Biolabmix, Novosibirsk, Russia), and 10 mM each of forward and reverse PCR primers for *MDR1*, *MRP1*, *ABCG2*, or *GAPDH*, a control gene. Real-time quantitative PCR was performed on the CFX96 Real-Time detection system (Bio-Rad, Hercules, CA, USA) according to the manufacturer’s protocol. The levels of each mRNA were normalized to *GAPDH*. Quantitative data were generated from the number of cycles required for fluorescent detection and amplification of target genes (Ct values). The relative level of expression of the target genes was based on the following formula: 2^−ΔΔCt^.

### 2.8. Real-Time Monitoring of Cell Proliferation

To assess the proliferative activity, the CRCs were seeded into gold microelectrode E-plates (Roche Diagnostics GmbH, Mannheim, Germany) and incubated for 72 h in the presence of DMSO (control), one of the top RTKI (e.g., Pemigatinib, Lucitanib, and Regorafenib—10 µM), Doxorubicin (1 µM), or their combinations. The growth kinetic curves were plotted in real time at 1 h intervals using the iCELLigence system (ACEA Biosciences, San Diego, CA, USA). The normalized cell index was calculated from cell index measurements at each detected point in RTCA software, version 1.2 (Roche Diagnostics GmbH, Mannheim, Germany).

### 2.9. Immunofluorescence Staining

HCT116 and Colon-26 cells were cultured on Poly-L-lysine-coated (Sigma-Aldrich, St. Louis, MO, USA) coverslips in culture medium without (control) or with the studied RTKI (Pemigatinib, Lucitanib, Regorafenib, Infigratinib, and Cabozantinib—10 µM) for 24 h. After that, colon cancer cells were fixed with ice-cold 100% methanol for 15 min at −20 °C, washed out using 1X PBS three times for 5 min in the dark, and incubated in a blocking buffer containing 5% normal goat serum and 0.3% Triton X-100 in 1X PBS for 60 min at room temperature. Further coverslips were incubated with primary antibodies MDR1/ABCB1, MRP1, and ABCG2 (cat. no. sc-55510, sc-18835, and sc-58222, respectively, Santa Cruz Biotechnology, Dallas, TX, USA) overnight at 4 °C. Afterward, cells were washed out using 1X PBS three times for 5 min in the dark with the subsequent incubation of Alexa488-conjugated mouse secondary antibodies (Invitrogen, Carlsbad, CA, USA) diluted in antibody dilution buffer (1X PBS, 1% BSA, 0.3% Triton X-100) for 60 min at room temperature in the dark. After washing out in 1X PBS, DAPI was added to the colon cancer cells for 30 s (Sigma-Aldrich, St. Louis, MI, USA). Coverslips were washed once in 1X PBS and placed on the glass slides. A fluorescent microscope (Olympus BX63, Tokyo, Japan) was used for visualization. The images were obtained by the Spot Advanced Imaging System.

### 2.10. Flow Cytometry

The fluorescence intensity of ABC-transporter substrates was examined using a BD FACS Canto II flow cytometer (Becton Dickinson Biosciences, Franklin Lakes, NJ, USA) with BD FACS Diva Software, version 7.0. In each sample, at least 50,000 events were indicated in 3 independent experiments.

The preparation of cells consisted of sequential replacement of culture medium without (control) or with inhibitors (20 µM of ABC transporter inhibitors or RTKi) for 3 h, substrates (40 µM of Doxorubicin or Mitoxantrone) for 1 h, and repeated addition of corresponding inhibitors or not for 3 h, with washing out using FBS-free culture medium after each step. To perform the flow cytometric examination, CRCs were trypsinized and centrifuged in PBS.

For the Calcein AM assay, the fluorescent compound Calcein AM (cat. no. ab141420, Abcam plc., Cambridge, UK) was used. Briefly, the cells were trypsinized, centrifuged, and incubated with Calcein AM (100 nM) for 30 min at 37 °C. After centrifugation, cells were incubated in culture medium with 20 µM of ABC transporter inhibitors or RTKi for 60 min at 37 °C and subjected to FACS analysis.

For cell cycle analysis, floating apoptotic cells were combined with a cell suspension obtained from the culture dish surface after trypsinization and then centrifuged at 1000 rpm for 3 min. After that, cells were fixed in ice-cold 70% ethanol at −20 °C for 24 h. The cell suspension was washed by centrifugation, resuspended in PBS containing 10 μg/mL propidium iodide (Sigma-Aldrich, St. Louis, MO, USA), and incubated in the dark for 10 min at room temperature.

### 2.11. Molecular Docking

Examination of the potential binding sites of FGFR/VEGFR inhibitors and selective ABC transporter inhibitors, including Tariquidar and Ko-143, was performed using AutoDock v4.2.6 (Center for Computational Structural Biology, CCSB, La Jolla, CA, USA, 2012). The structures of Tariquidar, Ko-143, and RTKIs were obtained from PubChem (https://pubchem.ncbi.nlm.nih.gov/, accessed on 1 June 2025). After adding missing hydrogen atoms to the ligands, geometry optimization was performed using the semi-empirical PM3 method (termination conditions: RMD gradient of 0.1 kcal/(A mol) or 1000 maximum cycles). The ABCB1 (PDB ID: 7A6E) and ABCG2 (PDB ID: 6FEQ) structures were downloaded from the Protein Data Bank (http://www.rcsb.org/, accessed on 9 June 2025) and prepared by removing water and adding missing hydrogen atoms in Python Molecular Viewer (PMV), part of MGLTools version 1.5.7 (Center for Computational Structural Biology, CCSB, La Jolla, CA, USA, 2019).Charges (Kollman and Gasteiger) were also computed there for all ligands and proteins. The grid box included a transmembrane domain of ABCB1 (number of points in dimension x—126, y—126, z—126; spacing—0.7; center x—174.25, y—158.316, z—157.917) or ABCG2 (number of points in dimension x—126, y—126, z—100; spacing—0.7; center x—126.834, y—126.834, z—136.465). The Lamarckian genetic algorithm (number of runs: 50; maximum number of evals: long) was used as a search method. Maximum RMS tolerance for conformational cluster analysis = 2.00 Å. The most favorable position of ligands in the TMD of ABC transporters was selected as the most populated cluster with the lowest binding energy. Docking results were visualized using UCSF Chimera v1.17.3 (University of California, San Francisco, CA, USA, 2023). 2D diagrams were created in BIOVIA Discovery Studio Visualizer (BIOVIA, Dassault Systèmes, San Diego, CA, USA, 2024).

### 2.12. Allograft Studies

Antitumor activity of the studied drugs was investigated at tumor allografts generated by subcutaneous inoculation of 2 × 10^5^/mL Colon-26 cancer cells into the lateral surface of the back of BALB/c mice. When the volume of the tumor reached 50 mm^3^, mice were randomized into 4 groups, which were intraperitoneally injected with 100 µL of solvent (control), Infigratinib (10 mg/kg), Doxorubicin (10 mg/kg), and a combination of both drugs at the mentioned doses 3 times per week for 3 weeks. During the experiment, the general health of the mice was monitored. On the 28th day of the experiment, allografts were isolated, and the final volume (length × width × width × 0.5) and weight were measured. Formalin-fixed, paraffin-embedded (FFPE) tissues were sectioned at 4 μm for hematoxylin and eosin (H&E) staining and subjected to IHC staining for cleaved caspase-3 and Ki-67 expression. Images were captured using ScanScope XT (Aperio Technologies Inc., Vista, CA, USA).

### 2.13. Statistics

All experiments were carried out at least 3 times. Based on these data, results were presented in the article as values ± standard error (SE). Statistical significance for flow cytometry results was determined using one-way ANOVA with Dunnett’s post hoc test. Student’s *t*-test was performed using an online calculator (https://medstatistic.ru/calculators/averagestudent.html, accessed on 24 February 2026).

## 3. Results

### 3.1. Colorectal Cancer Cells (CRCs) Are Characterized by Activation of FGFR and VEGFR Signaling Pathways

To identify FGFR and/or VEGFR inhibitors that synergize with certain chemotherapeutic agents used for CRC therapy, a preselected panel of eight small-molecule inhibitors was screened across three CRC lines, including HCT116, RKO, and Colon-26. Based on the 2D structures, the receptor tyrosine kinase inhibitors (RTKi) were divided into three groups as shown in Figure 1. The first one, which includes Infigratinib and Pemigatinib, contains a 3,5-dimethoxyphenyl and urea in the core (Figure 1A). The second group comprises Lucitanib and Cabozantinib and shares a quinoline ring bearing two methoxy groups at the 6th and 7th positions (Figure 1B). The last one consists of four structurally different TKIs—Alofanib, Axitinib, Sunitinib, and Regorafenib (Figure 1C).

All the aforementioned RTKIs, which effectively target the FGFR and/or VEGFR signaling pathways, alone or in combination with other hyperactivated kinases, were approved for the treatment of a broad spectrum of human malignancies, as shown in Supplementary Table S1 [49,50,51,52,53,54,55,56,57,58,59,60,61,62,63,64,65,66,67,68,69,70].

To examine the abilities of the aforementioned small-molecule inhibitors to modulate Doxorubicin- or 5-Fluorouracil-induced cytotoxicity in CRCs, we first assessed whether FGFR and VEGFR signaling pathways are activated in CRC lines. The GIST T-1R cell line, which acquired secondary resistance to the targeted drug imatinib mesylate due to overactivation of FGFR and VEGFR signaling pathways [48,71], was used as the positive control. The data shown in Figure 2 and File S1 illustrate activation of both signaling pathways in all three CRC lines, thereby providing a molecular basis for the anticancer activities of the aforementioned RTKI against CRCs.

### 3.2. Receptor Tyrosine Kinase Inhibitors Sensitize CRCs to Doxorubicin

To examine the impact of FGFR and VEGFR inhibitors on Doxorubicin- or 5-Fluorouracil-induced cytotoxicity in CRCs, we first assessed the cytotoxic effects of these chemotherapeutic agents. IC_50_ values for the aforementioned chemotherapeutic agents, measured by MTS colorimetric assay after 72 h of exposure, are shown in Table 1 and Supplementary Figure S1.

In particular, we observed a significant decrease in the IC_50_ of Doxorubicin-treated Colon-26 cells when Infigratinib, Pemigatinib, and Cabozantinib were added to the cell cultures. Similarly, Infigratinib potently potentiated the cytotoxic activity of Doxorubicin in HCT116 colon cancer cells. In addition, we also found Axitinib, Lucitanib, Sunitinib, and Regorafenib to be potent chemosensitizing agents in HCT116 cells. In contrast, no such effect was observed in the RKO cell line, probably due to its high sensitivity to Doxorubicin used alone and extremely low IC_50_ values. In contrast, the cytotoxic effect of 5-Fluorouracil was much less potentiated by RTKIs in CRCs (except the RKO cell line), which allowed us to focus our subsequent studies predominantly on the DNA topoisomerase inhibitors Doxorubicin and Mitoxantrone, exhibiting the well-known intrinsic fluorescence activities and therefore making them suitable to examine their intracellular concentrations and ABC-mediated efflux from cancer cells.

Next, we assessed whether RTKIs used at subtoxic concentrations (<IC50) could potentiate the cytotoxicity of the DNA topoisomerase II inhibitor Doxorubicin. The data shown in Figure 3 illustrate that Pemigatinib, Lucitanib, and Regorafenib effectively potentiated the cytotoxic activity of Doxorubicin in HCT116 cells, as evidenced by a significant decrease in confluency and an increase in the number of floating cells.

Similar results were obtained in the Colon-26 cell line, highlighting the high potency of RTKIs in sensitizing it to Doxorubicin (Supplementary Figure S2). Again, Pemigatinib effectively potentiated the cytotoxic activity of Doxorubicin. Additionally, Infigratinib and Cabozantinib enhanced the cytotoxic effect of this topoisomerase II inhibitor, consistent with the IC_50_ values shown in Table 1.

This was consistent with crystal violet staining of CRC cultures treated with these drugs alone or in combination. We observed a moderate decrease in CRC viability in monotherapy, whereas the combinations exerted significant anti-proliferative and cytotoxic effects. Cell viability across three CRC lines treated with Doxorubicin, 5-Fluorouracil, and RTKI, alone or in combination, was assessed using crystal violet staining and is shown in Table 2.

Representative images of crystal violet staining of HCT116 and Colon-26 are shown in Figure 4 and Supplementary Figure S3. For example, the viability of HCT116 cells treated with Doxorubicin in combination with Regorafenib was nearly 0%, highlighting the high potency of this combination (Figure 4A). Quantitative analysis also revealed a substantial decrease in the viability of HCT116 cells treated with a combination of Doxorubicin and Regorafenib, whereas these drugs alone had no effect on cellular viability (Figure 4B). Similarly, Pemigatinib and Lucitanib effectively potentiated the anti-proliferative and cytotoxic activities of Doxorubicin (Figure 4A). These data were also quantified, showing a >70% reduction in cellular viability in HCT116 cells treated with a combination of Doxorubicin and Lucitanib (Figure 4B). Pemigatinib was less potent but induced a statistically significant decrease in HCT116 viability when co-treated with Doxorubicin (Figure 4A,B).

Importantly, a similar pattern was observed in Colon-26 cells treated with the aforementioned combinations of the chemotherapeutic agents (Supplementary Figure S3), thereby highlighting the high potency of these RTKIs in overcoming CRC resistance to conventional chemotherapies. Again, the RTKI pattern that sensitized Colon-26 cells to Doxorubicin differed from that in HCT116 cells and demonstrated high potency of Infigratinib, Pemigatinib, and Cabozantinib in decreasing cellular viability when used in combination with Doxorubicin. This data was in line with the IC_50_ values shown in Table 1. Of note, HCT116 and Colon-26 were much less sensitive to 5-Fluorouracil when used in combination with the aforementioned RTKIs than Doxorubicin-treated cells. In contrast, RKO cells exhibited a prominent cytotoxic effect of 5-Fluorouracil used in combination with some RTKIs. Pemigatinib and Lucitanib were the most effective when used in combination with 5-Fluorouracil.

### 3.3. Synergistic Effect of Receptor Tyrosine Kinase Inhibitors and Doxorubicin in CRCs

The values of the synergistic interactions between the aforementioned RTKIs and Doxorubicin were assessed using the IC_50_-based colorimetric assay and the Highest Single Agent (HSA) model and calculated using the R-package computational tool in the SynergyFinder 2.0 platform. Single-agent dose–response curves showed a concentration-dependent decrease in cellular viability with RTKIs and Doxorubicin alone in HCT116 and Colon-26 cell lines (Figure 5A,D). Strikingly, the dose–response matrices revealed that combined treatment led to markedly greater cytotoxicity, particularly when both agents were used at higher concentrations (Figure 5B,F). Two-dimensional synergy maps illustrated robust (>10) synergy scores (SC) between Pemigatinib and Doxorubicin, with HSA scores of 10.03 for HCT116 cells and 17.16 for Infigratinib and Doxorubicin for Colon-26, thereby illustrating a potent combinatorial effect (Figure 5C,G). This was also supported by three-dimensional HSA synergy landscapes, as shown in Figure 5D,H, which reveal a pronounced combinatorial benefit of the aforementioned RTKIs and Doxorubicin.

Synergy scores (SC), representing synergistic, additive, or antagonistic effects, for all types of RTKIs and chemotherapeutic agents (e.g., Doxorubicin and 5-Fluorouracil), were calculated to identify the most potent combinations for CRC lines and are shown in Table 3.

Consistent with these data, the synergistic or additive effects between the aforementioned RTKIs and Doxorubicin were also assessed using the ZIP model, indicating potent synergy and/or additive effects across all CRC lines treated with Doxorubicin in the presence of RTKIs (Supplementary Table S2). Again, no synergy between RTKIs and 5-Fluorouracil was observed in the RKO cell line, indicating resistance to sensitization. This raises the question of the cause of the differential behavior of RKO and whether the phenotype is more reflective of intrinsic drug susceptibility than of transporter regulation. In particular, this might be due to enhanced DNA repair capacities in this particular cell lime, thereby providing effective repair of DNA lesions induced by chemotherapies. An increased number of cancer stem cells among RKO cells might be an alternative mechanism of their chemoresistance, thereby making RTKI-induced sensitization difficult. The examination of these possibilities was outside the scope of our manuscript and was a subject for further evaluation.

### 3.4. Receptor Tyrosine Kinase Inhibitors Enhance Pro-Apoptotic and Anti-Proliferative Activity of Doxorubicin in CRCs

To further corroborate these findings, we examined whether the RTKIs indicated above potentiate the pro-apoptotic activities of Doxorubicin in CRCs. For this, HCT116 cells were treated with RTKIs and Doxorubicin alone or in combinations for 72 h and subjected to Western blotting to examine the expression of the well-known markers of apoptosis—the cleaved forms of PARP or caspase-3. As expected, no evidence of apoptosis was observed in CRCs treated with the RTKi alone (except for the Lucitanib-treated cells), whereas Doxorubicin treatment resulted in an increase in cleaved PARP in HCT116 cells (Figure 6A, File S1), suggesting the sensitivity of this cancer cell line to Doxorubicin. In contrast to the single-treated HCT116 cells, a significant increase in the expression of the cleaved form of PARP was observed in HCT116 cells treated with a combination of Pemigatinib and Doxorubicin. A similar effect was observed in Doxorubicin-treated HCT116 cells cultured in the presence of Regorafenib. Consistent with these findings, all three types of RTKIs effectively potentiated the cytotoxic effect of Doxorubicin in Colon-26 cells, as evidenced by a significant increase in the expression of the cleaved form of caspase-3 (Figure 6B, File S1). Importantly, no increase in cleaved caspase-3 was detected in single-treated Colon-26 cells, suggesting intrinsic resistance to RTKI and Doxorubicin in this cancer cell line.

Consistent with the enhancement of Doxorubicin-induced pro-apoptotic activity in CRC lines, RTKI potentiated its anti-proliferative activity, as evidenced by a significant reduction in CRC growth kinetics. Of note, Doxorubicin exhibited a minor inhibitory effect on the growth kinetics in HCT116 cells when used alone (Figure 7A–C). Again, the proliferative activity of HCT116 cells decreased significantly when treated with Doxorubicin in the presence of Pemigatinib (Figure 7A). A similar pattern was found in Doxorubicin-treated HCT116 cells cultured in the presence of Lucitanib (Figure 7B) and Regorafenib (Figure 7C). This was expanded to the other chemotherapeutic agents and CRC lines, as well. For example, Colon-26 murine colon adenocarcinoma cells treated with 5-Fluorouracil in combination with various RTKIs (e.g., Infigratinib) also demonstrated reduced proliferative activity compared with single-treated and mock-treated cells (Supplementary Figure S4).

### 3.5. Anti-Proliferative and Pro-Apoptotic Activities of Receptor Tyrosine Kinase Inhibitors Used in Combination with Doxorubicin Are Not Due to the Inhibition of FGFR or VEGFR Signaling Cascades

Although RTKIs alone did not inhibit survival and proliferative capacities in CRC lines, we examined whether their combined activity with Doxorubicin was due to impairment of FGFR or VEGFR signaling pathways. To assess this possibility directly, we examined the levels of the total and phosphorylated forms of FGFR1-2, its adaptor protein FRS-2, and VEGFR1-2, which are involved in cell survival and proliferation. The data shown in Figure 8 and File S1 indicate no difference in the expression of the aforementioned proteins in CRCs cultured with RTKIs alone (except for Lucitanib-treated CRCs), thereby suggesting that impairment of FGFR or VEGFR signaling by RTKIs was not the main factor mediating the decreased survival and proliferation of CRCs treated with Doxorubicin. Of note, Lucitanib-induced reduction in the expression of the phosphorylated form of VEGFR2 (Figure 8B) suggests that the apoptotic and anti-proliferative activities observed in single-treated cells (Figure 6A and Figure 7B, respectively) are due to impairment of the overactivated VEGF-mediated signaling cascade in HCT116 cells.

### 3.6. Receptor Tyrosine Kinase Inhibitors Have No Impact on ABC Transporters’ Expression and Subcellular Distribution

The overexpression of ABC transporters facilitating drug efflux from cancer cells is known to be one of the mechanisms of cancer chemoresistance. Multiple reports, including our own [41], illustrate RTKI’s ability to regulate the activity and function of ABC transporters via multiple mechanisms (e.g., altering their expression, modifying their subcellular localization, and inhibiting their efflux function). To test these possibilities directly, the following studies were designed.

We initially examined the expression of three major multidrug resistance (MDR) proteins (P-gp, ABCG2, and MRP-1) on both protein and translational levels in CRCs. Chemo-resistant cancer cell lines (e.g., triple-negative breast cancer HCC1806 TxR [47] and osteosarcoma SaOS-2 DoxR [46]) served as the positive control for the high expression of these transporters. Western blot data shown in Figure 9 and File S1 illustrate the high expression of ABC-related proteins in CRCs when compared with chemo-resistant cancer sublines. This was consistent with RT-PCR data showing high mRNA levels of *MDR1*, *MRP-1*, and *ABCG2* in CRCs compared with chemo-resistant-naive HCC1806 triple-negative breast cancer cells and SaoS-2 osteosarcoma cells (Table 4 and Table 5), thereby indicating high basal expression of ABC transporters in CRCs. Importantly, all three CRC lines used in the present study exhibited high *mRNA* levels of *MRP-1* compared with both chemo-resistant cancer cell lines (e.g., HCC1806 TxR breast cancer and SaOS-2 DoxR osteosarcoma cell sublines—Supplementary Tables S3 and S4), thereby suggesting high intrinsic chemoresistance of CRCs to certain chemotherapies known to be substrates for this particular ABC transporter.

Next, we examined whether RTKI-induced sensitization of CRCs to Doxorubicin was due to the downregulation of ABC transporters at the protein level. Western blot data shown in Figure 10 (*left*) and File S1 indicate that HCT116 cells exhibited similar levels of ABC-related proteins compared with untreated controls, thereby suggesting that Lucitanib and Pemigatinib cannot sensitize HCT116 cells to Doxorubicin by decreasing the expression of MDR-related proteins. Of note, a moderate decrease in MRP-1 expression was observed in Regorafenib-treated HCT116 cells, whereas the expression of MDR1 and BCRP1 remained unchanged. Similar results were obtained in Colon-26 cells (Figure 10—*right*). To exclude early receptor inhibition followed by feedback activation in cancer cells treated with RTKIs alone or in combination with Doxorubicin, we assessed the expression of phosphorylated forms of FGFR1/2 and VEGFR1 and their downstream signaling proteins, such as AKT and MAPK, at earlier time points such as 0.5, 2, 6, and 24 h. As shown in Supplementary Figure S5 and File S1, no receptor inhibition was observed during the whole time period of the experiment when compared with the non-treated controls. Of note, for some markers (e.g., pMAPK), we observed increased expression in cancer cells treated with a combination of Regorafenib and Doxorubicin compared with those treated with this RTKi alone, potentially suggesting transient activation of adaptive pro-survival mechanisms in DNA-damaged cancer cells.

Next, we assessed the subcellular distribution of MDR-related proteins in CRCs treated with RTKIs or DMSO (i.e., solvent), serving as a negative control, for 24 h. The immunofluorescence microscopy data shown in Figure 11 also showed no changes in the subcellular localization of MDR proteins in RTKI-treated HCT116 cells. Similar results were obtained in Colon-26 cells (Supplementary Figure S6).

These data, together with the absence of the downregulation of ABC transporter expression, as shown in Figure 10, suggest that impaired ABC transporter activity and decreased efflux of chemotherapeutic agents might be a predominant molecular mechanism responsible for RTKI-induced sensitization of CRC to chemotherapeutic agents used for CRC chemotherapy.

### 3.7. Receptor Tyrosine Kinase Inhibitors Decrease the Efflux of Chemotherapeutic Drugs from CRCs

To test this possibility directly and assess whether RTKIs can retain chemotherapeutic agents within CRCs, we used fluorescent chemotherapeutic agents (e.g., Doxorubicin and Mitoxantrone) and fluorescent substrates for specific ABC transporters (e.g., Calcein AM as a substrate for ABCB1). For this purpose, we measured the mean fluorescence intensity (MFI) of Doxorubicin and Mitoxantrone in HCT116 and Colon-26 cells treated with these agents in the presence of Regorafenib, Lucitanib, Pemigatinib, Infigratinib, or Cabozantinib. In this experimental setting, Tariquidar (ABCB1 inhibitor), Ko-143 (ABCG2 inhibitor), and Mk-571 (MRP-1 inhibitor) served as the positive controls.

Strikingly, 2 of the 3 aforementioned RTKIs (i.e., Pemigatinib and Lucitanib) induced a significant (>30%) increase in MFI for Doxorubicin when compared with HCT116 cells treated with Doxorubicin alone (Figure 12A, Table 6—*left panel*). Moreover, a similar pattern illustrating RTKI-mediated inhibition of chemotherapeutic efflux from colon cancer cells was observed in Mitoxantrone-treated HCT116 cells, as shown in Supplementary Figure S7 and Table S5.

Next, using Calcein AM, a well-known P-gp substrate, we revealed that this MDR protein is a molecular target for Pemigatinib and Lucitanib. Indeed, these RTKIs effectively inhibited the efflux of Calcein AM from HCT116 (Supplementary Figure S8 and Table S6). As expected, we found that Tariquidar produced a maximal increase in Calcein AM-mediated MFI, whereas Mk-571 and Ko-143 were almost ineffective at altering the MFI parameters of Calcein AM in these CRCs, thereby illustrating that the ability of the aforementioned RTKIs to sensitize CRCs to Doxorubicin was predominantly due to the inhibition of its efflux via the ABCB1 transporter.

Given that Infigratinib and Cabozantinib were found to be the most effective RTKIs for sensitizing Colon-26 cells to Doxorubicin (Figure 6), we tested whether this was also due to decreased Doxorubicin efflux from this cell line. Indeed, we observed a similar pattern for Colon-26 cells treated with Doxorubicin in combination with RTKIs, as shown in Figure 12B and the *right panel* of Table 6. Thus, consistent with the apoptosis data shown in Figure 6, Infigratinib and Cabozatinib induced a prominent accumulation of Doxorubicin within cancer cells, suggesting that RTKI-induced impairment of Doxorubicin efflux from cancer cells might be a predominant mechanism underlying RTKI-induced sensitization of CRCs to this particular chemotherapeutic agent.

Of note, Mk-571, the MRP-1 inhibitor, was ineffective across all the aforementioned experimental conditions, consistent with the Western blot data shown in Figure 9, which illustrates low expression of this particular ABC transporter in CRCs.

The results from the competition assay using Tariquidar (ABCB1 inhibitor) and Ko-143 (ABCG2 inhibitor) demonstrated that the MFI of both Doxorubicin and Mitoxantrone increased with the Tariquidar–Infigratinib combination, thereby reducing the release of the chemotherapeutic agents relative to the substrate alone (Table 7). The values of this parameter were similar when Tariquidar was used alone and with Infigratinib for both substrates, Doxorubicin and Mitoxantrone (Table 7). This means that the combination did not exert a stronger effect on intracellular accumulation because it inhibited the common protein ABCB1. On the other hand, the combined use of Ko-143 and Infigratinib significantly increased the MFI of the substrates (Table 7). The MFI values for Doxorubicin or Mitoxantrone obtained using this combination were approximated to those for Infigratinib, Tariquidar alone, and their combination, as Ko-143 did not have a particular influence on this parameter (Table 7). These results showed that Infigratinib inhibited ABCB1 transporter activity in the Colon-26 cancer cell line. As a result, the intracellular accumulation of substrates for these proteins increased, allowing these chemotherapeutic drugs to exert their influence effectively.

Of note, for the transporter assays indicated above, the RTKIs were used at 20 µM and co-treated with 40 µM Doxorubicin or Mitoxantrone, according to well-established protocols used by our lab and others [41,46,72,73,74,75,76]. Even though the time period for these assays was less than 6 h and had no impact on the cellular viability evidenced by bright-field microscopy, to exclude the impact of the cytotoxicity of the aforementioned combinations, we assessed the expression of apoptotic markers (e.g., cleaved PARP and caspase-3) in HCT116 cells treated with Doxorubicin or Mitoxantrone alone or in the presence of RTKIs. As expected, we found no evidence of apoptosis in cancer cells across all experimental conditions, suggesting that the combinations of RTKIs and DNA topoisomerase inhibitors are not toxic (Supplementary Figure S9). Additionally, no significant changes in cell cycle profile were found in RTKI-treated colon cancer cells when compared with non-treated controls or after Doxorubicin treatment (Supplementary Table S7), thereby suggesting that the changes in fluorescence intensity of ABC substrates used in the present study (e.g., Doxorubicin, Mitoxantrone, and Calcein AM) in cancer cells cultured in the presence of RTKIs were not due to the decreased cellular viability.

### 3.8. Infigratinib Potentiates the Cytotoxic Activity of Doxorubicin In Vivo

To corroborate the in vitro studies, we examined whether Infigratinib used alone or in combination with Doxorubicin exhibited antitumor activity against Colon-26 allografts. For this purpose, Colon-26 cells were injected into the flanks of female Balb-c mice, and tumors were allowed to grow for at least 2 weeks before single (e.g., Infigratinib or Doxorubicin) or dual treatment. Although both chemotherapies exhibited moderate antitumor activity against Colon-26, their combination significantly reduced the weight and volume of Colon-26 allografts over 3 weeks of treatment compared with the baseline (Figure 13A,B). This correlated with the decreased number of viable cancer cells in this experimental group, as evidenced by hematoxylin–eosin staining (Figure 13C, *upper panel*). We also observed an increase in cleaved caspase-3 expression in Colon-26 allografts treated with a combination of Doxorubicin and Infigratinib (Figure 13C, *middle panel*). As expected, a significant decrease in the number of proliferative (i.e., Ki-67-positive) cells was observed in Colon-26 allografts treated with a combination of Infigratinib and Doxorubicin, compared with non-treated controls or with experimental groups treated with either a single RTKI or Doxorubicin (Figure 13C, *bottom panel*). Based on these results, we concluded that Infigratinib, a selective pan-FGFR inhibitor, effectively re-sensitized Colon-26 colon carcinoma cells in vivo.

### 3.9. In Silico Investigation of RTKI Interaction with ABC Transporters Reveals Molecular Mechanism of Their “Off-Target” Effects

Lastly, we performed molecular docking analysis to identify potential binding sites of the aforementioned RTKI on two major ABC transporters upregulated in CRCs—ABCB1 and ABCG2. High-resolution, occluded conformation bound to Tariquidar and of human origin was the most important parameter for selecting the structure of the ABCB1 transporter (PDB ID: 7A6E). Before studying RTKI, we validated the docking protocol. For this purpose, we removed the protein ligands, re-docked Tariquidar, and saved all grid and docking parameters for use with FGFR and VEGFR signaling inhibitors. Based on the data presented in Table 8, Cabozantinib, Infigratinib, and Regorafenib showed the lowest Estimated Free Energy of Binding with ABCB1 among all RTKIs (−7.86, −6.95, and −6.83 kcal/mol, respectively). This docking score was not lower than that of specific ABC transporter inhibitors (−8.34 kcal/mol) but confirmed the strong connection of the aforementioned inhibitors with this protein. That was consistent with the FACS analysis, in which two of them (Cabozantinib and Infigratinib) significantly increased the intracellular accumulation of Doxorubicin in the Colon-26 murine colon adenocarcinoma (Figure 12B and Table 6, *right panel*).

Moreover, a similar drug-binding pocket in the transmembrane domain (TMD) of ABCB1 was predicted to be an RTKI-binding site. This data was in concordance with known drug-binding cavity residues [77]. All inhibitors interacted with almost the same amino acid residues, as shown in Table 9. The most important amino acids for Cabozantinib and Infigratinib included LEU65, PHE336, ILE340, PHE983, and MET986, which were involved in the most significant chemical interactions (Figure 14). For example, most interactions in the Infigratinib structure were with the 3,5-dimethoxyphenyl group bearing two chlorides (Figure 14A), whereas in Cabozantinib, the interactions were with the quinoline ring and the 4-fluorophenyl group (Figure 14B).

Ko-143 was used for the preparation of the docking protocol for the ABCG2 transporter (PDB ID: 6FEQ), which is characterized by an inward-facing conformation bound with Ko-143, human origin and high resolution, as well as the ABCB1 protein. Molecular docking analysis of Ko-143 showed low Estimated Free Energy of Binding (−6.52 kcal/mol), but docking scores obtained for some RTKIs, e.g., Cabozantinib, Lucitanib, and Pemigatinib, were even lower than the value for this specific ABCG2 inhibitor (−7.29, −6.88, and −6.53 kcal/mol, respectively), as shown in Table 8. ABCG2 protein is characterized by a symmetrical structure with two identical chains. All ABC transporter inhibitors and RTKI were bound to both of them in the TMD, forming the drug-binding cavity composed of similar amino acids (Table 9). As well as for binding with ABCB1, the quinoline ring of Cabozantinib and Lucitanib was connected with the TMD of ABCG2 (Figure 15A,B), whereas the difluoride 3,5-dimethoxyphenyl group in Pemigatinib showed more interactions with this transporter (Figure 15C).

Collectively, we show here, for the first time, the “off-target” effects of several FGFR/VEGFR inhibitors on multidrug-resistant CRCs. This included Pemigatinib and Infigratinib (selective FGFR inhibitors), Cabozantinib, Regorafenib, and Lucitanib (multi-kinase inhibitors targeting multiple signaling pathways, including FGFR and VEGFR). Most of these inhibitors were structurally similar (Figure 1A,B), which could contribute to their most effective binding to various members of the ABC transporter family, particularly ABCB1 or ABCG2, and thus lead to changes in the functional activity of the aforementioned proteins by blocking the pumping function. These effects were due to the RTKIs’ ability to inhibit ABCB1-mediated efflux of chemotherapeutic agents (e.g., Doxorubicin, Mitoxantrone, and Calcein AM) from CRCs. At the same time, these RTKIs did not alter ABC transporter expression at the transcriptional and translational levels or affect their subcellular distribution. Importantly, the ability of the aforementioned RTKI to sensitize CRCs to conventional therapies and induce their apoptosis was not associated with inhibition of the FGFR or VEGFR signaling pathways, revealing their “off-target” effect on CRCs, which was primarily mediated by inhibition of P-gp-induced efflux of chemotherapeutic agents from CRC cells.

## 4. Discussion

Despite significant improvements in the early diagnosis and treatment of colorectal cancer (CRC), the prognosis for metastatic CRC (mCRC) remains unfavorable [78]. Indeed, objective responses to combination therapies, including the antimetabolite 5-Fluorouracil combined with either the DNA-adduct-forming agent oxaliplatin (i.e., FOLFOX) or the DNA topoisomerase I inhibitor irinotecan (i.e., FOLFIRI), are observed in only 50% of patients [79,80]. This, in part, can be due to the development of multidrug resistance (MDR) in tumors to structurally similar or even different chemotherapeutic agents, thereby decreasing treatment efficacy. Thus, there is an urgent need to develop novel, effective therapies that overcome MDR and increase therapeutic response rates in CRC.

Given that overexpression of ABC transporters is a major mechanism of intrinsic (i.e., primary) resistance in cancer to chemotherapeutic agents, facilitating the efflux of these drugs from cancer cells, we tested whether RTKIs could sensitize CRCs to chemotherapies by regulating the activity of MDR-related proteins. Indeed, we showed here that several RTKIs targeting multiple signaling pathways, including FGFR- and VEGFR-mediated cascades, can enhance the sensitivity of CRCs to certain chemotherapeutic agents, including the DNA topoisomerase inhibitors Doxorubicin and Mitoxantrone, as well as the antimetabolite 5-Fluorouracil. Indeed, HCT116 cells treated with Doxorubicin in combination with Regorafenib, Pemigatinib, or Lucitanib exhibited a dramatic decrease in cellular viability compared with CRCs treated with chemotherapeutic agents or these RTKIs alone (Table 1 and Table 2, Figure 3 and Figure 4). This was due to enhanced apoptosis in HCT116 cells treated with the aforementioned combinations of RTKIs and Doxorubicin (Figure 6). Moreover, these combinations decreased the proliferative activity of HCT116 cells compared with untreated controls or single-treated cells (Figure 7). These data were also consistent with high synergy scores (SC) between RTKIs and Doxorubicin in HCT116 colon cancer cells (Figure 5A–D, Table 3). These RTKI effects were not limited to HCT116 cells but also extended to the Colon-26 murine adenocarcinoma cell line and the RKO human colorectal cancer cell line. As expected, the types of RTKIs sensitizing these CRCs to chemotherapeutic agents partially overlapped, and different combinations of small-molecule inhibitors and chemotherapeutic agents effectively reduced viability and proliferative activity in these CRC lines. In particular, as with HCT116 cells, Pemigatinib effectively sensitized Colon-26 cells to Doxorubicin, whereas Regorafenib was ineffective (Table 1 and Table 2; Supplementary Figures S2 and S3). Meanwhile, and in contrast to HCT116 cells, Infigratinib and Cabozantinib exhibited potent chemosensitizing activities in Doxorubicin-treated Colon-26 cells (Table 1 and Table 2, Figure 5E–H, Supplementary Figures S2 and S3). Of note, the vast majority of RTKIs used in the present study were ineffective in 5-Fluorouracil-treated HCT116 and Colon-26 cancer cell lines, whereas we did observe high potency of the selective (Infigratinib and Pemigatinib) and non-selective (Cabozantinib, Lucitanib and Regorafenib) inhibitors to sensitize these particular colon cancer cell lines to this antimetabolite (Table 1 and Table 2). In contrast to HCT116 and Colon-26 cell lines, no RTKI-mediated chemosensitizing effect was observed in Doxorubicin-treated RKO cells (Table 1 and Table 2). Overall, these data illustrate the ability of RTKI to potentiate the cytotoxic activities of certain chemotherapies across various CRCs and highlight the importance of individual variations in the types of RTKI and chemotherapeutic agents in different CRC lines. In particular, these differences might be due to a broad spectrum of molecular mechanisms, including intrinsic resistance to chemotherapeutic agents, differences in ABC transporter expression (Figure 9), and the presence of cancer stem cells. And, of course, the differences in RTK profiles, including FGFR and VEGFR signaling pathways, observed between these CRC lines (Figure 2) can also be taken into account.

Next, we provided direct evidence about the RTKIs to increase the intracellular concentrations of chemotherapeutic agents with autofluorescence moieties (e.g., Doxorubicin and Mitoxantrone) in CRCs (Figure 12, Table 6, Supplementary Figure S7, Supplementary Table S5), therefore suggesting that decreased activity of the overexpressed ABC transporters and an impaired efflux of chemotherapeutic agents from cancer cells can be a major molecular mechanism of RTKI-induced chemosensitization in CRC. Indeed, the aforementioned effects of RTKIs were not attributable to decreased expression and/or subcellular redistribution of three major ABC transporters (e.g., P-gp, MRP-1, and BCRP) (Figure 10 and Figure 11, Supplementary Figure S6).

Notably, using competitive assays with selective ABCB1 and ABCG2 inhibitors, we identified the ABCB1 protein as a primary target for Infigratinib (Table 7). This was consistent with our previous study, which identified this type of ABC transporter as a predominant molecular target for the aforementioned selective FGFR inhibitor [41]. Molecular docking analysis revealed that the “off-target” effect of this RTKI was attributable to its effective binding to the ABCB1 transmembrane domain (Figure 14). Of note, the non-additive effect of Infigratinib and Tariquidar can be interpreted as evidence that both act on the same transporter, but it could also result from assay saturation or overlapping downstream effects. Importantly, both Tariquidar and Ko-143 increased Doxorubicin fluorescence, suggesting that multiple transporters may contribute to this process.

Overall, we show here for the first time that some RTKIs originally developed to target FGFR and/or VEGFR pathways can effectively sensitize CRCs to certain chemotherapies, including DNA topoisomerase inhibitors. Importantly, this sensitizing effect was not related to the inhibition of the aforementioned signaling cascades, subcellular distribution of ABC transporters, dysregulation in the cell cycle, or changes in membrane permeability.

Lastly, we showed that RTKIs can potentiate the cytotoxic effects of chemotherapy in CRCs in vivo. For this purpose, we used Infigratinib, a selective FGFR inhibitor, which has previously been shown to be an effective chemosensitizing agent for Colon-26 murine carcinoma cells in vitro. Data shown in Figure 13 illustrate the high potency of this inhibitor in Doxorubicin-treated CRC allografts. This was evidenced by reduced growth of Colon-26 colon cancer allografts with a combination of Infigratinib and Doxorubicin. Immunohistochemical (IHC) staining revealed a substantial increase in apoptotic cells (e.g., cleaved caspase-3-positive) in allografts from animals receiving the combined therapy of Infigratinib and Doxorubicin compared with the single-treated groups. Conversely, the number of proliferative cells (e.g., Ki-67-positive cells) was significantly reduced after such combined therapy.

Thus, these data are consistent with multiple reports indicating that the “off-target” effects of small-molecule inhibitors targeting various signaling cascades can effectively potentiate the cytotoxic activities of chemotherapies by promoting the retention of chemotherapeutic agents within cancer cells, thereby increasing the efficacy of anticancer treatment. For example, Wu C.-P. and co-authors demonstrate the high potency of Erdafitinib, a small-molecule pan-FGFR kinase inhibitor, in sensitizing ABCB1-overexpressing cancer cells to certain chemotherapeutic drugs, including mitotic-poisoning agents (e.g., Paclitaxel and Vincristine) and Topotecan [81]. Additionally, Kim S.H. et al. demonstrated that Paclitaxel, in combination with Infigratinib, synergistically suppressed urothelial carcinoma cell migration and colony formation by regulating EMT-associated factors, while FGFR1 knockdown enhanced the antitumor effect of Paclitaxel [82]. Thus, RTKIs are currently considered potent chemosensitizing agents that increase the sensitivity of cancer cells to certain chemotherapies via modulation of ABC transporter activity [83,84,85].

Importantly, the concentrations of RTKIs used in our study are pharmacologically achievable. In particular, this includes Infigratinib [49,50], Pemigatinib [51,52,53], Lucitanib [55,56,57], Cabozantinib [60,61,62,63], and Regorafenib [64,65,66,67], thereby making them attractive RTKIs to overcome chemoresistance in CRC.

## 5. Conclusions

Overall, the role of RTKIs in the modulation of ABC transporter-mediated MDR phenotypes in cancer has attracted significant attention in recent decades and has been extensively discussed in multiple original manuscripts and several comprehensive reviews, including our own, to demonstrate their potent “off-target” effects to inhibit the efflux of chemotherapies from human malignancies and therefore overcome primary and secondary chemoresistances [27,77,83,84,85]. We show here, for the first time, that some RTKIs targeting FGFR and/or VEGFR signaling pathways can sensitize certain CRC lines (e.g., HCT 116 and Colon-26) to chemotherapeutic agents (e.g., Doxorubicin) by inhibiting their efflux, thereby supporting a rationale for combined therapies for CRCs.

## Figures and Tables

**Figure 1:**
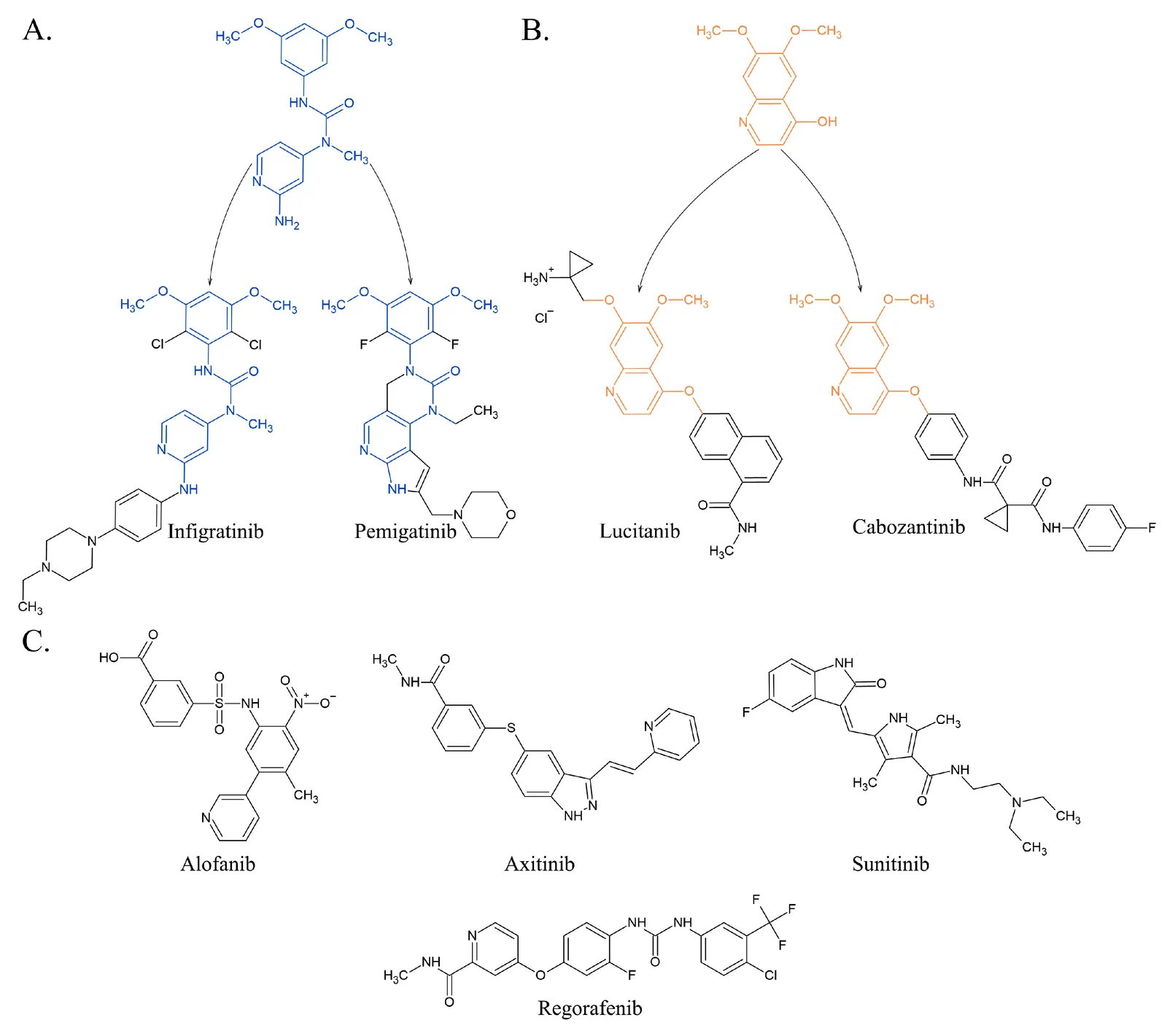
2D structures of the studied receptor tyrosine kinase inhibitors are divided into 3 groups depending on the structural similarity. Infigratinib and Pemigatinib (**A**) and Lucitanib and Cabozantinib (**B**) have the same scaffold (blue and orange colors indicate the same core for each group). Alofanib, Axitinib, Sunitinib, and Regorafenib (**C**) do not have a common core.

**Figure 2:**
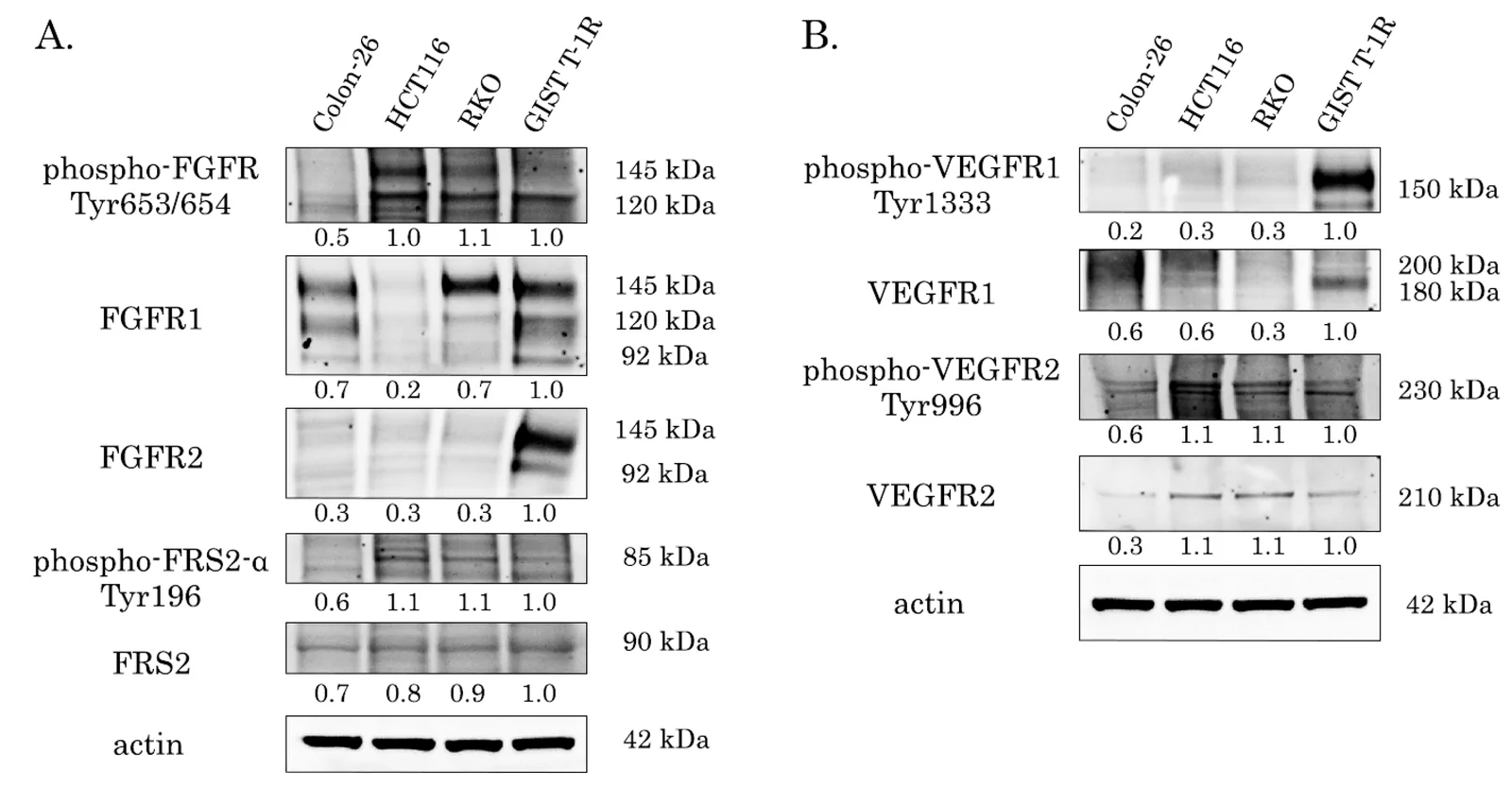
Assessment of activation of FGFR and VEGFR signaling pathways in 3 CRC lines (Colon-26, HCT116, and RKO) by Western blotting. GIST T-1R cells were used as a positive control. (**A**) Expression of total and phosphorylated forms of FGFR1/2 and the adaptor protein FRS-2 in 3 CRC lines and GIST T-1R cells. An actin stain was used as a loading control. (**B**) Expression of total and phosphorylated VEGFR1/2 in the aforementioned cancer cell lines. An actin stain was used as a loading control. The uncropped blots are shown in File S1.

**Figure 3:**
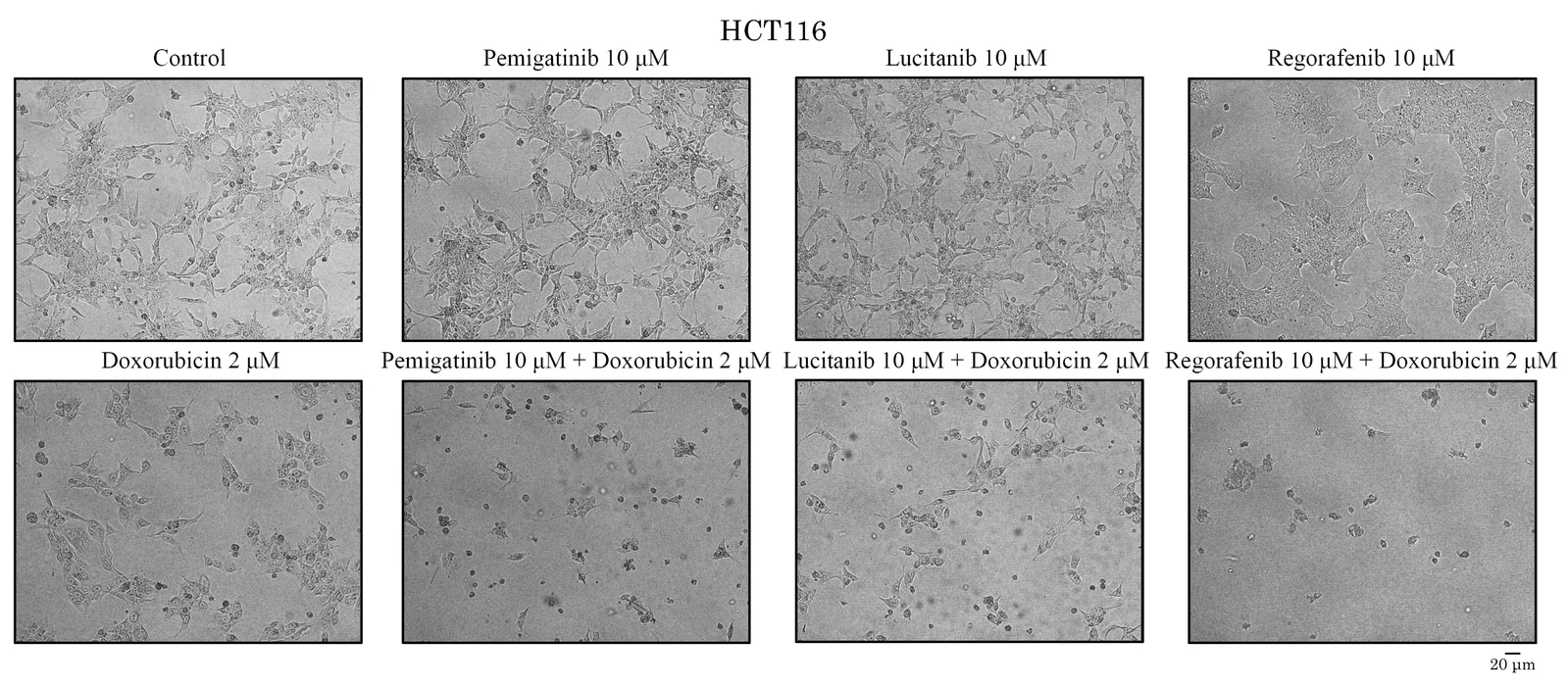
Representative bright-field images of HCT116 colon cancer cells after exposure to DMSO (control), Doxorubicin (2 µM), RTKIs (Pemigatinib, Lucitanib, and Regorafenib—10 µM), and their combinations for 72 h. Combinational treatment significantly reduces cell confluency when compared with monotherapies and control.

**Figure 4:**
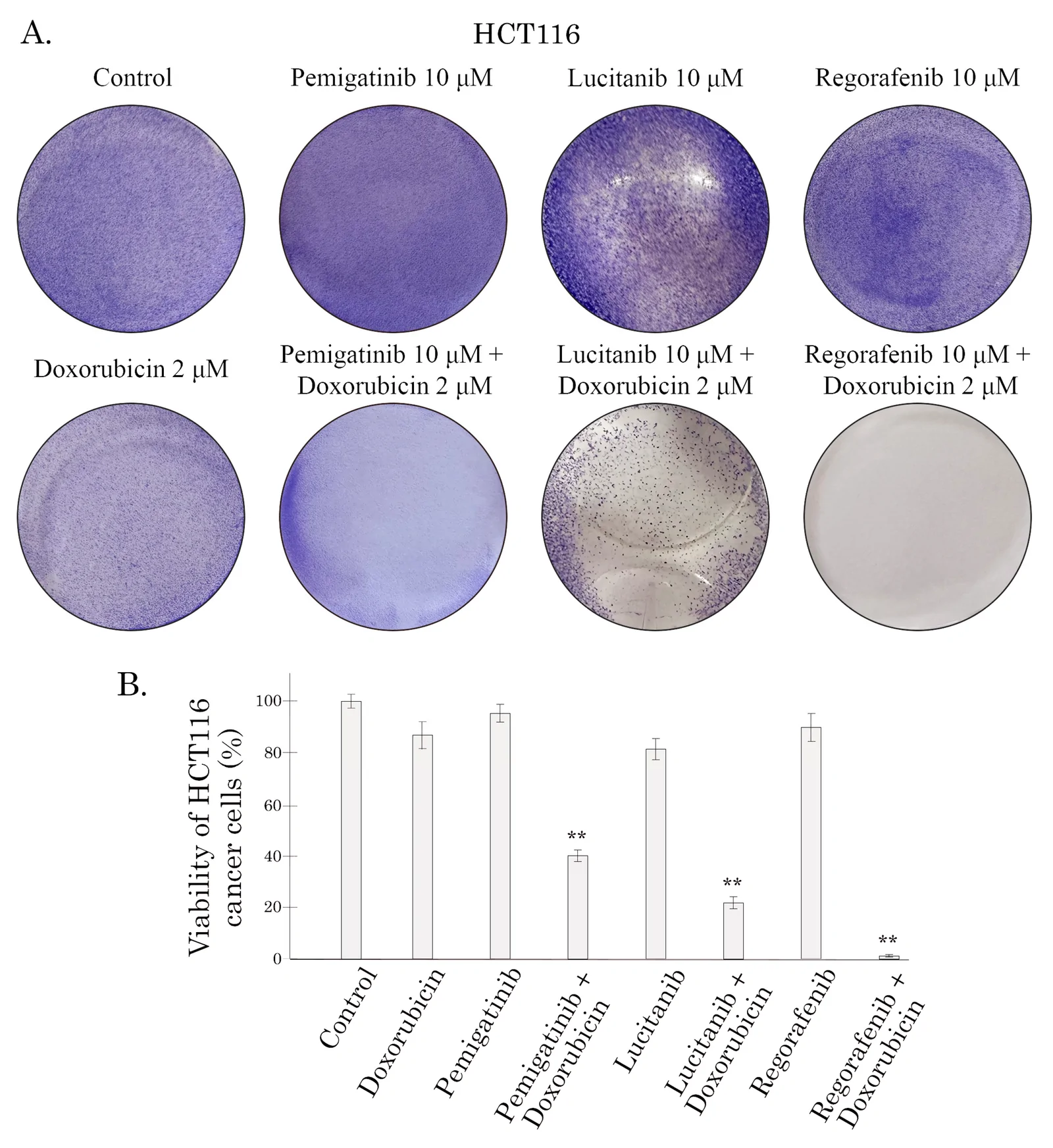
Cristal violet staining of HCT116 cancer cells treated with DMSO (control), Doxorubicin (2 µM), and RTKIs (i.e., Pemigatinib, Lucitanib, and Regorafenib—10 µM) alone for 72 h, and when combined with Doxorubicin. (**A**) Representative images of HCT116 cancer cells stained with crystal violet solution after 72 hours of incubation with the aforementioned chemotherapeutic drugs. (**B**) Quantitative analysis of HCT116 cells stained with crystal violet solution. Control was taken as 100% of cellular viability. Data were presented as mean (%) ± SD (*n* = 3). Significant differences compared to control at *p* < 0.01 (**).

**Figure 5:**
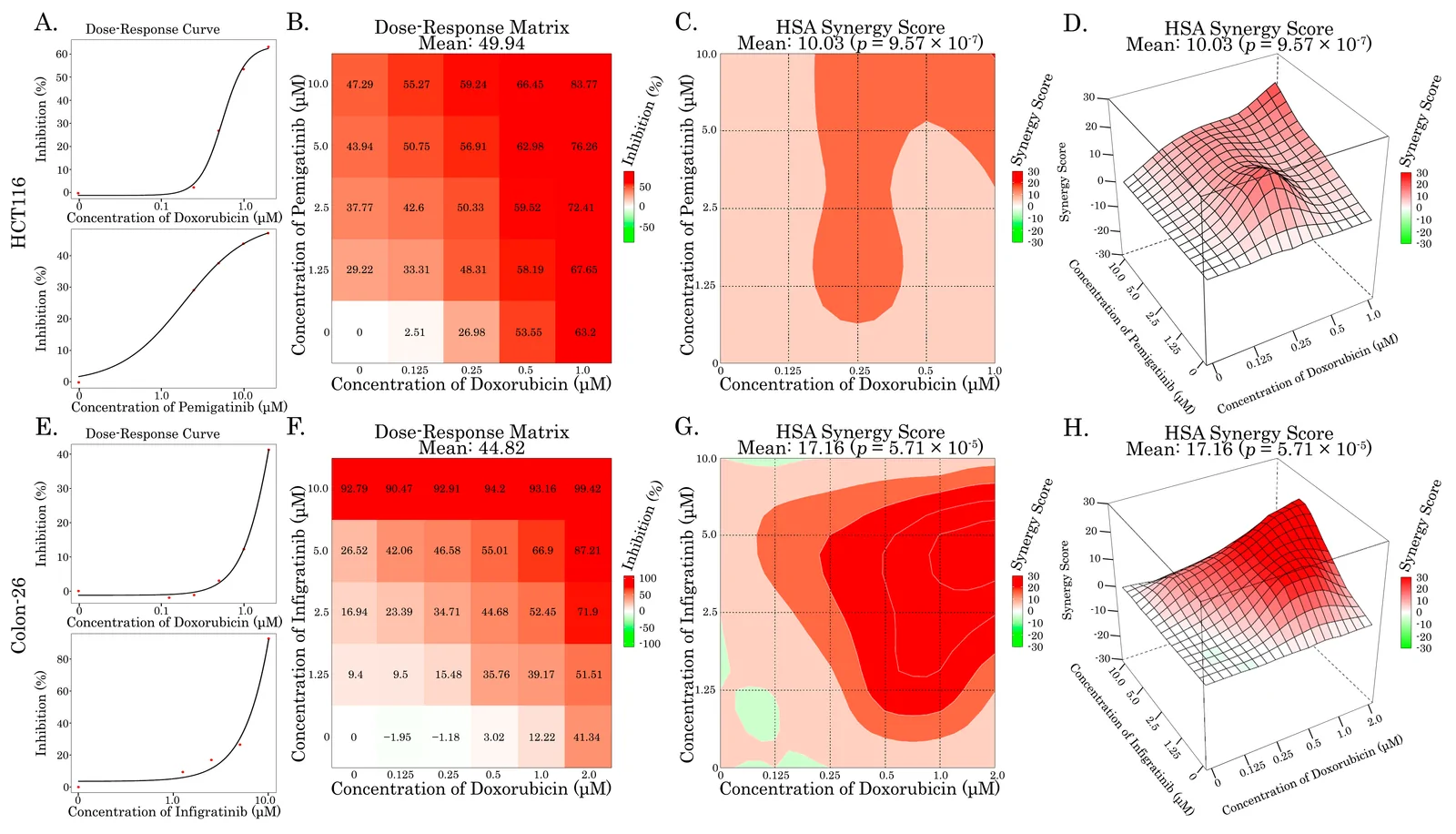
Analysis of synergy between Doxorubicin and Pemigatinib in HCT116 (**A**–**D**) and between Doxorubicin and Infigratinib in Colon-26 cells (**E**–**H**). CRCs were treated with increasing concentrations of the aforementioned RTKI, Doxorubicin, and their combinations to assess synergistic, additive, or antagonistic effects using the online SynergyFinder tool. (**A**,**E**) Single-agent curves demonstrating a dose-dependent cytotoxic effect in CRCs treated with RTKIs and Doxorubicin. The data represent the mean of three independent experiments. Red dots represent cell viability (%) at specific concentrations of the chemotherapeutic agent. (**B**,**F**) Dose-dependent matrices of RTKI and Doxorubicin combinations to show the maximal cytotoxicity of both drugs used at higher concentrations. (**C**,**G**) Two-dimensional synergy maps illustrate robust synergism between RTKI and Doxorubicin, with HSA scores of 10.03 for HCT116 cells and 17.16 for Colon-26 cells. (**D**,**H**) Three-dimensional HSA synergy landscapes illustrating high HSA scores and confirming the combinational benefit of RTKI and Doxorubicin in CRCs.

**Figure 6:**
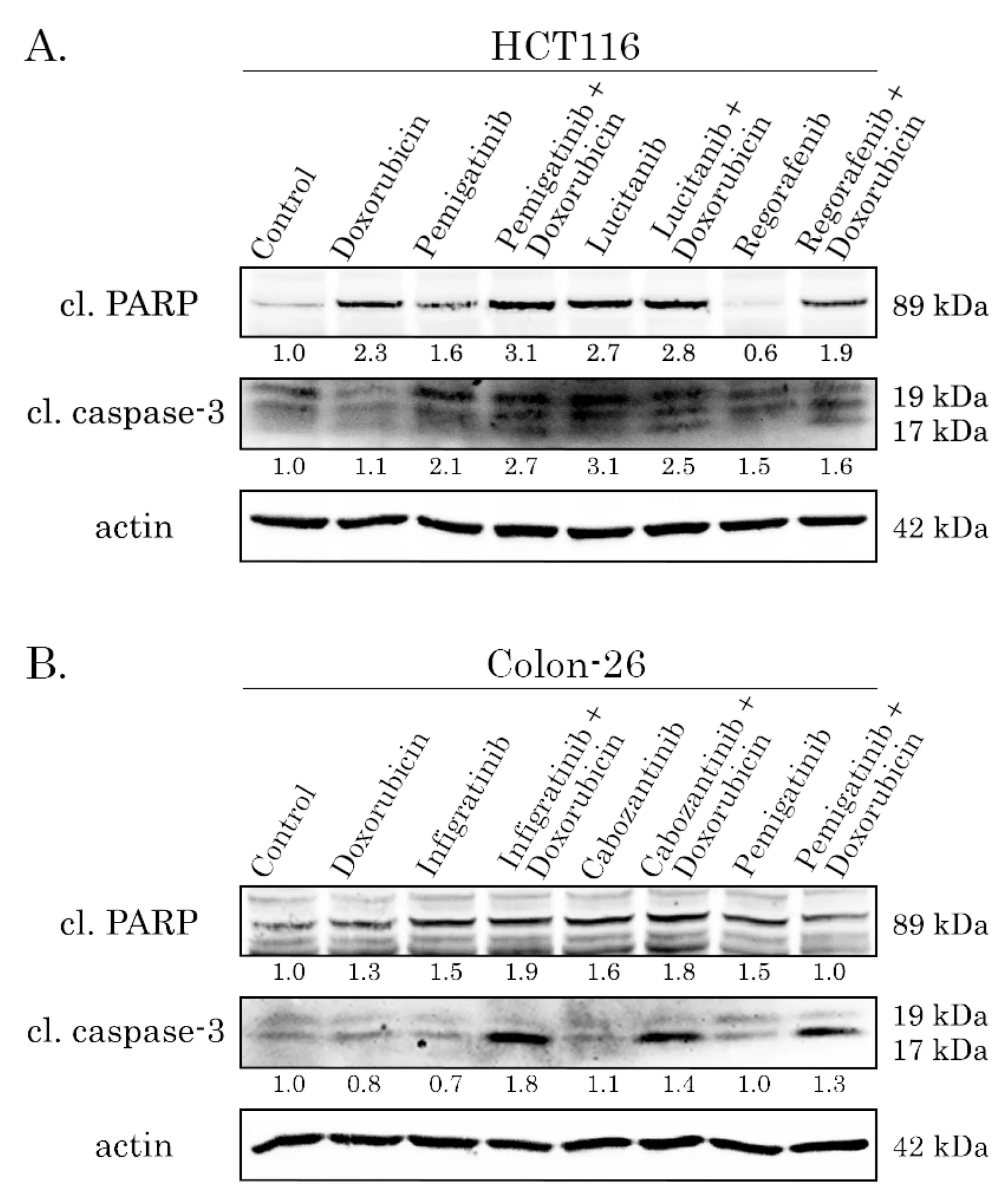
Pro-apoptotic activity of RTKIs, Doxorubicin alone, and in combination in CRC lines. HCT116 (**A**) and Colon-26 (**B**) cells were treated with Doxorubicin (2 µM or 1 µM in HCT116 and Colon-26 cells, respectively), Pemigatinib, Lucitanib, Regorafenib, Cabozantinib, and Infigratinib (10 µM) alone or in combination for 72 h and subjected to Western blot analysis to examine the expression of apoptotic markers, including cleaved poly-(ADP)-ribose-polymerase (PARP) and caspase-3. Actin staining was used to show the comparable amounts of protein loaded into each sample. The uncropped blots are shown in File S1.

**Figure 7:**
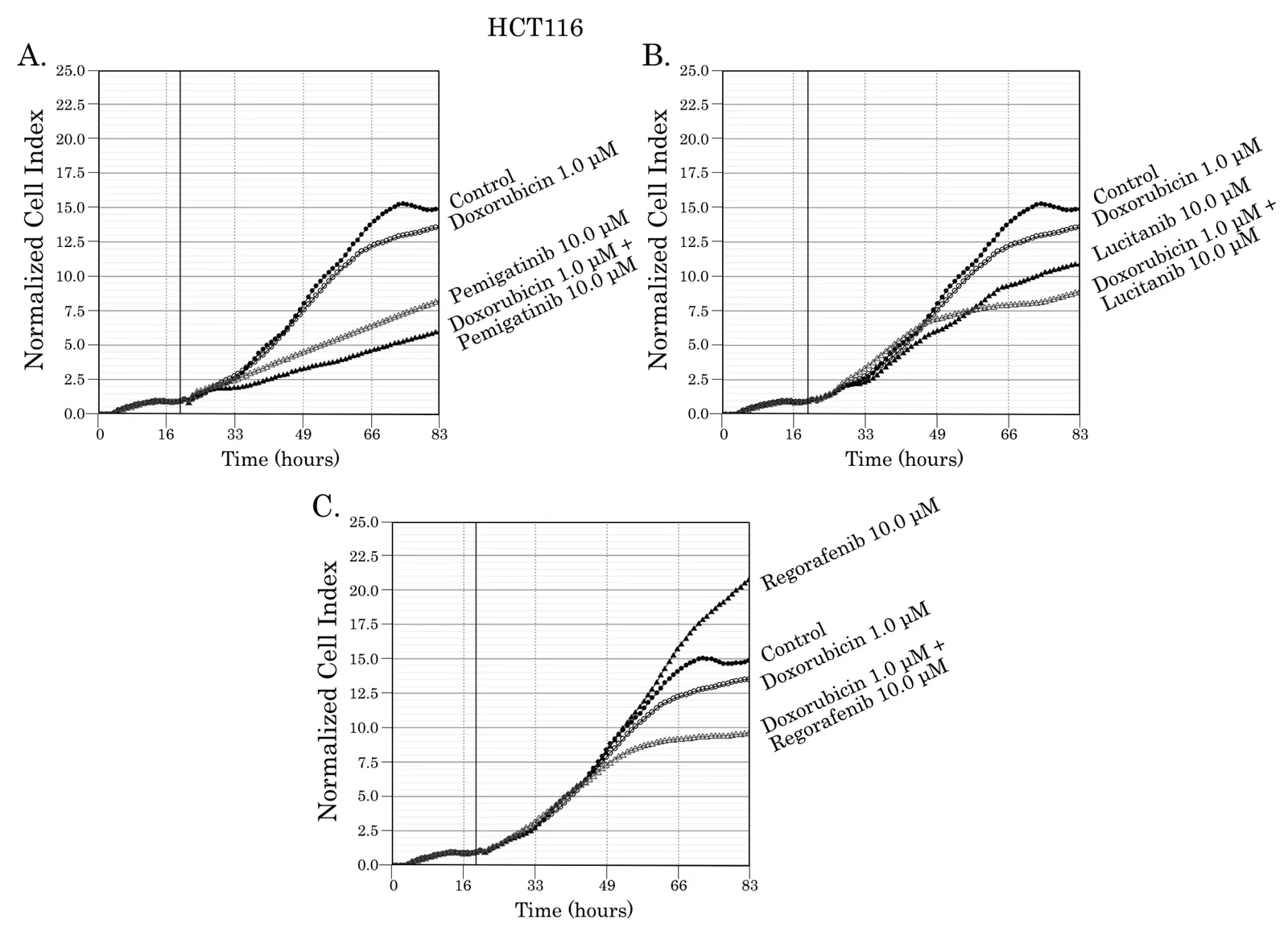
Anti-proliferative effects of RTKIs used alone or in combination with Doxorubicin in the HCT116 colon cancer cell line. Changes in the growth kinetics of cancer cells treated with the solvent DMSO (control), Doxorubicin, Pemigatinib (**A**), Lucitanib (**B**), and Regorafenib (**C**), alone or in combination. Cells (0.5 × 10^5^/mL) were seeded into the wells of an E-Plate L8 PET cassette and installed in the iCELLigence cell growth kinetics system (ACEA Biosciences, San Diego, CA, USA). Cells were allowed to attach and grow for the following 24 h. Afterward, RTKI, Doxorubicin, or DMSO was introduced into the cell culture (the vertical line). Cell proliferation index values were recorded every hour throughout the experiment. RTCA Software version 1.0 (ACEA Biosciences, Inc., San Diego, CA, USA) was used to analyze the data.

**Figure 8:**
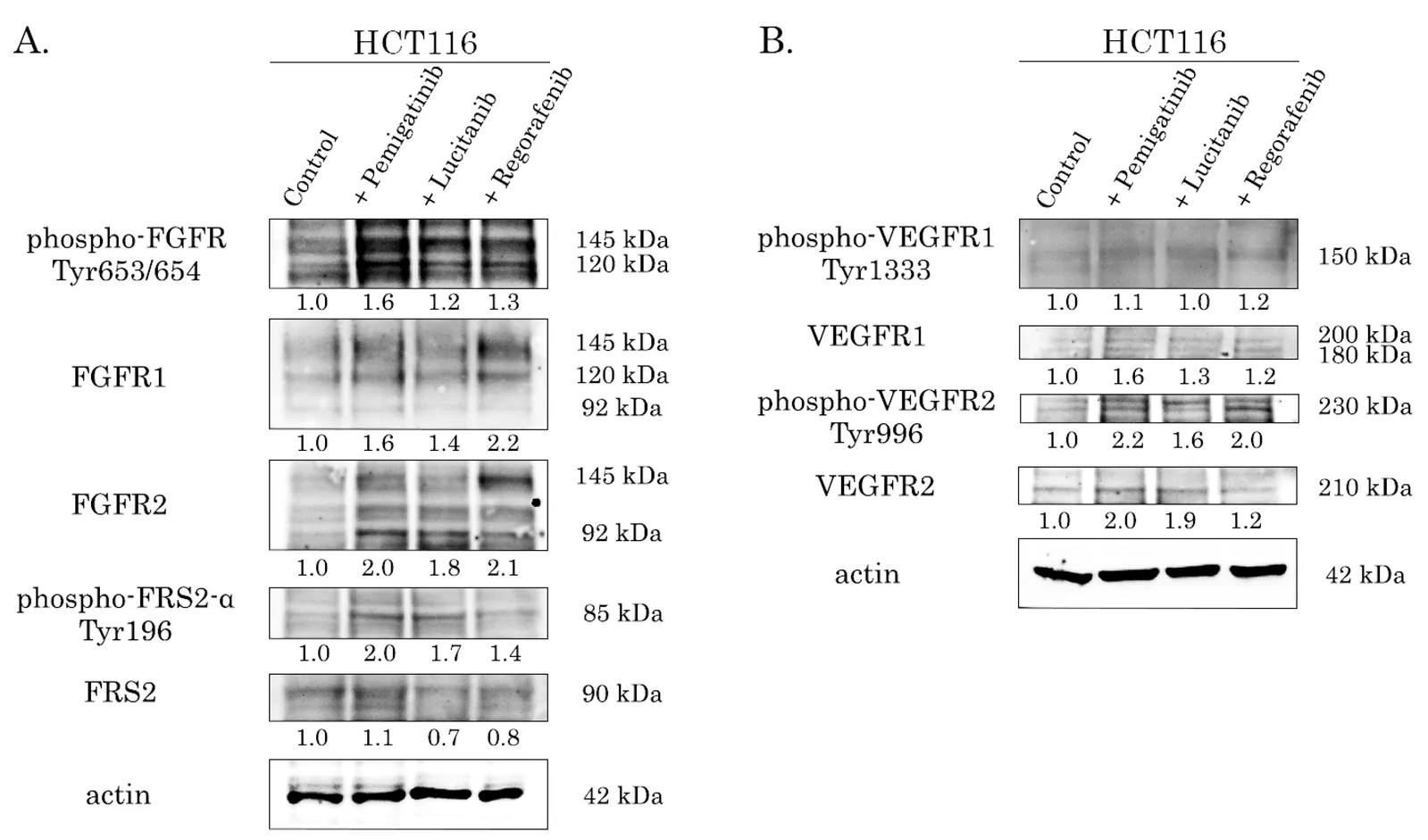
An impact of RTKI (Pemigatinib, Lucitanib, and Regorafenib) on FGFR and VEGFR signaling pathways in the HCT116 cancer cell line. Changes in expression of total and phosphorylated forms of FGFR and VEGFR signaling proteins ((**A**) and (**B**), respectively) in HCT116 cells in response to RTKI (10 µM) for 72 h. Actin stain was used as a loading control for all experiments shown in (**A**,**B**). The uncropped blots are shown in File S1.

**Figure 9:**
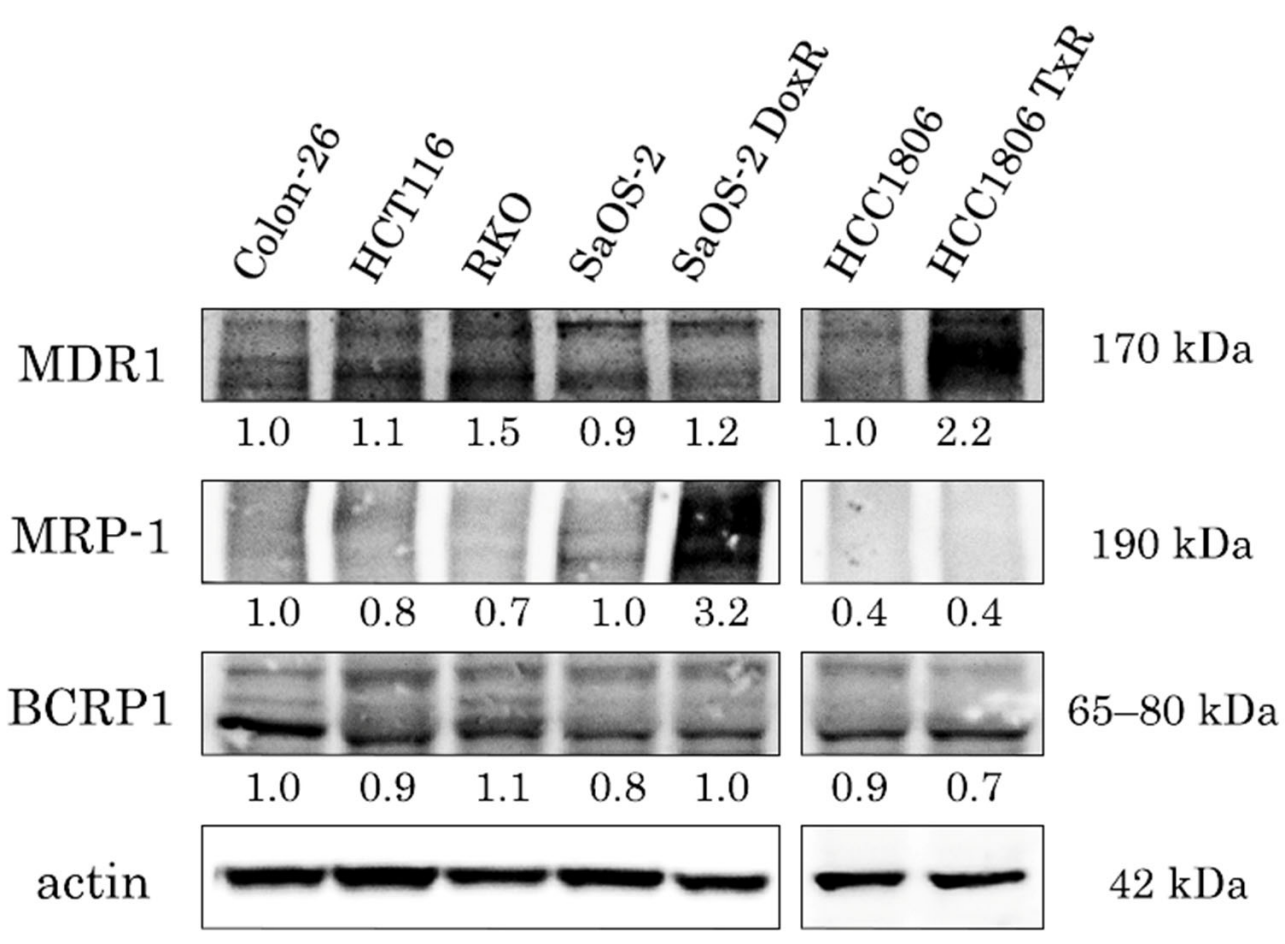
The expression of ABC transporters (MDR1, MRP-1, and BCRP1) in Colon-26, HCT116, and RKO colorectal cancer cells. Chemoresistant SaOS-2 (e.g., SaOS-2 DoxR) osteosarcoma cells and HCC1806 triple-negative breast cancer cells (HCC1806 Tx-R) were used as positive controls for elevated ABC-transporter levels, whereas their parental cell lines (SaOS-2 and HCC1806) served as negative controls. Actin stain was used as a marker for equal protein loading. The uncropped blots are shown in File S1.

**Figure 10:**
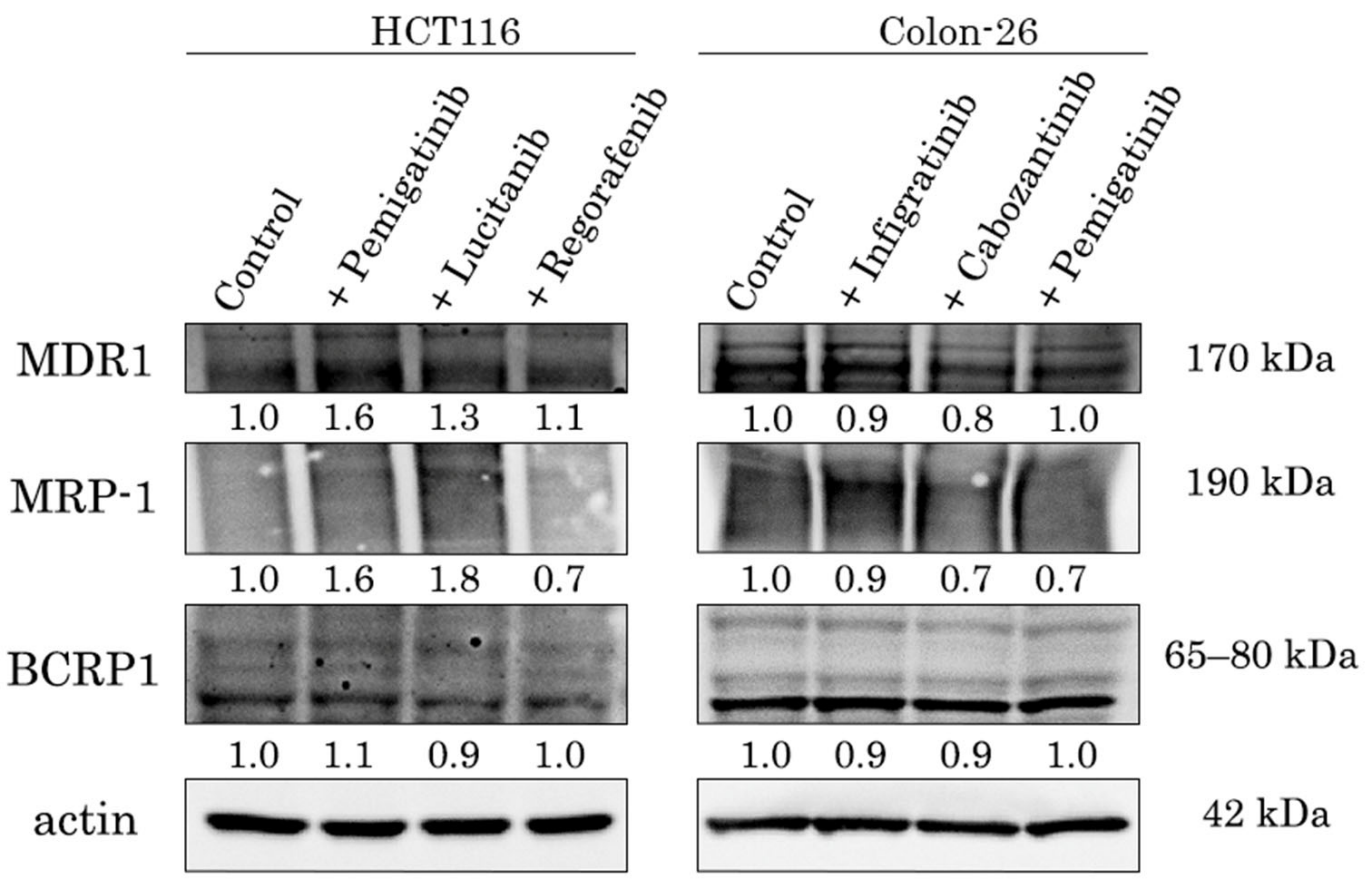
Expression of ABC transporters in HCT116 and Colon-26 colon cancer cells treated with RTKI. Western blot data show no effect of RTKI (e.g., Infigratinib, Cabozantinib, Pemigatinib, Lucitanib, or Regorafenib) on the expression of MDR-related proteins (MDR1, MRP-1, and BCRP1) in HCT116 and Colon-26 cells treated with the aforementioned RTKIs (10 µM) for 72 h. Actin stain was used as a loading control. The uncropped blots are shown in File S1.

**Figure 11:**
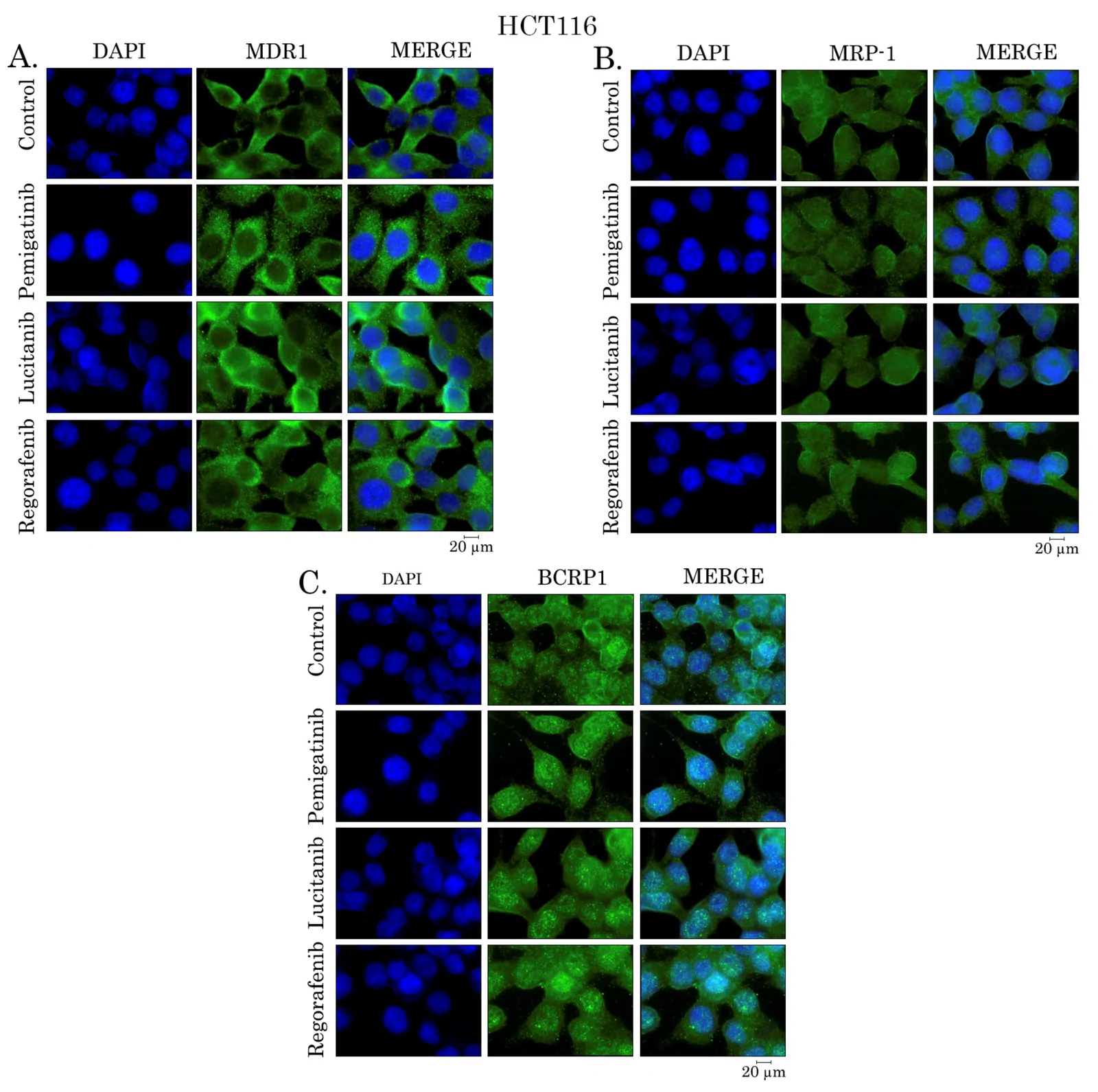
RTKIs did not influence the subcellular localization of MDR1 (**A**), MRP-1 (**B**), and BCRP1 (**C**) in HCT116 cells. Representative immunofluorescence images of HCT116 cancer cells incubated with DMSO (control) or Pemigatinib, Lucitanib, and Regorafenib at 10 µM for 24 h are shown. Cell nuclei were outlined by staining with DAPI. Magnification 100×, scale bars 20 μm.

**Figure 12:**
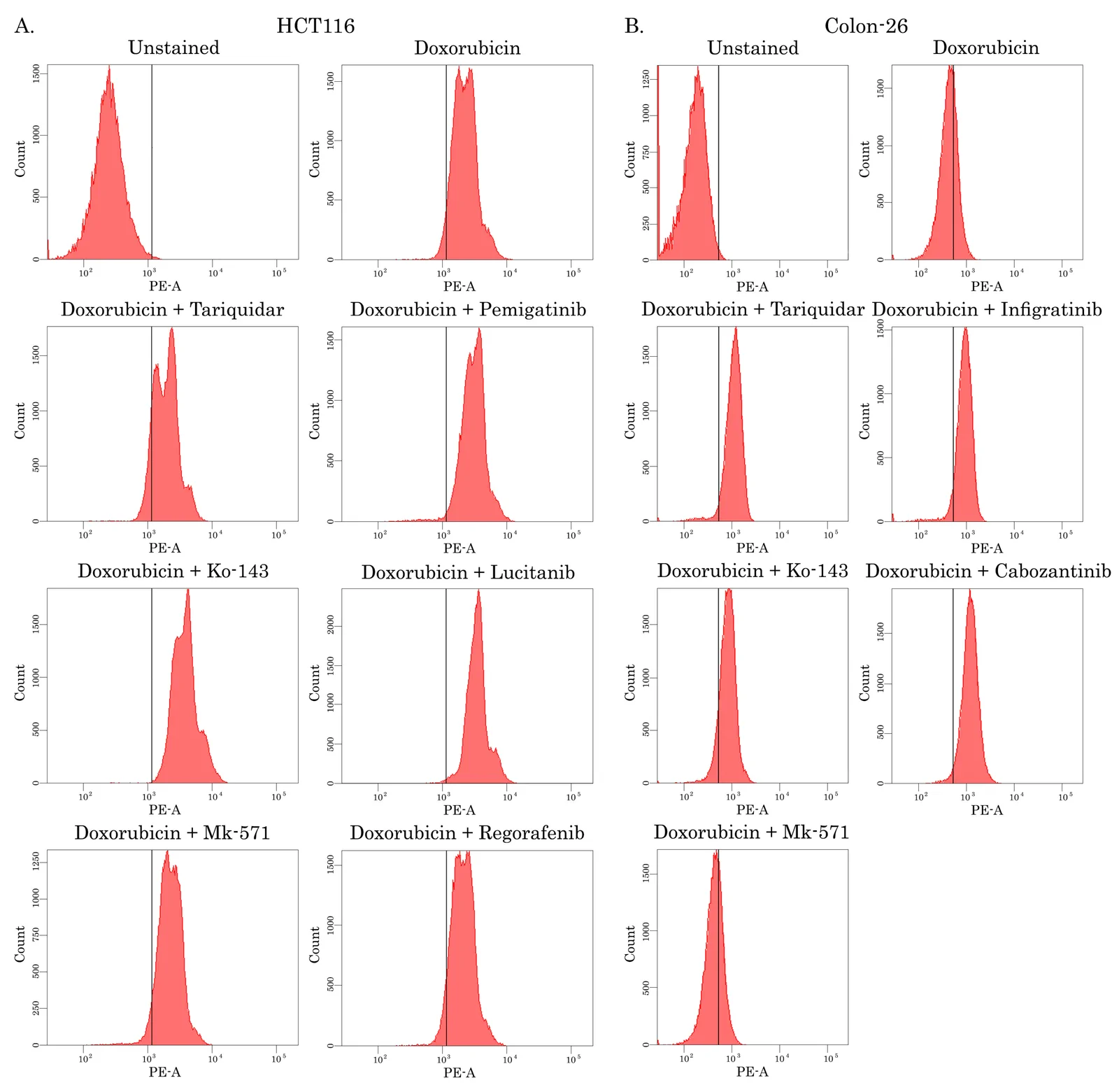
RTKIs increase the intracellular accumulation of Doxorubicin in HCT116 (**A**) and Colon-26 (**B**) cancer cell lines. The intracellular accumulation of Doxorubicin in combination with ABC transporter inhibitors (Tariquidar, Ko-143, and Mk-571) and RTKIs (Pemigatinib, Lucitanib, and Regorafenib for HCT116 cells and Infigratinib and Cabozantinib for Colon-26 cells). The cells were treated with 40 µM of Doxorubicin alone or with pre- and post-incubation with 20 µM of the aforementioned inhibitors. The vertical line in the figure illustrates the autofluorescence intensity in the unstained sample and was subsequently used to distinguish autofluorescence from doxorubicin-induced fluorescence. Representative histograms of at least 3 independent experiments are shown.

**Figure 13:**
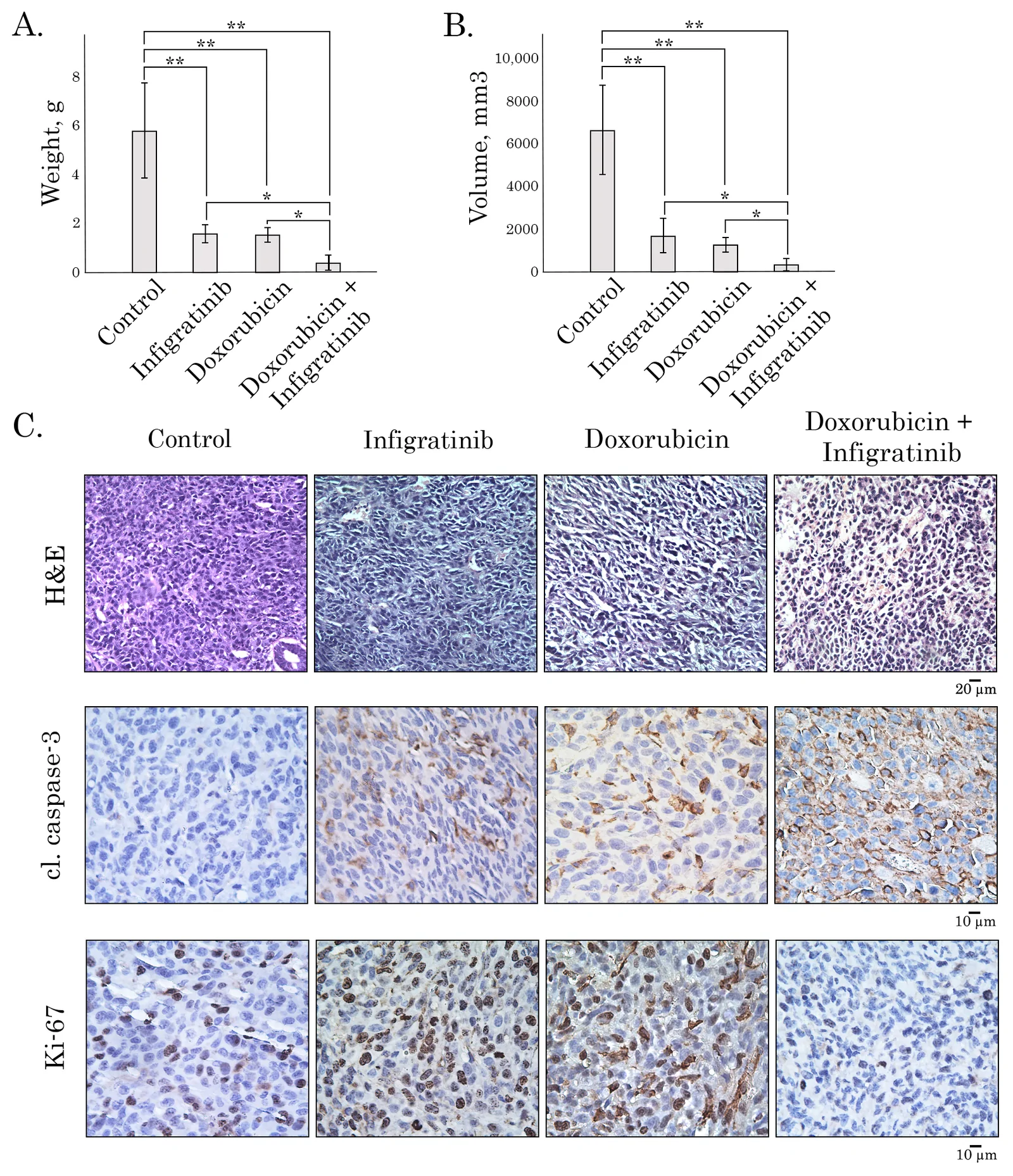
Antitumor effect of the combination (Infigratinib and Doxorubicin) on an allograft tumor model. After the subcutaneous inoculation of Colon-26 cells (day 14), Balb-c mice were randomized into four groups (n = 4). They administered i.p. 100 μL of vehicle (negative control), Infigratinib (10 mg/kg), Doxorubicin (10 mg/kg), or a combination 3 times a week for 3 weeks. Tumor volume (**A**) and tumor weight (**B**) of Colon-26 allografts at the end point of the experiment (3 weeks post-treatment) for each experimental group. (**C**) Changes in tumor tissue morphology in mice treated with the combination of Infigratinib and Doxorubicin. Hematoxylin and eosin staining (H&E) (20×) (upper panel) and immunohistochemical detection (40×) of the cleaved caspase-3 apoptotic marker (middle panel) and the Ki-67 proliferative marker (bottom panel) in Colon-26 allografts obtained from mice of the control group and mice treated with Infigratinib, Doxorubicin, and their combination. Significance level * *p* < 0.05, ** *p* < 0.01.

**Figure 14:**
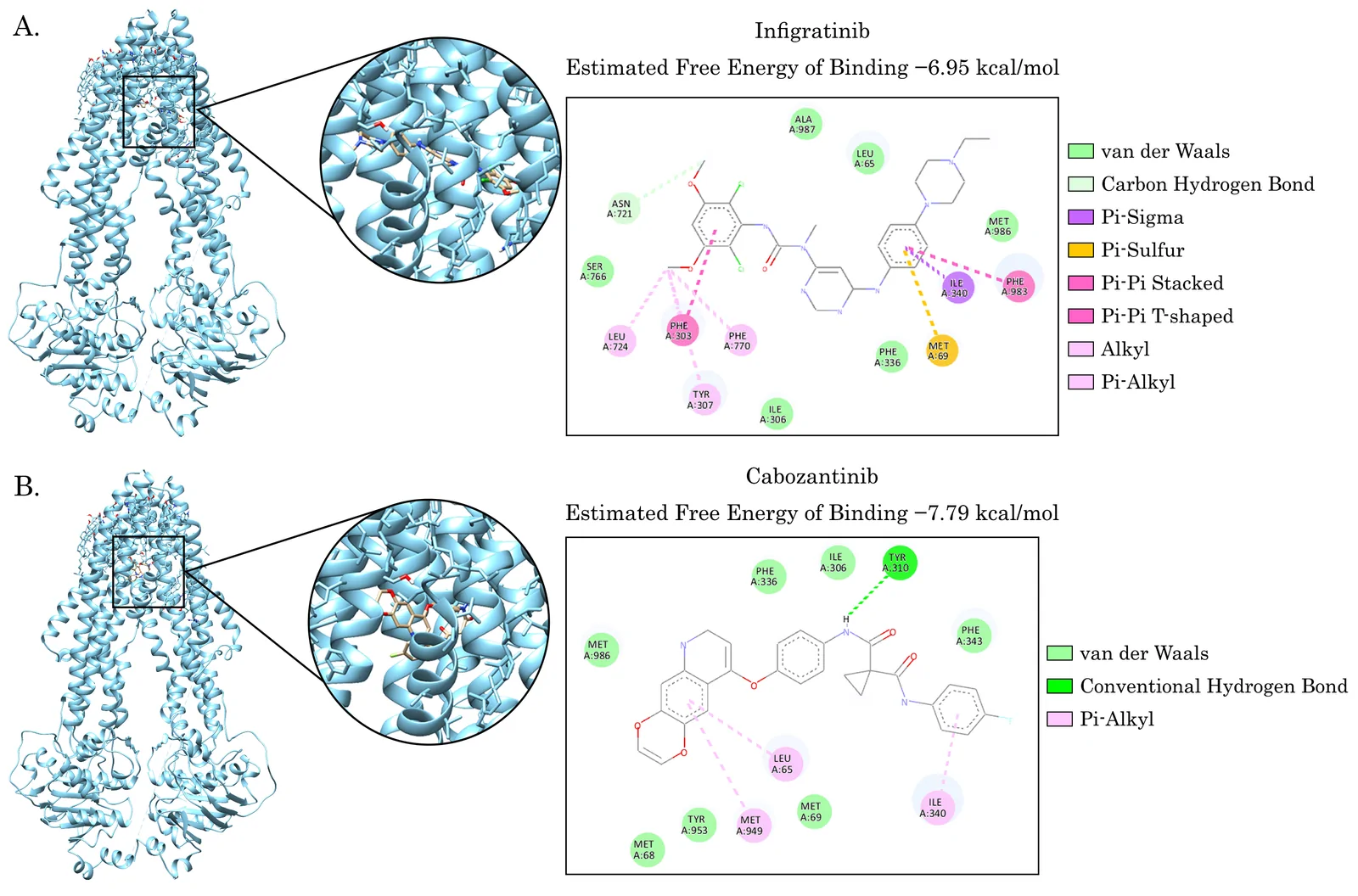
The three- and two-dimensional molecular interactions of Infigratinib (**A**) and Cabozantinib (**B**) with the ABCB1 transporter.

**Figure 15:**
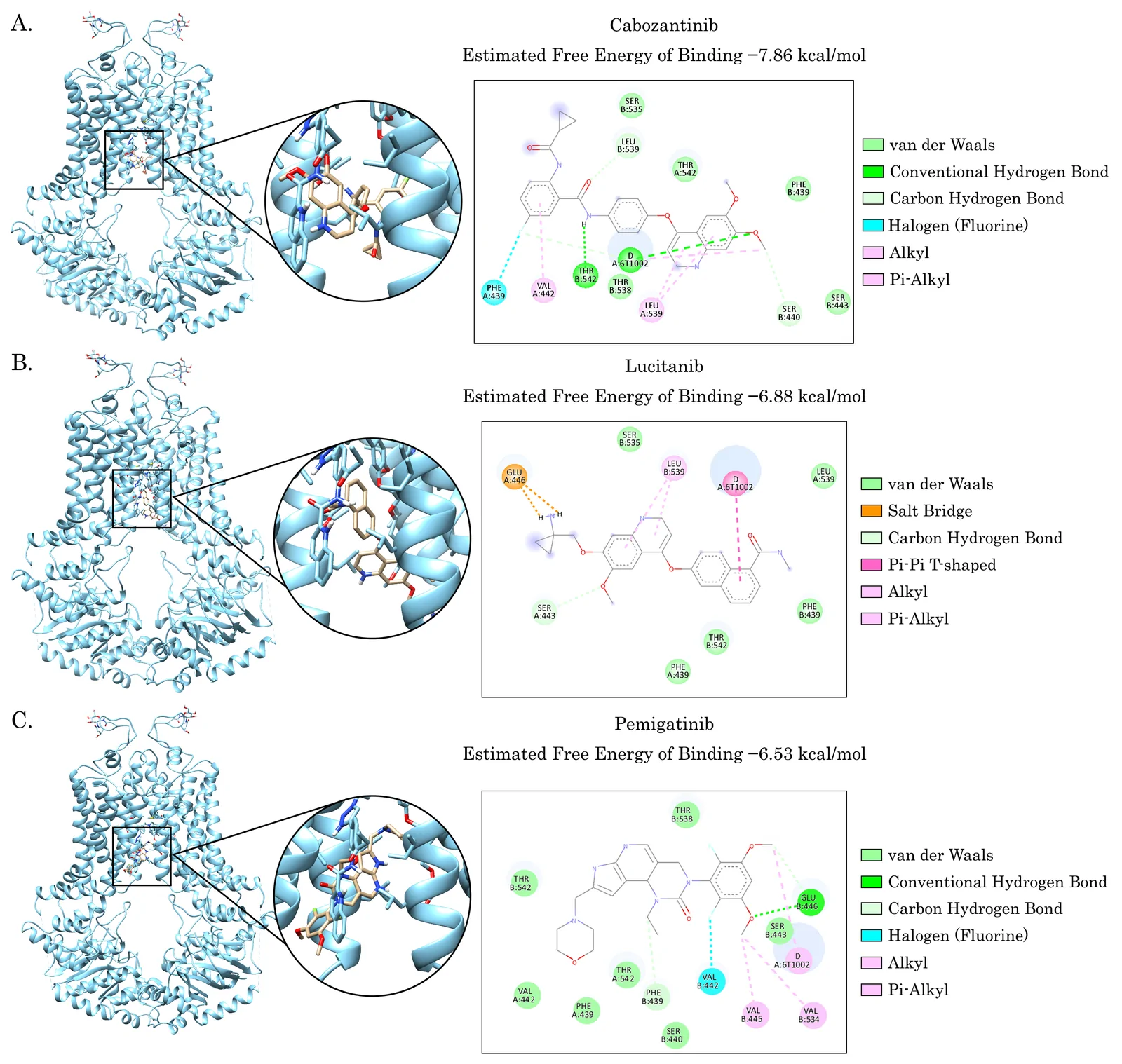
The three- and two-dimensional molecular interactions of Cabozantinib (**A**), Lucitanib (**B**), and Pemigatinib (**C**) with the ABCG2 transporter.

**Table 1:** Combined therapy with 10 µM RTKI decreases the IC_50_ values for Doxorubicin and 5-Fluorouracil in CRC lines. IC_50_ (µM) for Doxorubicin, 5-Fluorouracil, and receptor tyrosine kinase inhibitors alone and in combination.

	Colon-26			HCT116			RKO		
	—	Doxorubicin	5-Fluorouracil	—	Doxorubicin	5-Fluorouracil	—	Doxorubicin	5-Fluorouracil
—	—	9.83 ± 1.04	0.70 ± 0.15	—	1.14 ± 0.14	3.33 ± 0.24	—	<0.5	3.78 ± 0.38
Selective inhibitors of the FGFR signaling pathway									
Infigratinib	>100	<0.5	<0.5	12.15 ± 0.41	<0.5	1.47 ± 0.24	5.99 ± 0.23	<0.5	<0.5
Pemigatinib	>100	<0.5	<0.5	35.58 ± 5.64	<0.5	<0.5	>100	<0.5	<0.5
Alofanib	>100	24.34 ± 2.57	<0.5	>100	2.16 ± 0.62	2.47 ± 0.17	>100	<0.5	4.65 ± 0.72
Non-selective RTKI targeting FGFR and/or VEGFR signaling pathways									
Lucitanib	25.98 ± 0.88	4.96 ± 0.63	<0.5	17.46 ± 2.79	<0.5	3.61 ± 0.33	5.05 ± 0.51	<0.5	<0.5
Axitinib	>100	14.28 ± 1.73	0.84 ± 0.03	>100	<0.5	2.06 ± 0.35	>100	<0.5	3.43 ± 0.73
Cabozantinib	>100	1.58 ± 0.16	0.91 ± 0.11	>100	0.78 ± 0.43	1.86 ± 0.27	15.21 ± 1.14	<0.5	<0.5
Regorafenib	>100	9.08 ± 0.85	<0.5	27.95 ± 4.90	<0.5	14.46 ± 1.60	9.74 ± 1.88	<0.5	1.79 ± 0.68
Sunitinib	15.63 ± 1.26	2.89 ± 0.09	<0.5	5.36 ± 1.11	<0.5	3.58 ± 0.55	8.39 ± 0.57	<0.5	9.51 ± 1.16

“—”: untreated control for each cell line

**Table 2:** CRCs’ viability (%) treated with Doxorubicin, 5-Fluorouracil, or 10 µM of RTKI alone or in combination (72 h).

	Colon-26			HCT116			RKO		
	—	Doxorubicin 1 µM	5-Fluorouracil 1 µM	—	Doxorubicin 2 µM	5-Fluorouracil 4 µM	—	Doxorubicin 1 µM	5-Fluorouracil 2 µM
—	100.0 ± 5.1	100.5 ± 5.9	51.4 ± 9.0	100.0 ± 3.6	87.3 ± 3.3	85.5 ± 1.2	100.0 ± 6.7	61.7 ± 1.3	97.3 ± 1.7
**Selective inhibitors of the FGFR signaling pathway**									
Infigratinib	79.4 ± 4.3	52.0 ± 3.2	30.1 ± 2.6	85.5 ± 2.8	54.6 ± 1.8	85.7 ± 0.5	85.8 ± 1.5	48.4 ± 0.5	78.9 ± 1.8
Pemigatinib	52.0 ± 3.2	41.7 ± 1.7	24.7 ± 2.7	95.5 ± 2.8	40.8 ± 1.3	65.9 ± 1.2	91.6 ± 2.3	57.5 ± 2.4	57.1 ± 0.3
Alofanib	40.1 ± 2.6	51.8 ± 1.8	40.0 ± 0.2	85.5 ± 2.8	54.6 ± 1.8	81.2 ± 1.2	88.7 ± 0.4	37.5 ± 1.8	77.3 ± 8.4
**Non-selective RTKI targeting FGFR and/or VEGFR signaling pathways**									
Lucitanib	93.5 ± 0.5	58.2 ± 2.5	31.8 ± 3.4	81.0 ± 1.8	22.1 ± 0.7	91.3 ± 4.8	43.6 ± 1.4	46.6 ± 1.5	33.7 ± 2.1
Axitinib	87.2 ± 0.1	65.0 ± 0.1	39.9 ± 0.5	91.0 ± 1.2	56.5 ± 1.3	85.1 ± 1.5	98.2 ± 0.9	51.1 ± 0.7	61.2 ± 1.4
Cabozantinib	100.1 ± 8.7	30.5 ± 1.5	43.9 ± 1.3	105.1 ± 5.0	73.1 ± 3.6	79.3 ± 1.0	87.7 ± 1.8	45.5 ± 2.9	54.3 ± 1.1
Regorafenib	87.0 ± 8.6	49.6 ± 1.6	45.5 ± 1.1	89.8 ± 2.0	1.9 ± 0.01	74.6 ± 0.3	91.8 ± 0.8	49.1 ± 0.8	79.6 ± 3.2
Sunitinib	95.2 ± 1.2	63.9 ± 0.8	59.7 ± 8.2	82.2 ± 3.1	80.0 ± 0.2	61.8 ± 0.4	91.0 ± 1.5	63.7 ± 2.8	84.7 ± 3.3

“—”: untreated control for each cell line

**Table 3:** Synergy scores (HSA model) between the most potent RTKI and Doxorubicin or 5-Fluorouracil in colorectal cancer cell lines.

Colon-26		HCT116		RKO	
Doxorubicin + Infigratinib	17.16	Doxorubicin + Pemigatinib	10.03	Doxorubicin + Cabozantinib	10.38
Doxorubicin + Cabozantinib	16.5	Doxorubicin + Lucitanib	7.26	Doxorubicin + Infigratinib	2.57
Doxorubicin + Pemigatinib	15.08	Doxorubicin + Regorafenib	8.35	Doxorubicin + Alofanib	−0.73
5-Fluorouracil + Pemigatinib	1.42	5-Fluorouracil + Sunitinib	1.11	5-Fluorouracil + Lucitanib	7.76
5-Fluorouracil + Infigratinib	0.59	5-Fluorouracil + Pemigatinib	−1.05	5-Fluorouracil + Cabozantinib	7.21
5-Fluorouracil + Lucitanib	−0.35	5-Fluorouracil + Regorafenib	−2.56	5-Fluorouracil + Pemigatinib	−1.85

**Table 4:** The expression of MDR1, MRP-1, and ABCG2 mRNA in CRCs when compared with chemo-sensitive HCC1806 triple-negative breast cancer cell line. Values are averages of 3 experiments. Significance level ** *p* < 0.001, *** *p* < 0.0001.

Genes	*MDR1*	*MRP1*	*ABCG2*
HCC1806 TxR	26.50 ***	1.15	4.01 ***
HCT116	7.94 **	6.61 ***	4.82 ***
RKO	23.44 ***	22.25 ***	1.05
Colon-26	22.20 ***	115.30 ***	-

**Table 5:** The expression of MDR1, MRP-1, and ABCG2 mRNA in CRCs when compared with chemo-sensitive SaOS-2 osteosarcoma cancer cell line. Values are averages of 3 experiments. Significance level * *p* < 0.01, *** *p* < 0.0001.

Genes	*MDR1*	*MRP1*	*ABCG2*
SaOS-2 DoxR	2.88 *	1.36	0.96
HCT116	18.57 *	22.22 ***	15.73 ***
RKO	54.79 ***	74.83 ***	3.43 *
Colon-26	51.88 ***	387.89 ***	-

**Table 6:** The mean fluorescence intensity (MFI) of Doxorubicin in HCT116 and Colon-26 cells was determined from 3 independent experiments. The intracellular accumulation of Doxorubicin alone and with pre- and post-incubation with ABC transporter inhibitors (Tariquidar, Ko-143, and MK-571) and RTKIs (Pemigatinib, Lucitanib, and Regorafenib for HCT116, and Infigratinib and Cabozantinib for Colon-26). Significance level * *p* < 0.05, ** *p* < 0.01.

HCT116		Colon-26	
Doxorubicin	2407 ± 163	Doxorubicin	432 ± 46
Doxorubicin + Tariquidar	4129 ± 345 **	Doxorubicin + Tariquidar	1069 ± 158 **
Doxorubicin + Ko-143	3068 ± 210 *	Doxorubicin + Ko-143	822 ± 103 **
Doxorubicin + Mk-571	2329 ± 116	Doxorubicin + MK-571	446 ± 52
Doxorubicin + Pemigatinib	3187 ± 370 *	Doxorubicin + Infigratinib	891 ± 94 **
Doxorubicin + Lucitanib	3677 ± 494 **	Doxorubicin + Cabozantinib	1211 ± 125 **
Doxorubicin + Regorafenib	2188 ± 204		

**Table 7:** The competition assay was used to assess synergy between Infigratinib and Tariquidar or Ko-143, based on the enhancement of fluorescence intensity of Doxorubicin and Mitoxantrone. The mean fluorescence intensity (MFI) of Doxorubicin and Mitoxantrone in Colon-26 cells was determined from 3 independent experiments. Significance level ** *p* < 0.01.

Colon-26			
Doxorubicin	419 ± 26	Mitoxantrone	8811 ± 532
Doxorubicin + Infigratinib	881 ± 32	Mitoxantrone + Infigratinib	15,827 ± 956
Doxorubicin + Tariquidar	934 ± 51	Mitoxantrone + Tariquidar	15,067 ± 917
Doxorubicin + Tariquidar + Infigratinib	883 ± 39	Mitoxantrone + Tariquidar + Infigratinib	16,300 ± 1038
Doxorubicin + Ko-143	471 ± 22	Mitoxantrone + Ko-143	10,668 ± 741
Doxorubicin + Ko-143 + Infigratinib	907 ± 35 **	Mitoxantrone + Ko-143 + Infigratinib	14,130 ± 907 **

**Table 8:** Estimated Free Energy of Binding of the specific ABC transporter inhibitors and RTKI with ABCB1 and ABCG2.

	ABCB1	ABCG2
**Selective ABC transporter inhibitors**		
Tariquidar	−8.34 kcal/mol	−7.60 kcal/mol
Ko-143	−7.50 kcal/mol	−6.52 kcal/mol
**Selective inhibitors of the FGFR signaling pathway**		
Infigratinib	−6.95 kcal/mol	−6.35 kcal/mol
Pemigatinib	−6.24 kcal/mol	−6.53 kcal/mol
Alofanib	−6.30 kcal/mol	−6.18 kcal/mol
**Non-selective RTKI targeting FGFR and/or VEGFR signaling pathways**		
Lucitanib	−6.28 kcal/mol	−6.88 kcal/mol
Axitinib	−6.45 kcal/mol	−6.41 kcal/mol
Cabozantinib	−7.86 kcal/mol	−7.29 kcal/mol
Regorafenib	−6.83 kcal/mol	−6.15 kcal/mol
Sunitinib	−5.84 kcal/mol	−6.17 kcal/mol

**Table 9:** Important drug-binding cavity residues for RTKI interactions with ABCB1 and ABCG2. Amino acids involved in interaction with 3 or more inhibitors are underlined.

	ABCB1	ABCG2
**Selective ABC transporter inhibitors**		
Tariquidar	MET69, ILE306, TYR307, TYR310, PHE336, ILE340, PHE343, PHE728, PHE983	Chain A: PHE439, SER440, VAL442, SER443, GLU446, SER535, LEU539 Chain B: PHE439
Ko-143	PHE303, ALA336, ASN721, LEU724, PHE732, PHE770, SER979, ALA987	Chain A: PHE439, VAL442, SER443, THR538, LEU539, THR542 Chain B: PHE439, SER440, VAL442, SER443, THR538, LEU539, THR542
**Selective inhibitors of the FGFR signaling pathway**		
Infigratinib	LEU65, MET69, PHE303, ILE306, TYR307, PHE336, ILE340, ASN721, LEU724, SER766, PHE770, PHE983, MET986, ALA987	Chain A: PHE439, VAL442, SER443, GLU446 Chain B: PHE439, SER440, VAL442, SER443
Pemigatinib	PHE239, PHE770, GLN773, PHE777, LYS826, SER831, GLN838, VAL991, PHE994	Chain A: PHE439, VAL442, THR542 Chain B: PHE439, SER440, VAL442, SER443, VAL445, GLU446, VAL534, THR538, THR542
Alofanib	HIS61, LEU65, MET68, MET69, VAL125, ILE340, GLU875, PHE942, GLN946, MET949, TYR950, TYR953, MET986	Chain A: PHE439, LEU539, THR538, THR542 Chain B: PHE439, THR538, LEU539, THR542
**Non-selective RTKI targeting FGFR and/or VEGFR signaling pathways**		
Lucitanib	TRP232, LEU236, PHE239, ILE299, ALA302, PHE303, ILE306, PHE343, GLN990, PHE994	Chain A: PHE439, SER535, VAL536, LEU539, THR542 Chain B: PHE439, VAL442, SER443, GLU446
Axitinib	LEU65, PHE336, ILE340, PHE343, PHE732, TYR953, PHE983	Chain A: SER440, VAL442, THR538, LEU539, THR542 Chain B: PHE439, VAL442, THR542
Cabozantinib	LEU65, TYR310, PHE336, ILE340, GLN725, PHE728, PHE732, GLN946, MET949, TER950, TYR953, PHE978, SER979, PHE983, MET986	Chain A: PHE439, VAL442, LEU539, THR542 Chain B: PHE439, SER440, SER443, SER535, THR538, LEU539, THR542
Regorafenib	TRP232, LEU236, ILE299, ALA302, PHE303, ILE306, PHE770, GLN773, GLN838, VAL991, PHE994, ALA995	Chain A: PHE439, VAL442, SER443, GLU446, THR538, THR542 Chain B: PHE439, SER440, SER443
Sunitinib	PHE336, ASN721, GLN725, ASN839, ASN842, PHE983, ALA987, VAL991	Chain A: PHE439, SER440, VAL442, SER443, GLU446, THR542 Chain B: PHE439, THR542

## Data Availability

The original contributions presented in the study are included in the article and Supplementary Materials; further enquiries can be directed to the corresponding author.

## Supplementary Materials

The following supporting information can be downloaded at: https://www.mdpi.com/article/10.3390/cancers18193074/s1. **Figure S1**: MTS-based viability assay in HCT116 cells treated with RTKIs for 72 h. The data were normalized to DMSO-treated controls. **Figure S2**: Representative bright-field images of Colon-26 colon cancer cells after exposure to DMSO (control), Doxorubicin (1 µM), RTKIs (Infigratinib, Cabozantinib, and Pemigatinib—10 µM), and their combinations. **Figure S3**: Cristal violet staining of Colon-26 cancer cells treated with DMSO (control), Doxorubicin (1 µM), and RTKIs (i.e., Infigratinib, Cabozantinib, Pemigatinib—10 µM) alone, and when combined with Doxorubicin. **Figure S4**: Anti-proliferative effects of Infigratinib used alone or in combination with 5-Fluorouracil in the Colon-26 colon cancer cell line. **Figure S5**: An impact of Regorafenib alone or in combination with Doxorubicin on FGFR and VEGFR signaling pathways in the HCT116 cancer cell line. Changes in expression of total and phosphorylated forms of FGFR1/2 and VEGFR1 and downstream signaling molecules AKT and MAPK were assessed at 0.5, 2, 6, and 24 h post-treatment. Actin stain was used as a loading control. **Figure S6**: RTKI did not influence the subcellular localization of MDR1 (**A**), MRP-1 (**B**), and BCRP1 (**C**) in Colon-26 cells. **Figure S7**: RTKIs increase the intracellular accumulation of Mitoxantrone in the HCT116 cancer cell line. The intracellular accumulation of Mitoxantrone in combination with ABC transporter inhibitors (Tariquidar, Ko-143, and Mk-571) and RTKIs (Pemigatinib, Lucitanib, Regorafenib). **Figure S8**: RTKIs increase the intracellular accumulation of Calcein AM, a specific MDR1 substrate, in the HCT116 cancer cell line. The intracellular accumulation of Calcein AM in combination with ABC transporter inhibitors (Tariquidar, Ko-143, and Mk-571) and RTKIs (Pemigatinib, Lucitanib, Regorafenib). **Figure S9**: Lack of pro-apoptotic activity in HCT116 cells treated with Doxorubicin or Mitoxantrone (40 µM) alone or in presence of Lucitanib and Regorafenib (20 µM) for 6 h. Cleaved poly-(ADP)-ribose-polymerase (PARP) and caspase-3 stain were used as apoptotic markers, whereas actin staining was used to show the comparable amounts of protein loaded into each sample. **Table S1**: Molecular targets and clinical application of the studied RTKI. **Table S2**: Synergy scores (ZIP model) between the most potent RTKI and Doxorubicin or 5-Fluorouracil in colorectal cancer cell lines. **Table S3**: The expression of MDR1, MRP-1, and ABCG2 mRNA in CRCs when compared with chemo-resistant HCC1806 TxR triple-negative breast cancer cell subline. **Table S4**: The expression of MDR1, MRP-1, and ABCG2 mRNA in CRCs when compared with chemo-resistant SaOS-2 DoxR osteosarcoma cancer cell subline. **Table S5**: The mean fluorescence intensity of Mitoxantrone in HCT116 cells was determined from 3 independent experiments. The intracellular accumulation of Mitoxantrone alone and with pre- and post-incubation with ABC transporter inhibitors (Tariquidar, Ko-143, and Mk-571) and RTKIs (Pemigatinib, Lucitanib, Regorafenib). **Table S6**: The mean fluorescence intensity of Calcein AM, a specific MDR1 substrate, in HCT116 cells was determined from 3 independent experiments. The intracellular accumulation of Calcein AM alone and in combination with ABC transporter inhibitors (Tariquidar, Ko-143, and Mk-571) and RTKIs (Pemigatinib, Lucitanib, Regorafenib) in HCT116. **Table S7**: FACS analysis of cell cycle profile of Colon-26 cells treated with RTKIs (10 μM) or Doxorubicin (1 μM) for 72 h. **File S1:** The uncropped original Western blotting images.

## Abbreviations

The following abbreviations are used in this manuscript:

ABC transporters	ATP-binding cassette transporters
MDR	Multidrug resistance
FGFR	Fibroblast growth factor receptor
VEGFR	Vascular endothelial growth factor receptor
CRC	Colorectal cancer
P-gp	P-glycoprotein
BCRP	Breast cancer resistance protein
MRP	Multidrug resistance-associated protein
RTKI	Receptor tyrosine kinase inhibitor
SC	Synergy score
IHC staining	Immunohistochemical staining
EMT	Epithelial-to-mesenchymal transition
CSC	Cancer stem cell
PARP	Poly-(ADP)-ribose-polymerase
FDA	Food and Drug Administration
ATCC	American Type Culture Collection
SaOS-2 DoxR	Doxorubicin-resistant SaOS-2 osteosarcoma subline
HCC1806 Tx-R	Paclitaxel-resistant HCC1806 triple-negative breast cancer subline
GIST T-1R	Imatinib-resistant gastrointestinal stromal tumor subline
DMSO	Dimethyl sulfoxide
SDS	Sodium Dodecyl Sulfate solution
PBS	Phosphate-buffered saline
RIPA buffer	Radio-immunoprecipitation buffer
PCR	Polymerase Chain Reaction
RNA	Ribonucleic acid
FFPE tissues	Formalin-fixed, paraffin-embedded tissues
H&E	Hematoxylin and Eosin
SE	Standard error
IC_50_	Half maximal inhibitory concentration
HSA model	Highest Single Agent model
MFI	Mean fluorescence intensity
TMD	Transmembrane domain
