# Supplementary material for: Re-Evaluating Multi-Kinase Inhibitors Targeting FGFR and VEGFR Pathways for Combined Therapy in Colorectal Cancer: A Preclinical Study
Source: Cancers (Basel). 2026 Sep 22;18(19):3074. doi: 10.3390/cancers18193074 (PMC13664539; doi:10.3390/cancers18193074)

Figure 2. Assessment of activation of FGFR and VEGFR signaling pathways in 3 CRC cell lines (Colon-26, HCT116, and RKO) by western blotting.

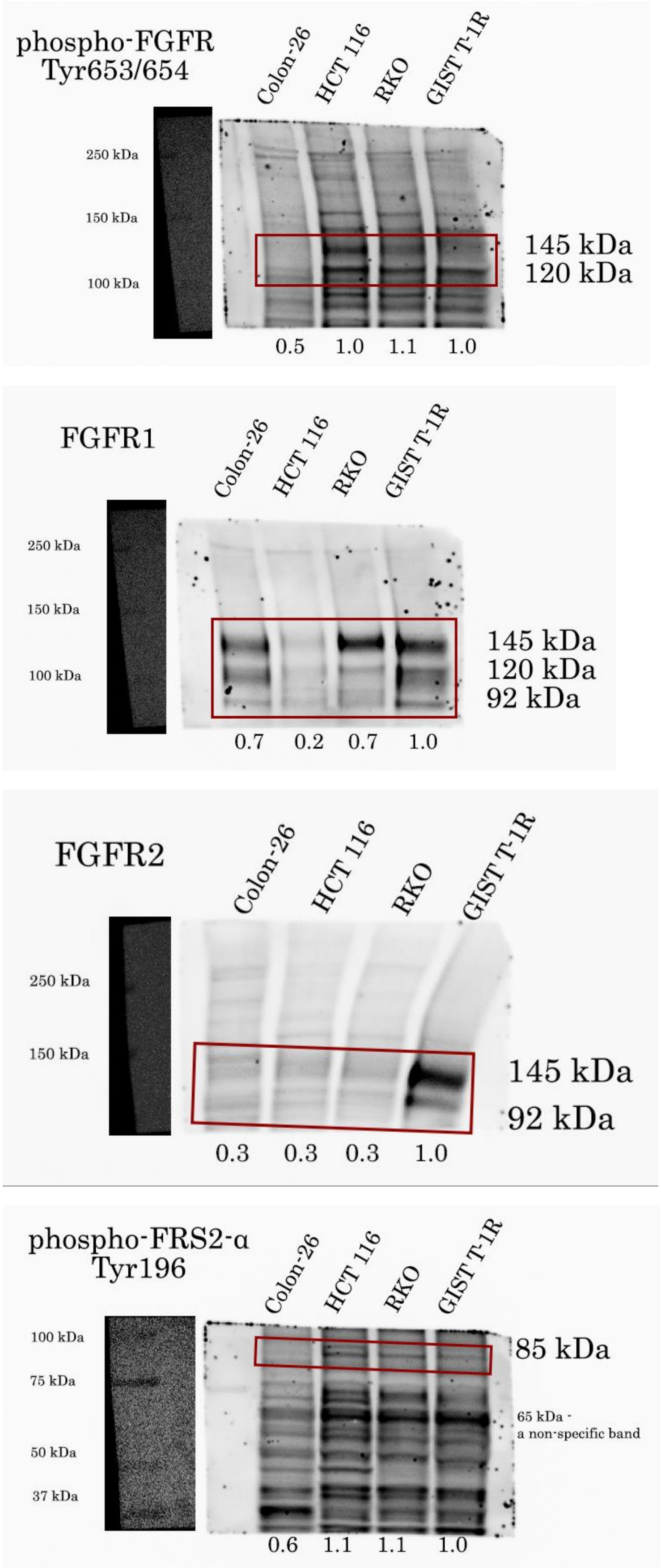

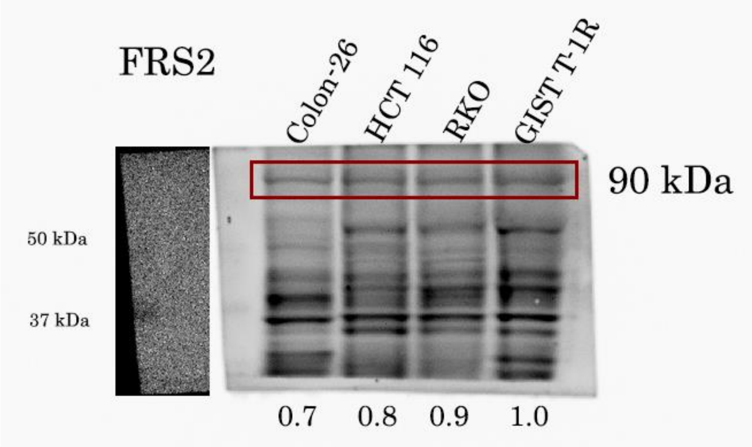
FRS2
Colon-26
HCT 116
RKO
GIST T-1R
90 kDa
50 kDa
37 kDa
0.7
0.8
0.9
1.0

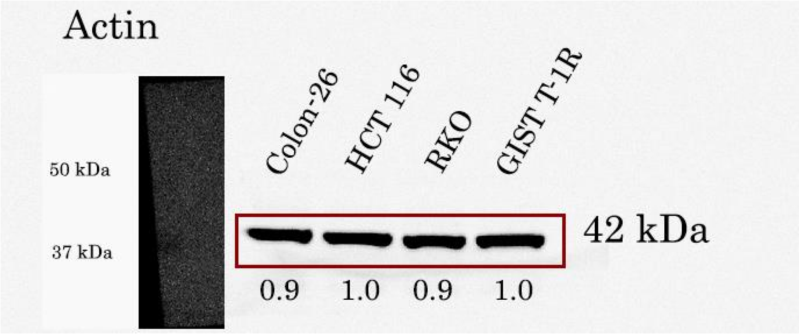
Actin
Colon-26
HCT 116
RKO
GIST T-1R
50 kDa
37 kDa
42 kDa
0.9
1.0
0.9
1.0

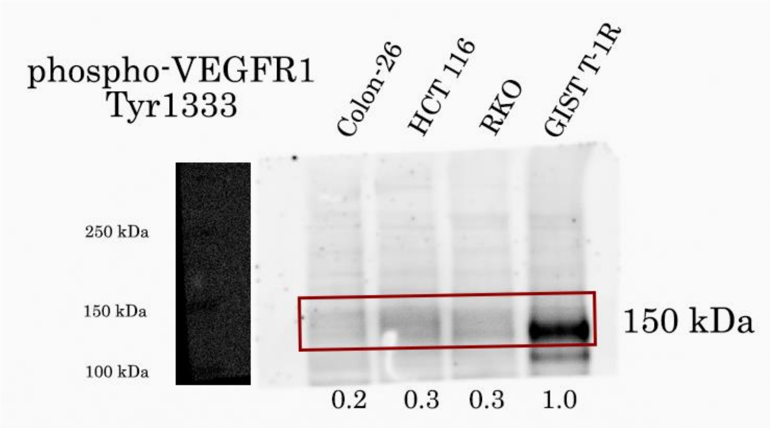
phospho-VEGFR1
Tyr1333
Colon-26
HCT 116
RKO
GIST T-1R
250 kDa
150 kDa
100 kDa
150 kDa
0.2
0.3
0.3
1.0

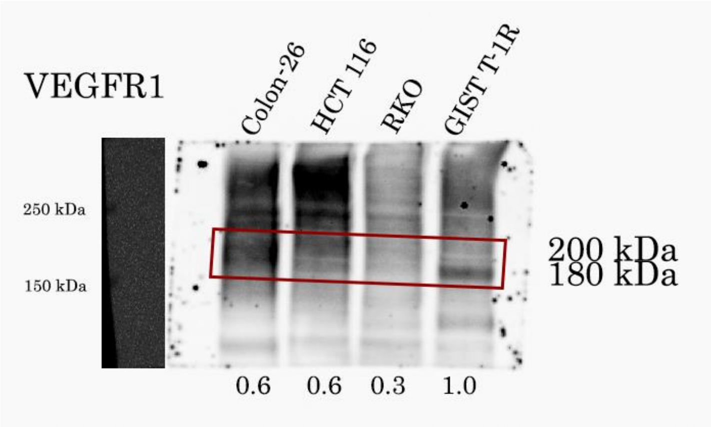
VEGFR1
Colon-26
HCT 116
RKO
GIST T-1R
250 kDa
150 kDa
200 kDa
180 kDa
0.6
0.6
0.3
1.0

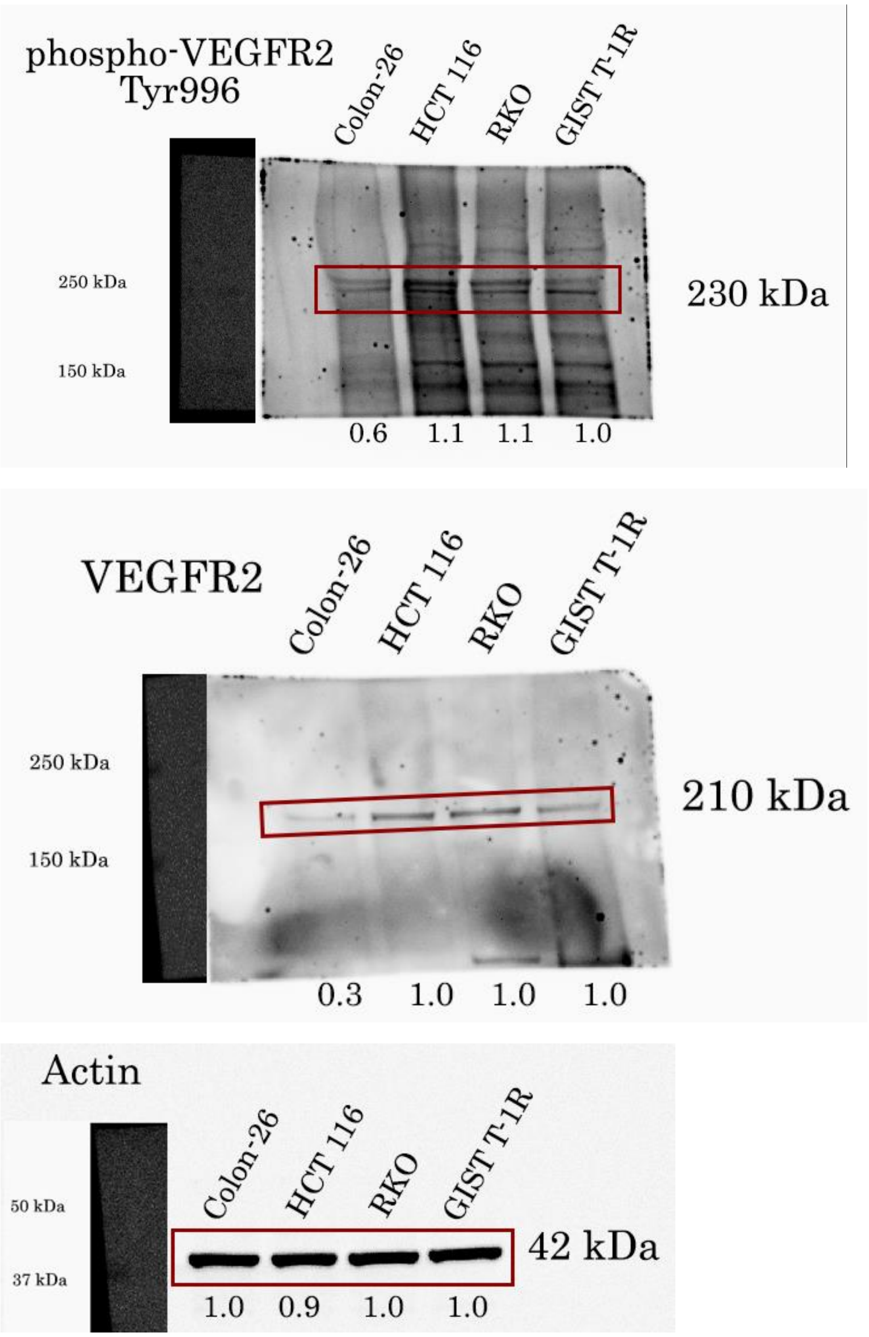

phospho-VEGFR2
Tyr996
Colon-26
HCT 116
RKO
GIST T-1R
250 kDa
150 kDa
230 kDa
0.6
1.1
1.1
1.0
VEGFR2
Colon-26
HCT 116
RKO
GIST T-1R
250 kDa
150 kDa
210 kDa
0.3
1.0
1.0
1.0
Actin
Colon-26
HCT 116
RKO
GIST T-1R
50 kDa
37 kDa
42 kDa
1.0
0.9
1.0
1.0

Figure 6. Pro-apoptotic activity of RTKIs, Doxorubicin alone, and in combination in CRC cell lines.

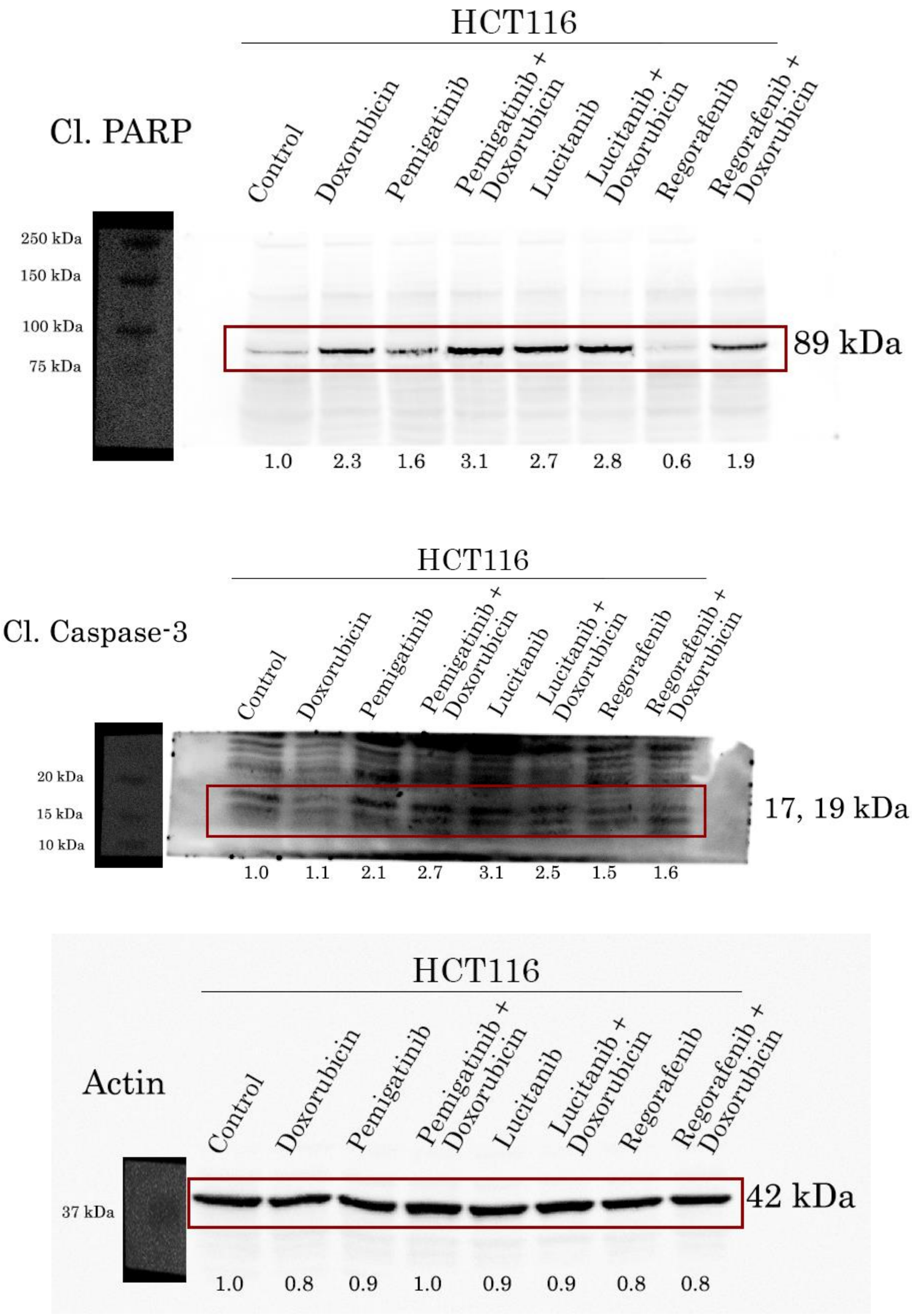

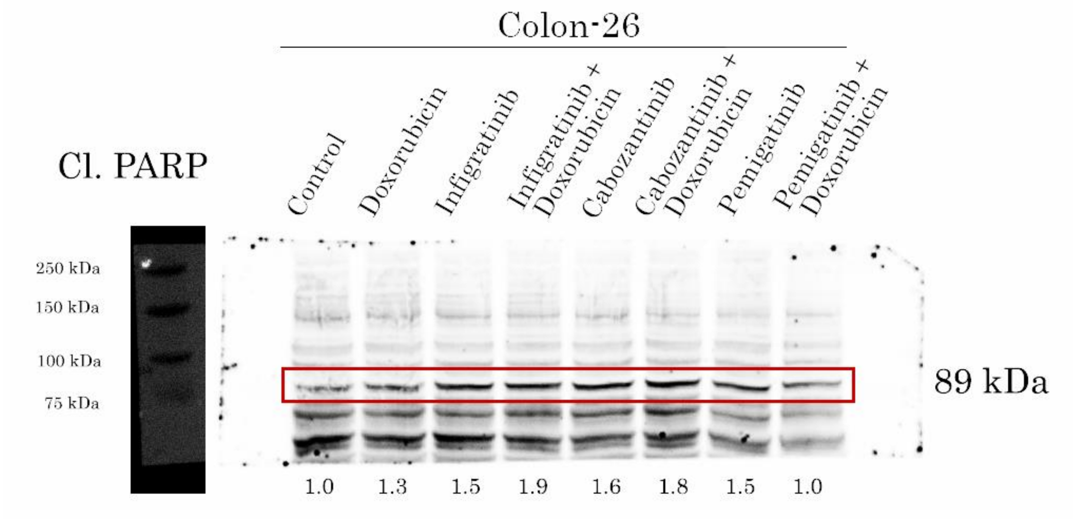
Colon-26
Cl. PARP
Control
Doxorubicin
Infigratinib
Infigratinib + Doxorubicin
Cabozantinib
Cabozantinib + Doxorubicin
Pemigatinib
Pemigatinib + Doxorubicin
250 kDa
150 kDa
100 kDa
75 kDa
89 kDa
1.0 1.3 1.5 1.9 1.6 1.8 1.5 1.0

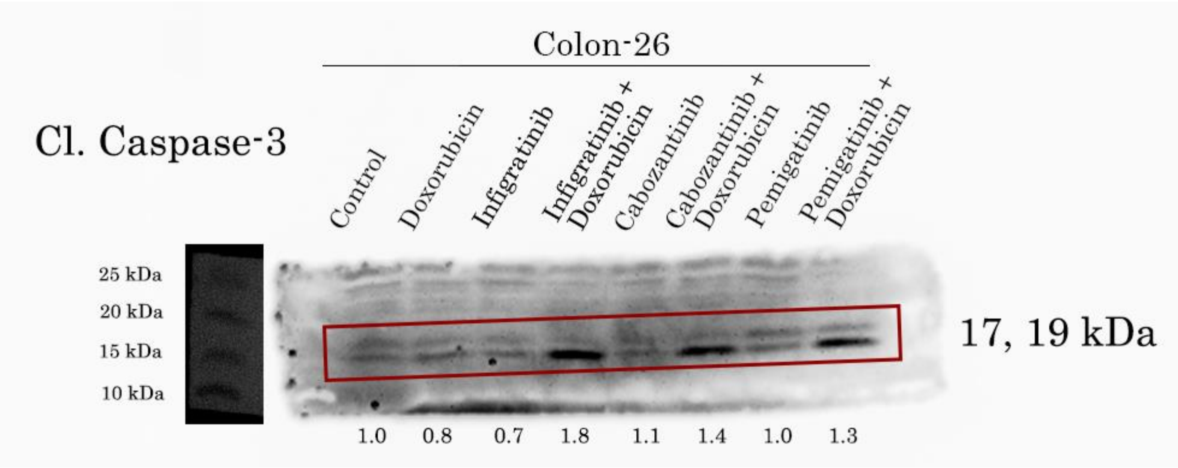
Colon-26
Cl. Caspase-3
Control
Doxorubicin
Infigratinib
Infigratinib + Doxorubicin
Cabozantinib
Cabozantinib + Doxorubicin
Pemigatinib
Pemigatinib + Doxorubicin
25 kDa
20 kDa
15 kDa
10 kDa
17, 19 kDa
1.0 0.8 0.7 1.8 1.1 1.4 1.0 1.3

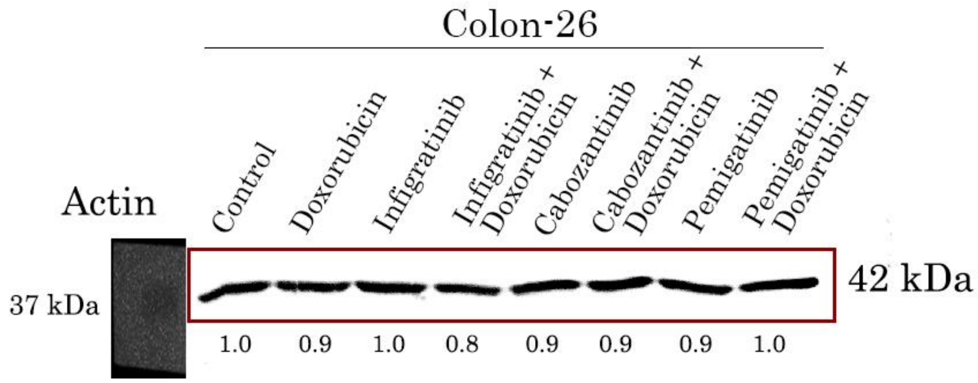
Colon-26
Actin
Control
Doxorubicin
Infigratinib
Infigratinib + Doxorubicin
Cabozantinib
Cabozantinib + Doxorubicin
Pemigatinib
Pemigatinib + Doxorubicin
37 kDa
42 kDa
1.0 0.9 1.0 0.8 0.9 0.9 0.9 1.0

Figure 8. An impact of RTKI (Pemigatinib, Lucitanib and Regorafenib) on FGFR and VEGFR signaling pathways in the HCT116 cancer cell line.

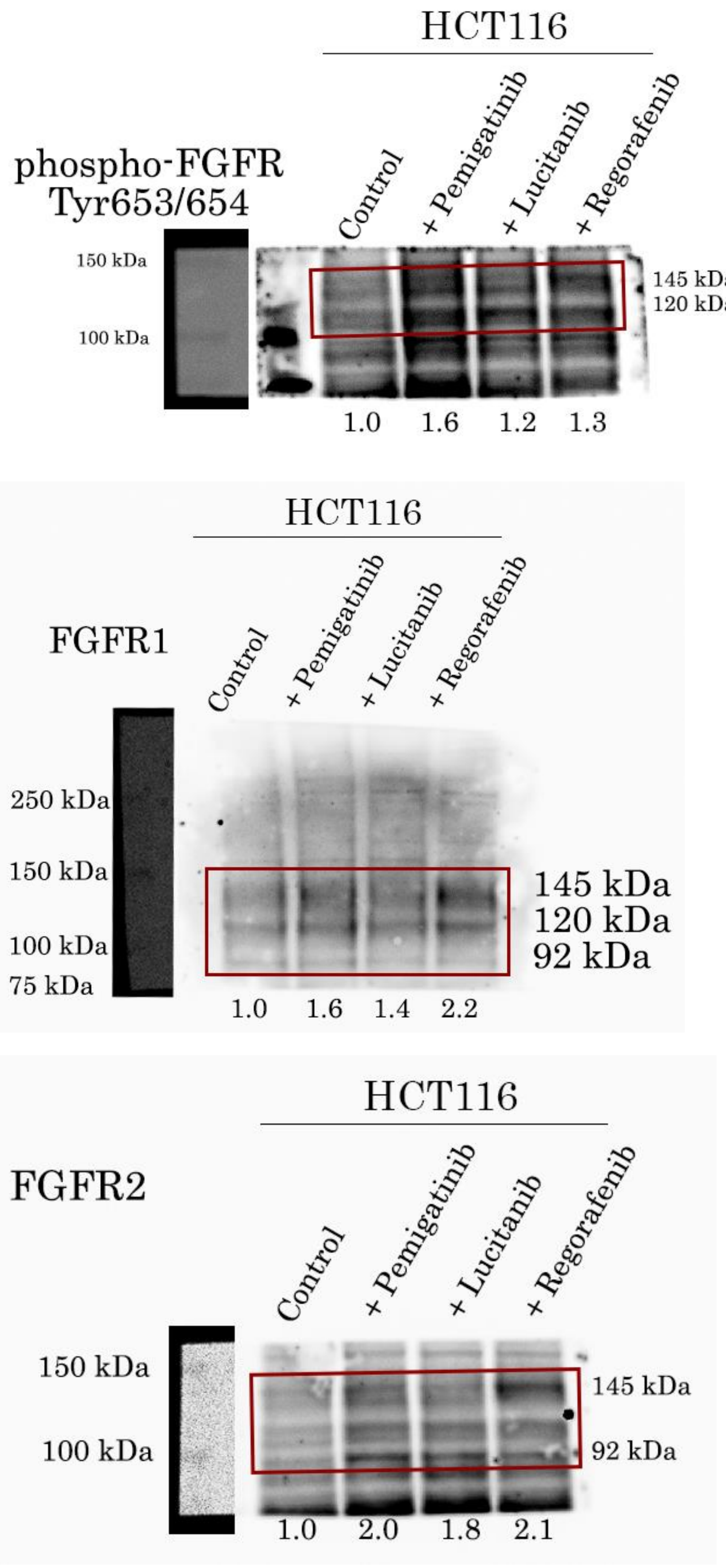

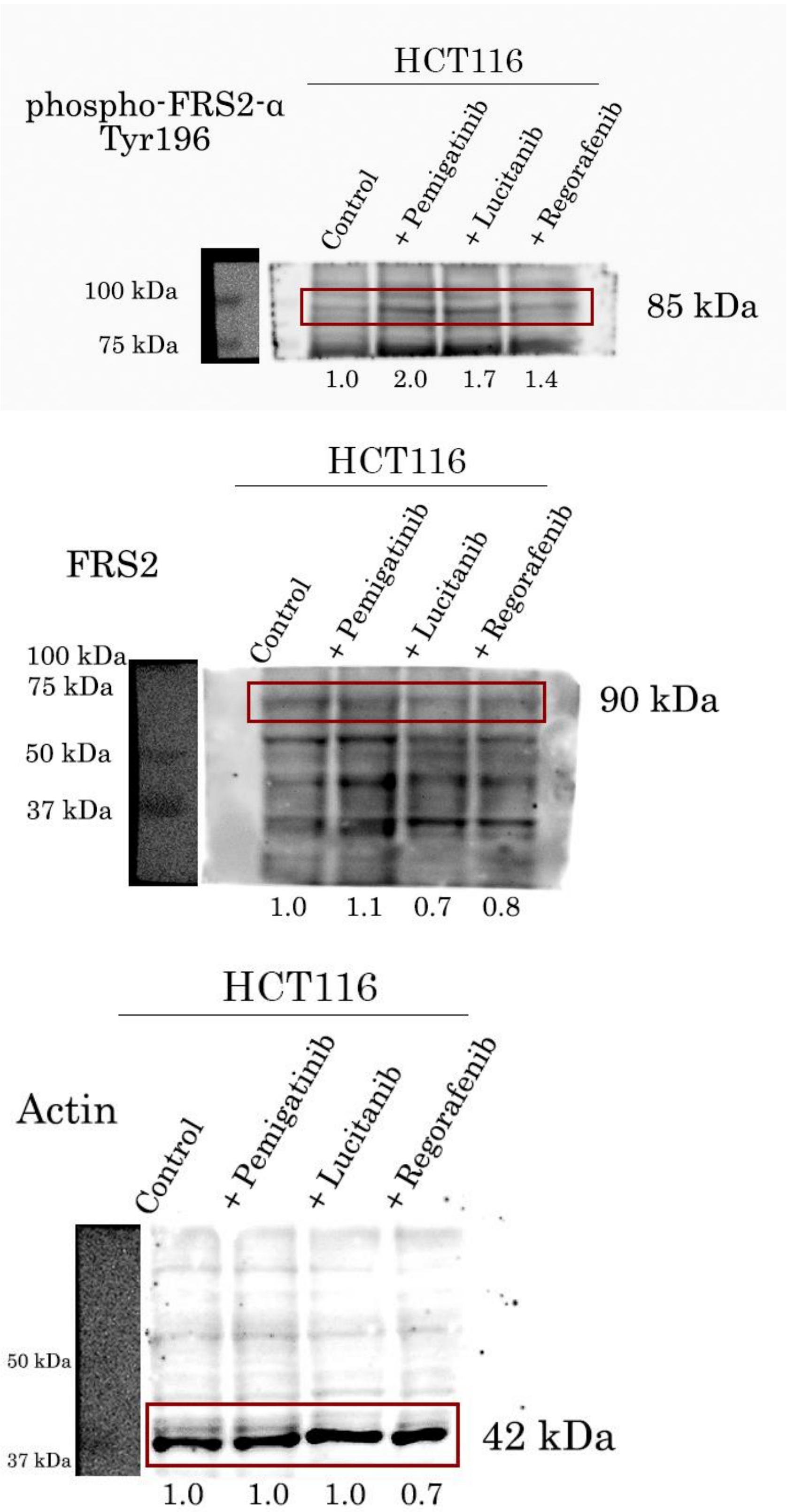

HCT116
phospho-FRS2-α Tyr196
Control
+ Pemigatinib
+ Lucitanib
+ Regorafenib
100 kDa
75 kDa
85 kDa
1.0 2.0 1.7 1.4
HCT116
FRS2
Control
+ Pemigatinib
+ Lucitanib
+ Regorafenib
100 kDa
75 kDa
50 kDa
37 kDa
90 kDa
1.0 1.1 0.7 0.8
HCT116
Actin
Control
+ Pemigatinib
+ Lucitanib
+ Regorafenib
50 kDa
37 kDa
42 kDa
1.0 1.0 1.0 0.7

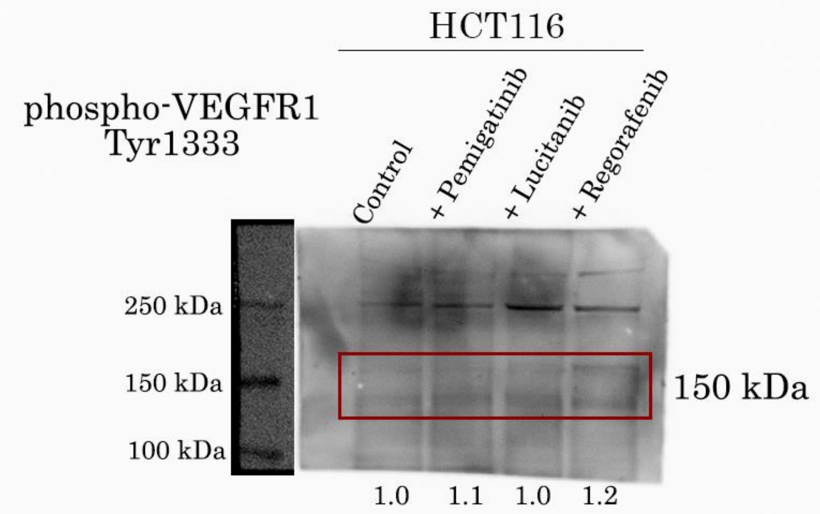
HCT116
phospho-VEGFR1
Tyr1333
Control
+ Pemigatinib
+ Lucitanib
+ Regorafenib
250 kDa
150 kDa
100 kDa
150 kDa
1.0
1.1
1.0
1.2

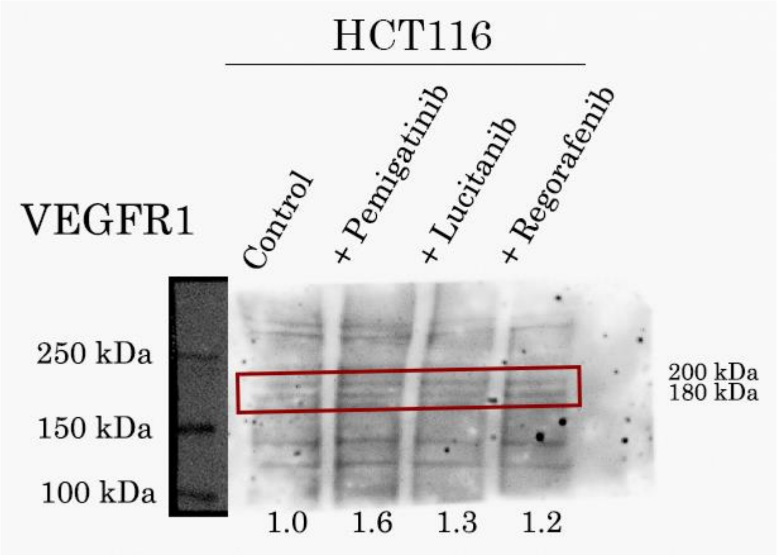
HCT116
VEGFR1
Control
+ Pemigatinib
+ Lucitanib
+ Regorafenib
250 kDa
150 kDa
100 kDa
200 kDa
180 kDa
1.0
1.6
1.3
1.2

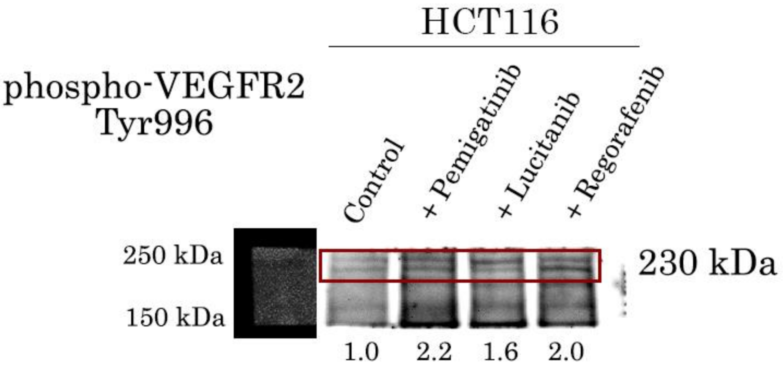
HCT116
phospho-VEGFR2
Tyr996
Control
+ Pemigatinib
+ Lucitanib
+ Regorafenib
250 kDa
150 kDa
230 kDa
1.0
2.2
1.6
2.0

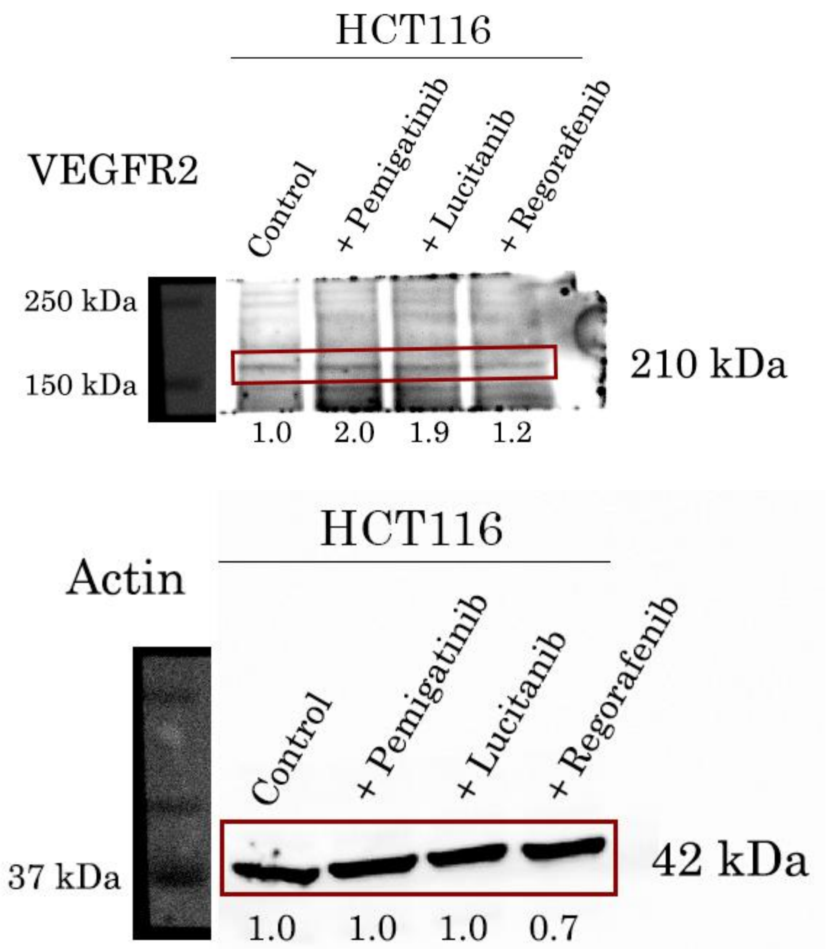
HCT116
VEGFR2
Control
+ Pemigatinib
+ Lucitanib
+ Regorafenib
250 kDa
150 kDa
210 kDa
1.0
2.0
1.9
1.2
HCT116
Actin
Control
+ Pemigatinib
+ Lucitanib
+ Regorafenib
37 kDa
42 kDa
1.0
1.0
1.0
0.7

Figure 9. The expression of ABC transporters (MDR1, MRP-1, and BCRP1) in Colon-26, HCT116, and RKO colorectal cancer cells.

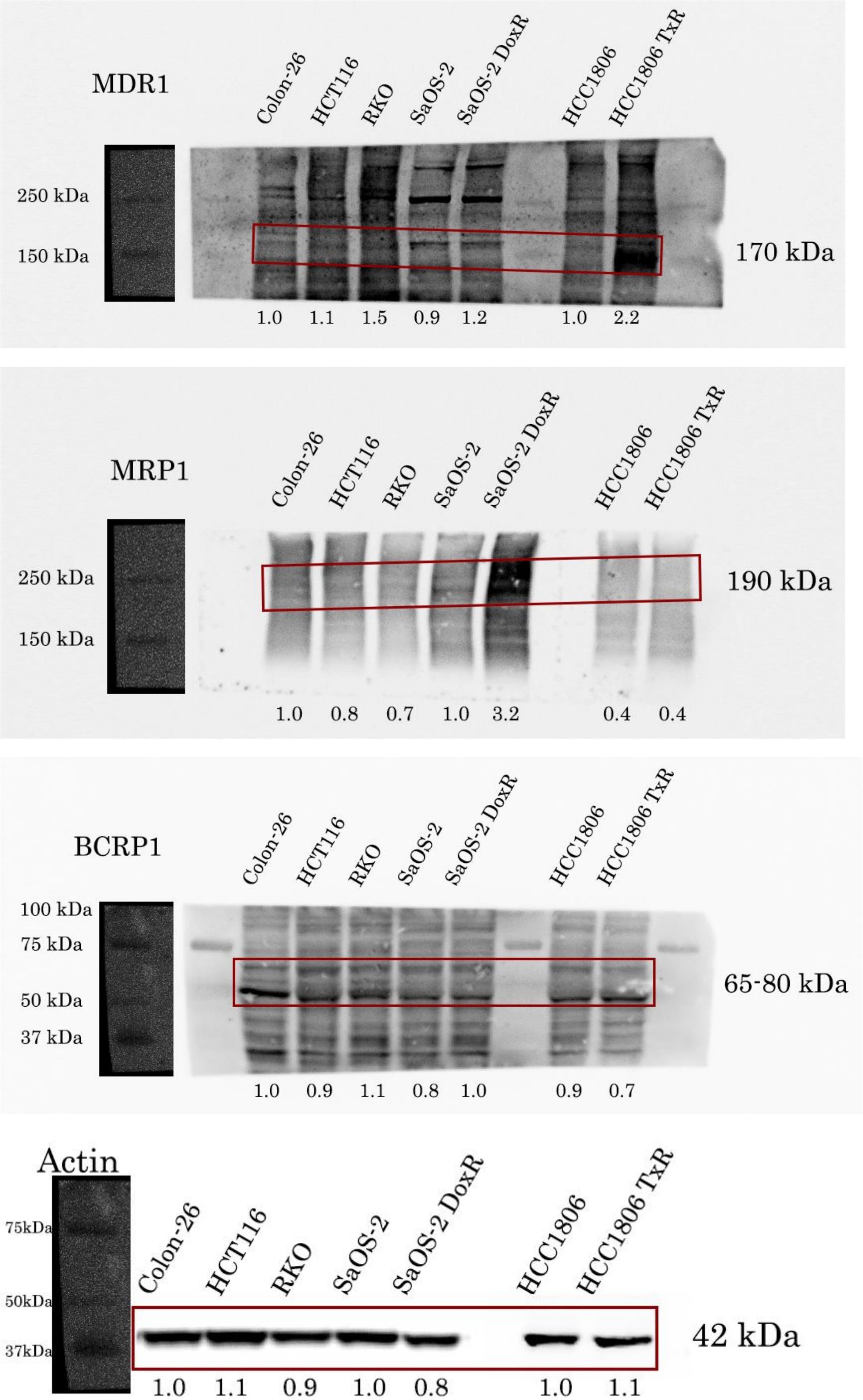

Figure 10. Expression of ABC transporters in HCT116 and Colon-26 colon cancer cells treated with RTKI.

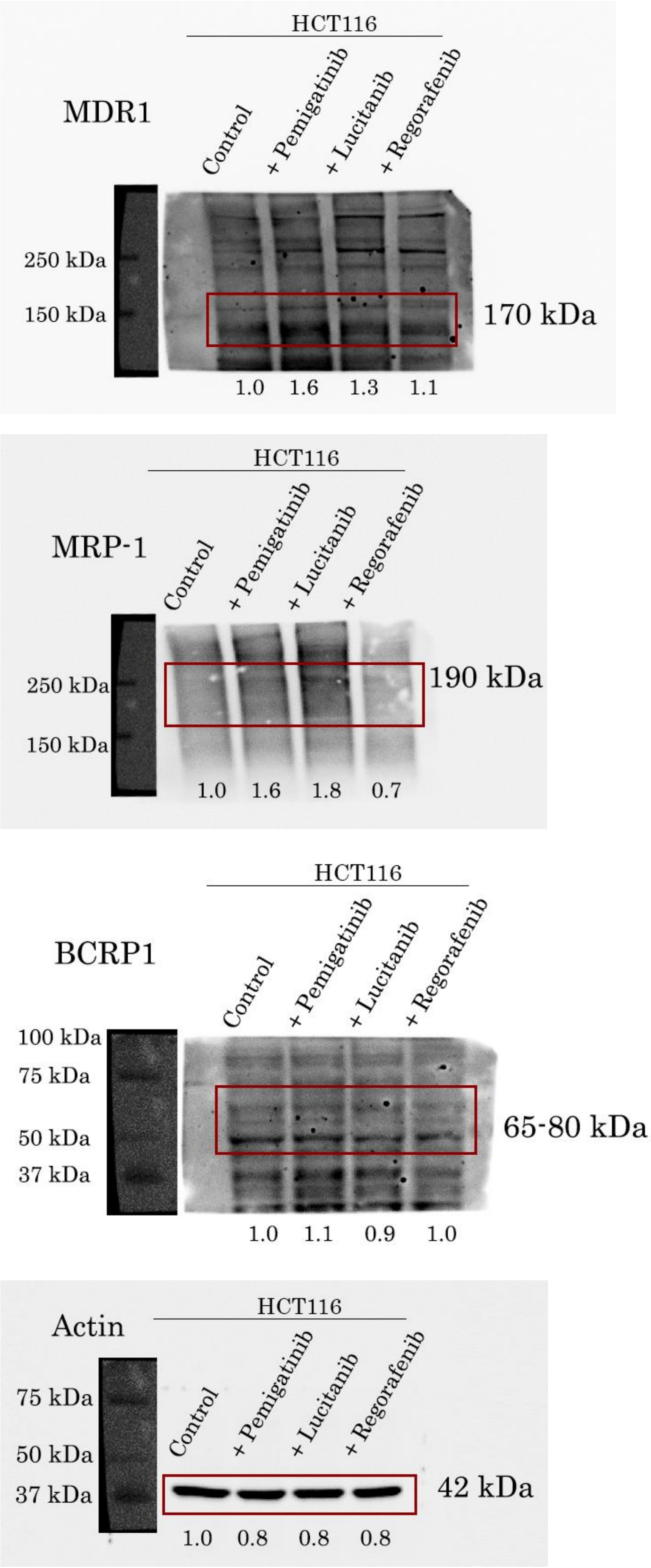

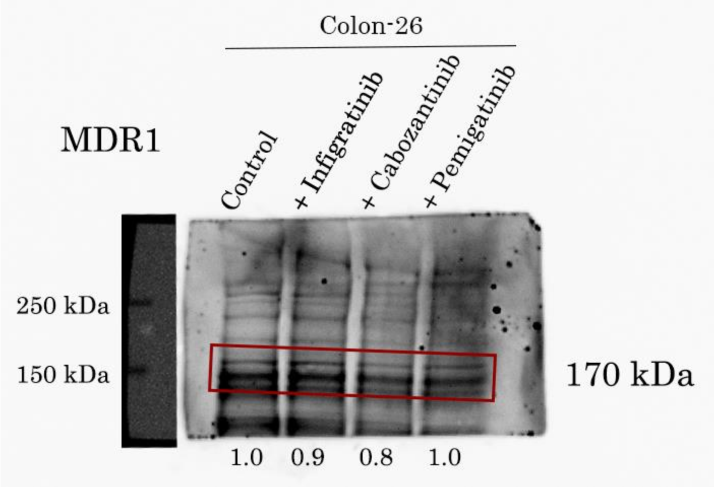
MDR1
Colon-26
Control
+ Infigratinib
+ Cabozantinib
+ Pemigatinib
250 kDa
150 kDa
170 kDa
1.0 0.9 0.8 1.0

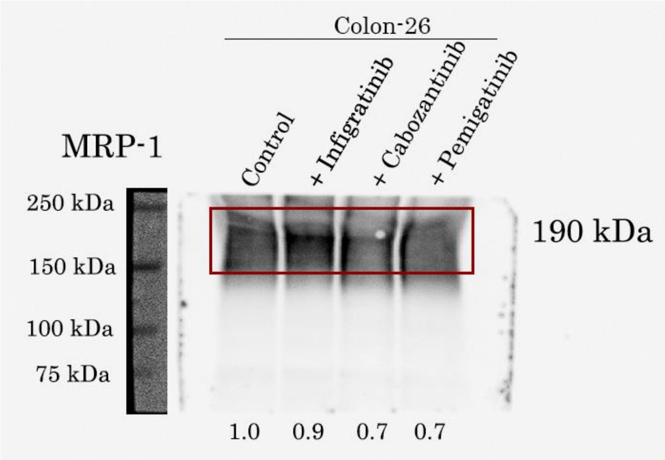
MRP-1
Colon-26
Control
+ Infigratinib
+ Cabozantinib
+ Pemigatinib
250 kDa
150 kDa
100 kDa
75 kDa
190 kDa
1.0 0.9 0.7 0.7

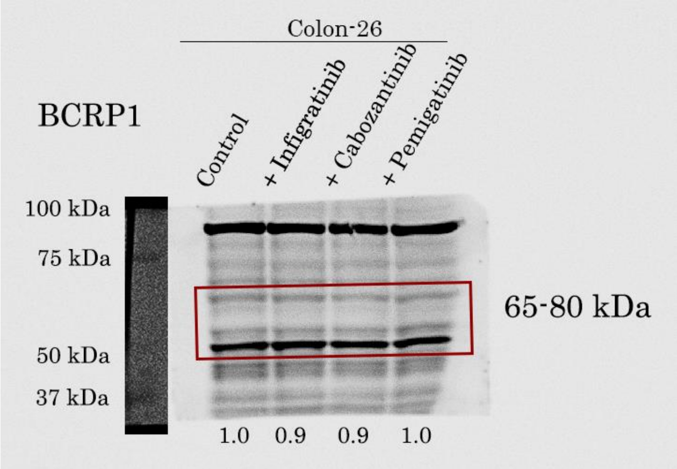
BCRP1
Colon-26
Control
+ Infigratinib
+ Cabozantinib
+ Pemigatinib
100 kDa
75 kDa
50 kDa
37 kDa
65-80 kDa
1.0 0.9 0.9 1.0

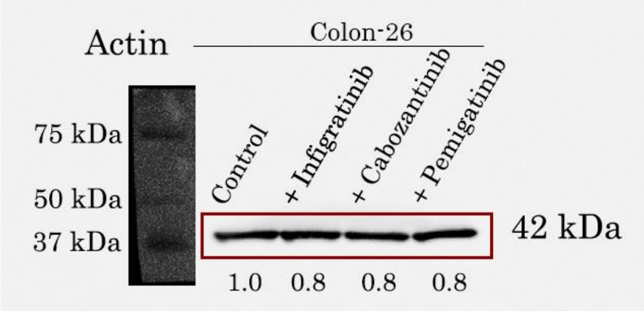
Actin
Colon-26
Control
+ Infigratinib
+ Cabozantinib
+ Pemigatinib
75 kDa
50 kDa
37 kDa
42 kDa
1.0 0.8 0.8 0.8

Figure S5. An impact of Regorafenib alone or in combination with Doxorubicin on FGFR and VEGFR signaling pathways in the HCT116 cancer cell line.

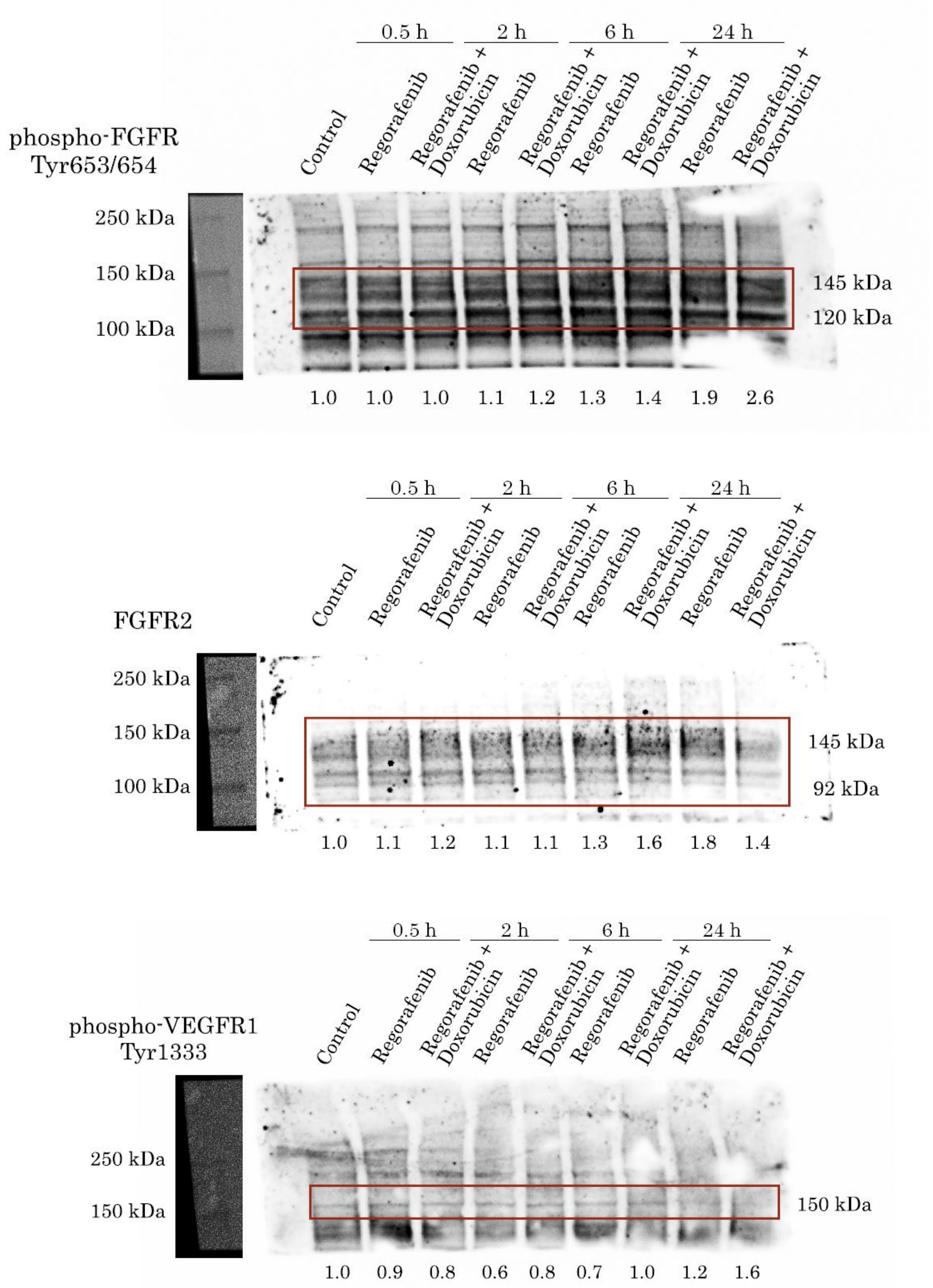

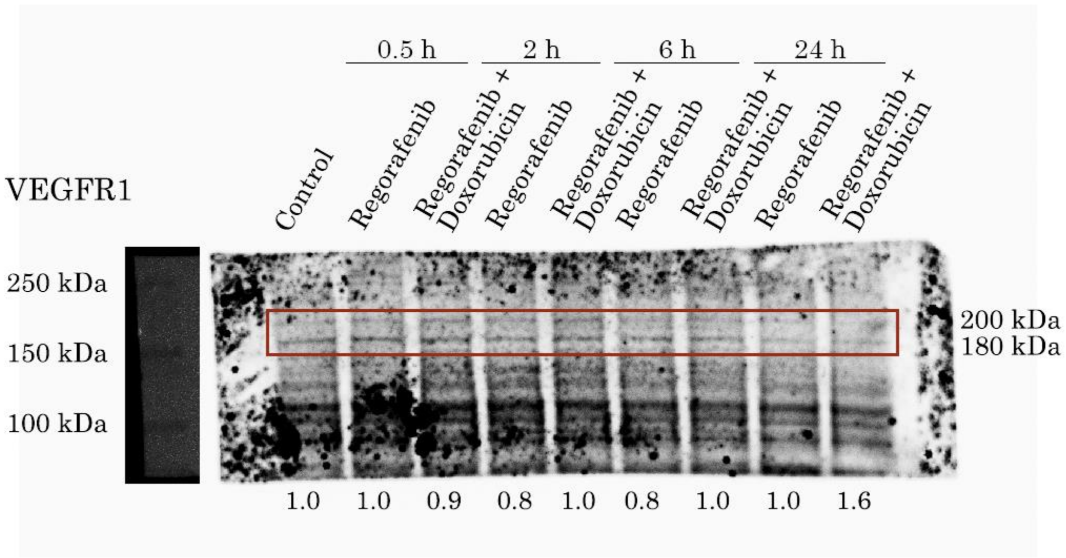
VEGFR1
0.5 h
2 h
6 h
24 h
Control
Regorafenib
Regorafenib + Doxorubicin
Regorafenib
Regorafenib + Doxorubicin
Regorafenib
Regorafenib + Doxorubicin
Regorafenib
Regorafenib + Doxorubicin
250 kDa
150 kDa
100 kDa
200 kDa
180 kDa
1.0 1.0 0.9 0.8 1.0 0.8 1.0 1.0 1.6

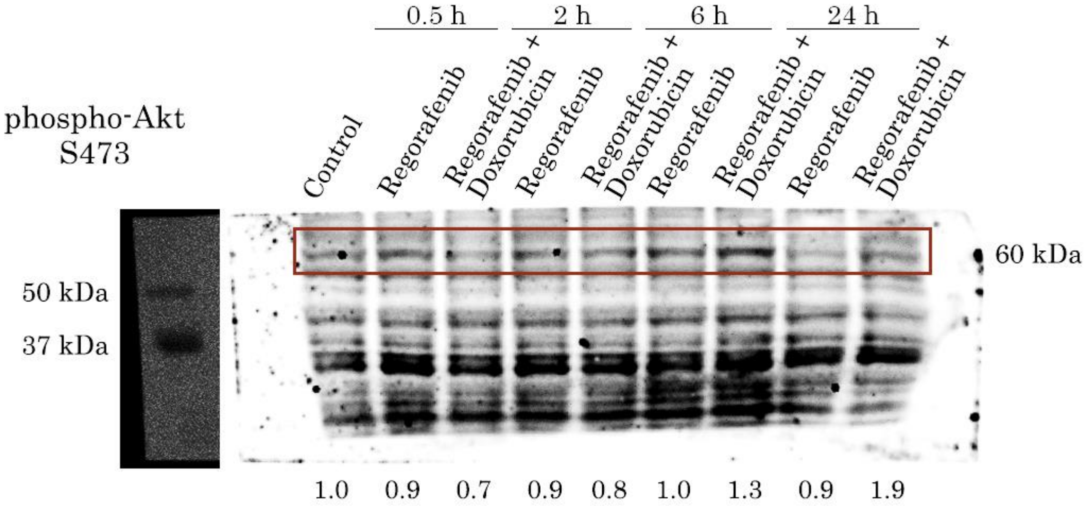
phospho-Akt S473
0.5 h
2 h
6 h
24 h
Control
Regorafenib
Regorafenib + Doxorubicin
Regorafenib
Regorafenib + Doxorubicin
Regorafenib
Regorafenib + Doxorubicin
Regorafenib
Regorafenib + Doxorubicin
50 kDa
37 kDa
60 kDa
1.0 0.9 0.7 0.9 0.8 1.0 1.3 0.9 1.9

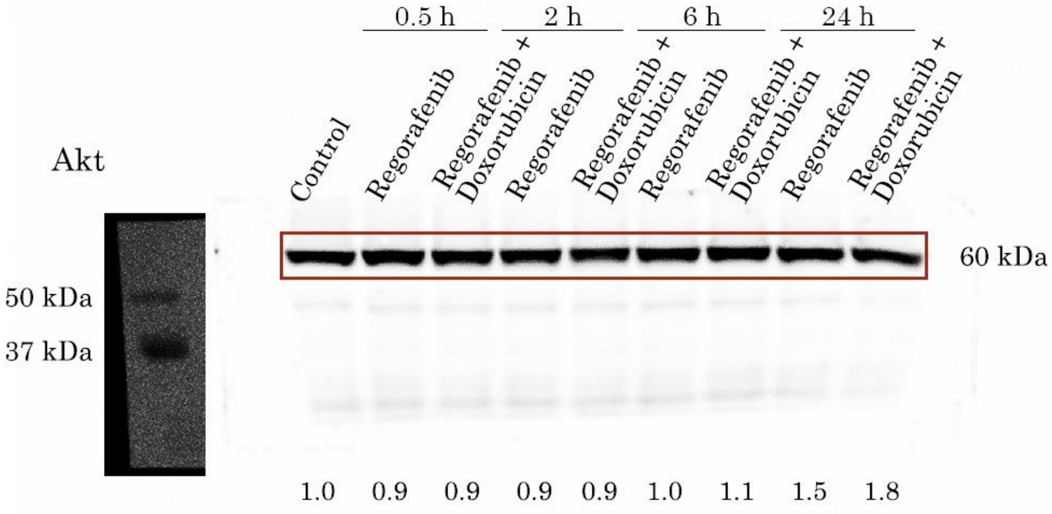
Akt
0.5 h
2 h
6 h
24 h
Control
Regorafenib
Regorafenib + Doxorubicin
Regorafenib
Regorafenib + Doxorubicin
Regorafenib
Regorafenib + Doxorubicin
Regorafenib
Regorafenib + Doxorubicin
50 kDa
37 kDa
60 kDa
1.0 0.9 0.9 0.9 0.9 1.0 1.1 1.5 1.8

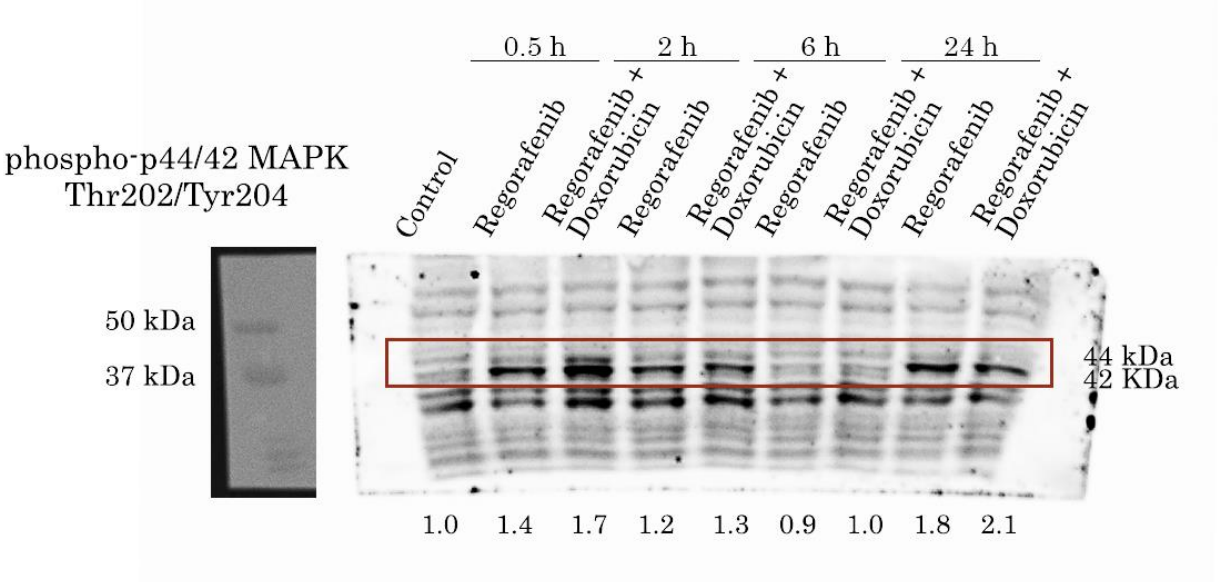
phospho-p44/42 MAPK
Thr202/Tyr204
0.5 h
2 h
6 h
24 h
Control
Regorafenib
Regorafenib + Doxorubicin
Regorafenib
Regorafenib + Doxorubicin
Regorafenib
Regorafenib + Doxorubicin
Regorafenib
Regorafenib + Doxorubicin
50 kDa
37 kDa
44 kDa
42 KDa
1.0 1.4 1.7 1.2 1.3 0.9 1.0 1.8 2.1

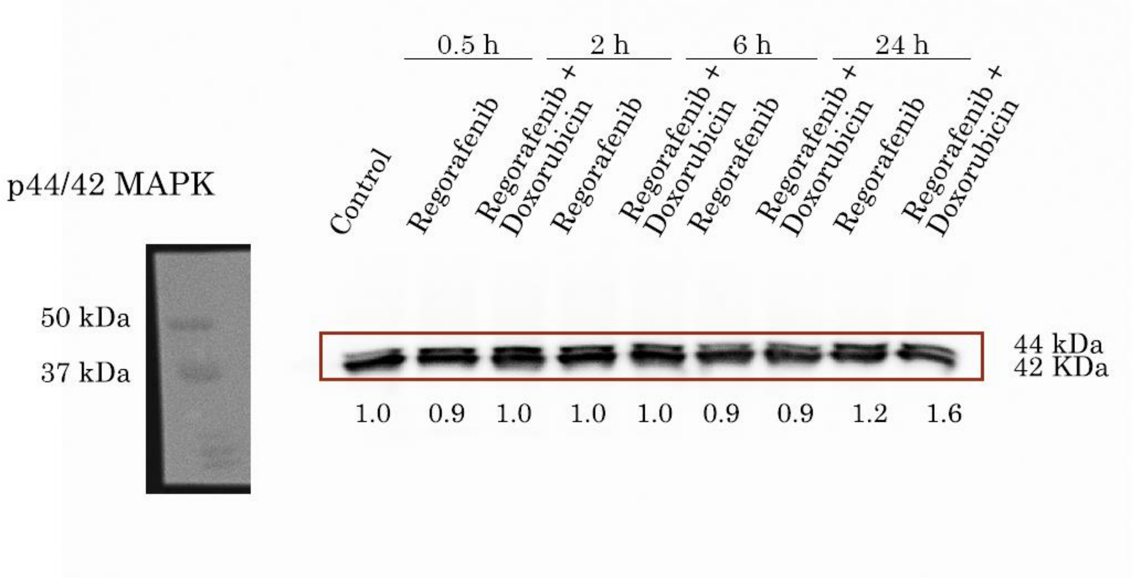
p44/42 MAPK
0.5 h
2 h
6 h
24 h
Control
Regorafenib
Regorafenib + Doxorubicin
Regorafenib
Regorafenib + Doxorubicin
Regorafenib
Regorafenib + Doxorubicin
Regorafenib
Regorafenib + Doxorubicin
50 kDa
37 kDa
44 kDa
42 KDa
1.0 0.9 1.0 1.0 1.0 0.9 0.9 1.2 1.6

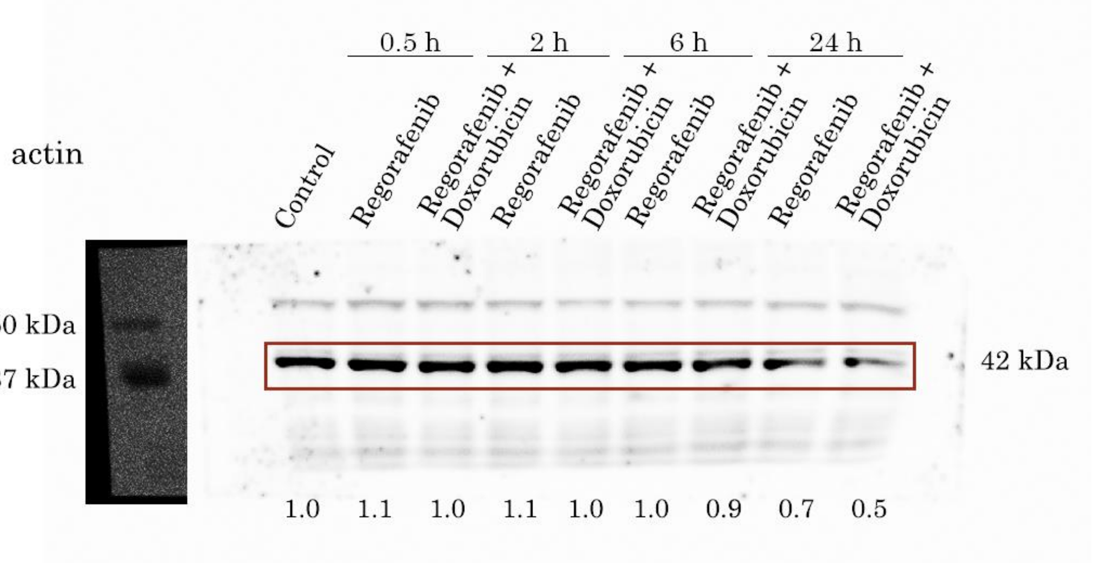
actin
0.5 h
2 h
6 h
24 h
Control
Regorafenib
Regorafenib + Doxorubicin
Regorafenib
Regorafenib + Doxorubicin
Regorafenib
Regorafenib + Doxorubicin
Regorafenib
Regorafenib + Doxorubicin
50 kDa
37 kDa
42 kDa
1.0 1.1 1.0 1.1 1.0 1.0 0.9 0.7 0.5

Figure S9. Lack of pro-apoptotic activity in HCT116 cells treated with Doxorubicin or Mitoxantron (40 µM) alone or in presence of Lucitanib and Regorafenib (20 µM) for 6 h.

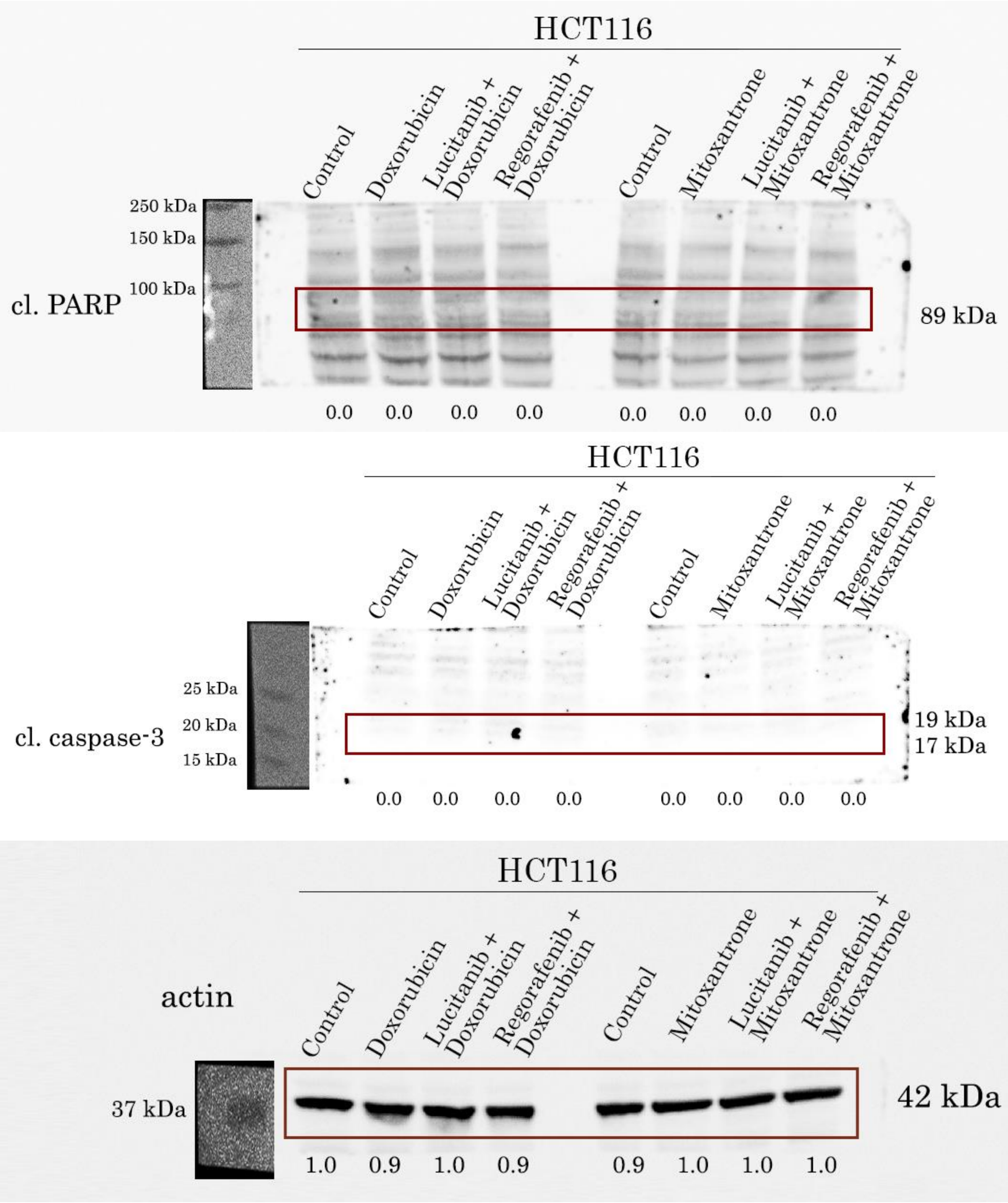

Supplement: Supplementary file 1 [file cancers-18-03074-s001.zip › File S1.pdf]
