# Supplementary material for: Re-Evaluating Multi-Kinase Inhibitors Targeting FGFR and VEGFR Pathways for Combined Therapy in Colorectal Cancer: A Preclinical Study
Source: Cancers (Basel). 2026 Sep 22;18(19):3074. doi: 10.3390/cancers18193074 (PMC13664539; doi:10.3390/cancers18193074)
Supplement: Supplementary file 1 [file cancers-18-03074-s001.zip › supplementary.pdf]

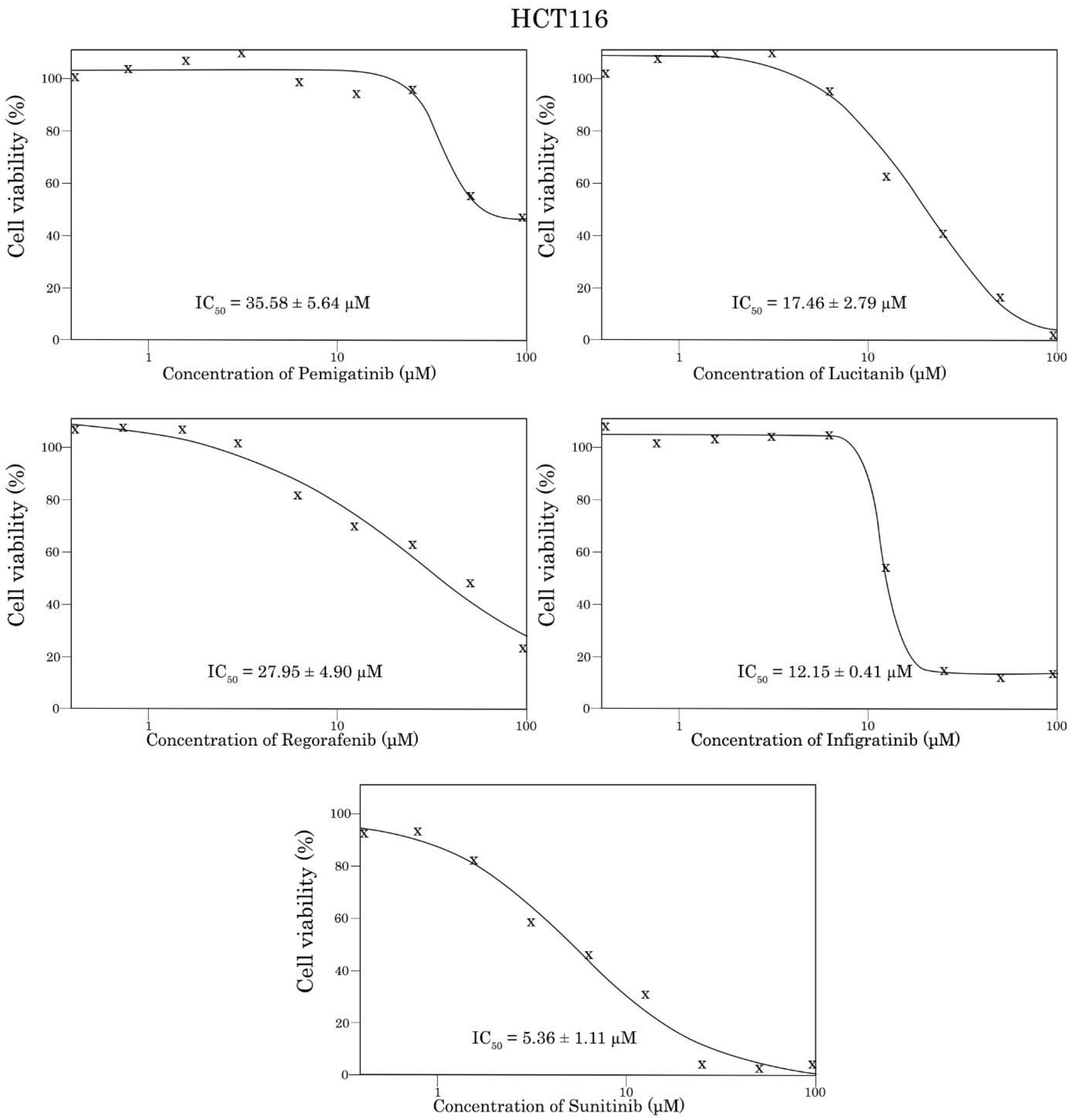

Figure S1. MTS-based viability assay in HCT116 cells treated with RTKIs for 72 h. The data were normalized to DMSO-treated controls.

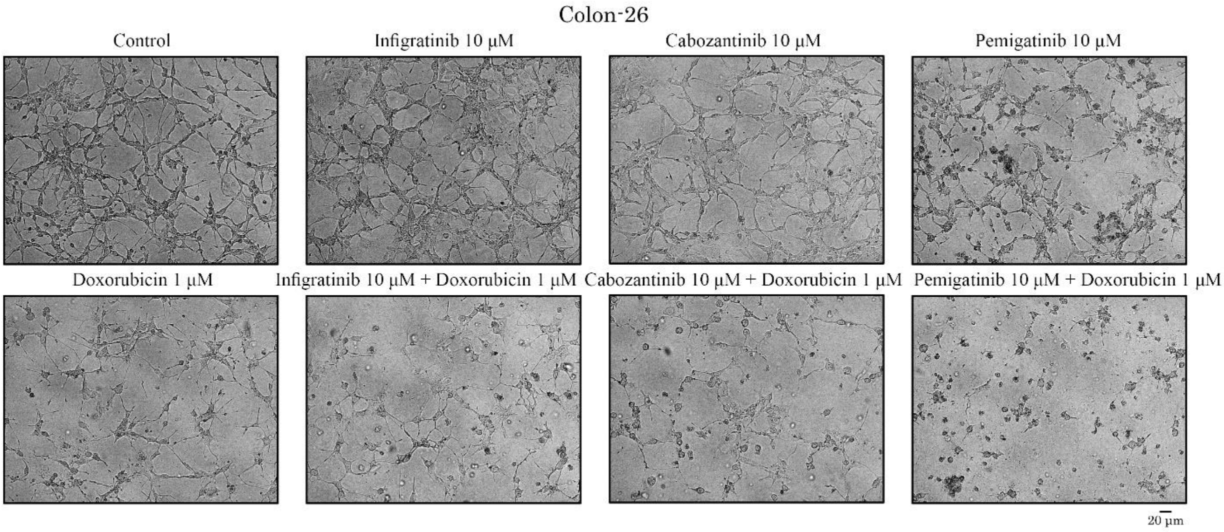

Figure S2. Representative brightfield images of Colon-26 colon cancer cells after exposure to DMSO (control), Doxorubicin (1 μM), RTKIs (Infigratinib, Cabozantinib, and Pemigatinib - 10 μM), and their combinations for 72 h. Combinational treatment significantly reduces cell confluency when compared with monotherapies and control.

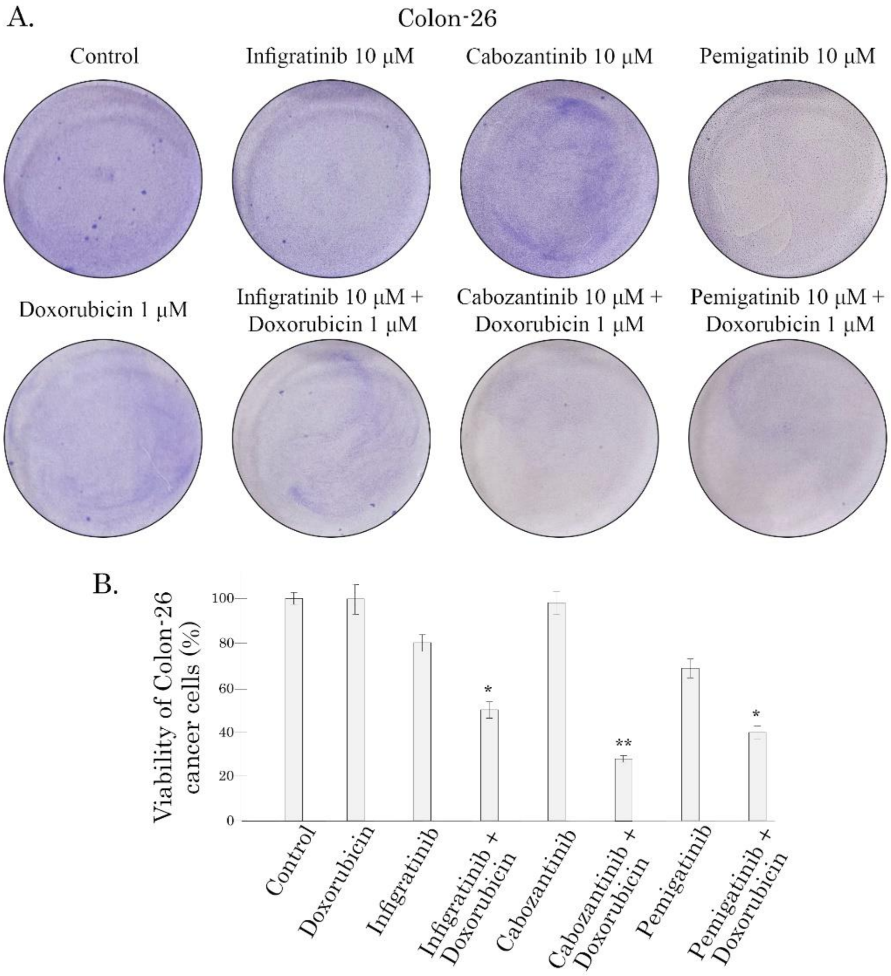

Figure S3. Cristal violet staining of Colon-26 cancer cells treated with DMSO (control), Doxorubicin (1 μM), and RTKIs (i.e., Infigratinib, Cabozantinib, Pemigatinib - 10 μM) alone for 72 h, and when combined with Doxorubicin. (**A**) Representative images of Colon-26 cancer cells stained with crystal violet solution after 72 hours of incubation with aforementioned chemotherapeutic drugs. (**B**) Quantitative analysis of Colon-26 cells stained with crystal violet solution. Control was taken as 100% of cellular viability. Data was presented as mean (%) ± SD (n=3). Significance level * $p<0.05$, ** $p<0.01$.

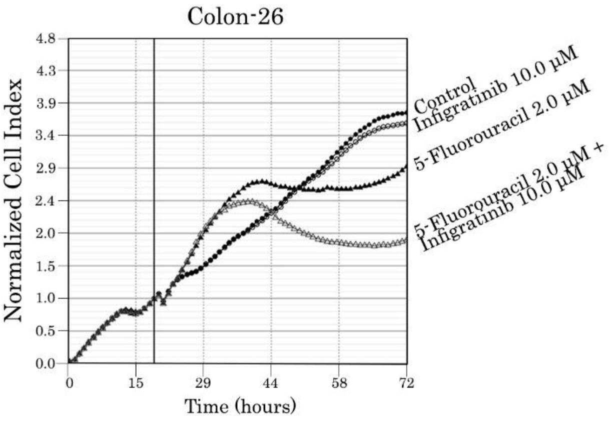

Figure S4. Anti-proliferative effects of Infigratinib used alone or in combination with 5-Fluorouracil in the Colon-26 colon cancer cell line. Changes in growth kinetics of cancer cells treated with solvent DMSO (negative control), 5-Fluorouracil, Infigratinib alone or in combination. Cells (0.5×10^5/ml) were seeded into the wells of an E-Plate L8 PET cassette and installed in the iCELLigence cell growth kinetics system (ACEA Biosciences, San Diego, CA, USA). Cells were allowed to attach and grow for the following 24 h. Afterward, Infigratinib, 5-Fluorouracil, or DMSO was introduced into the cell culture. Cell proliferation index values were recorded every hour throughout the experiment. RTCA Software version 1.0 (ACEA Biosciences, Inc., San Diego, CA, USA) was used to analyze the data.

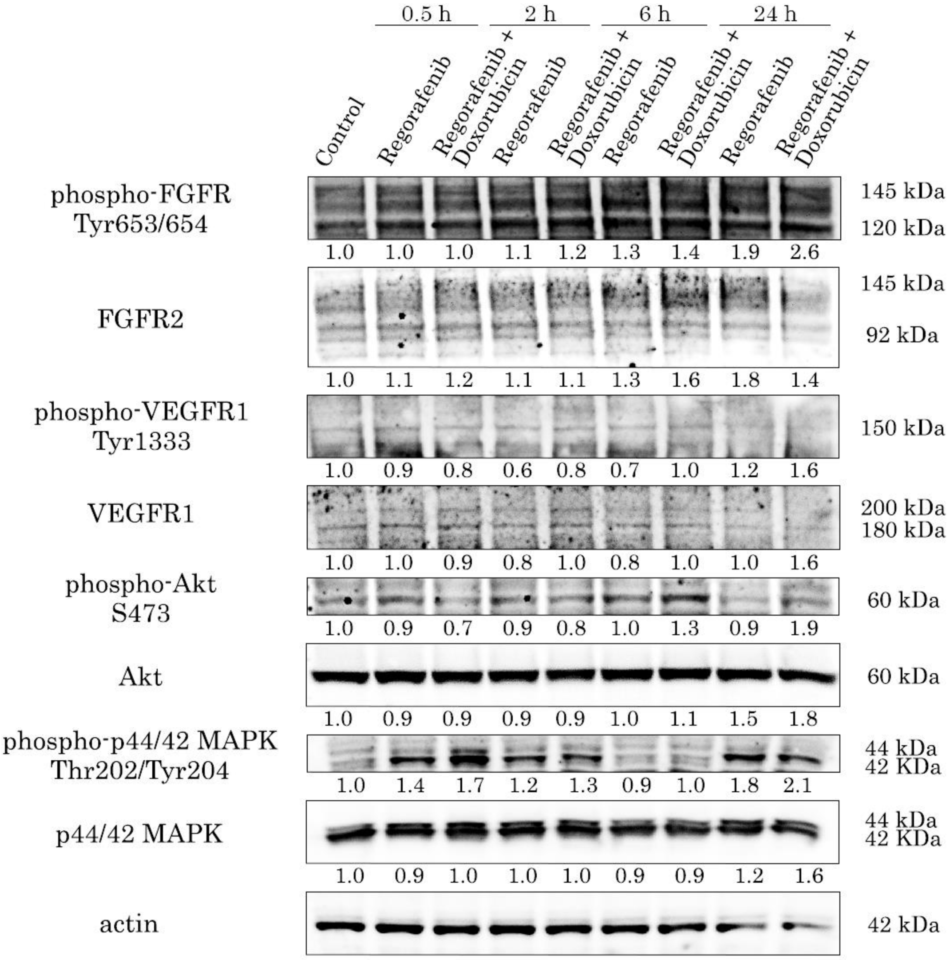

Figure S5. An impact of Regorafenib alone or in combination with Doxorubicin on FGFR and VEGFR signaling pathways in the HCT116 cancer cell line. Changes in expression of total and phosphorylated forms of FGFR1/2 and VEGFR1 and downstream signaling molecules AKT and MAPK were assessed at 0.5, 2, 6 and 24h of post treatment. Actin stain was used as a loading control.

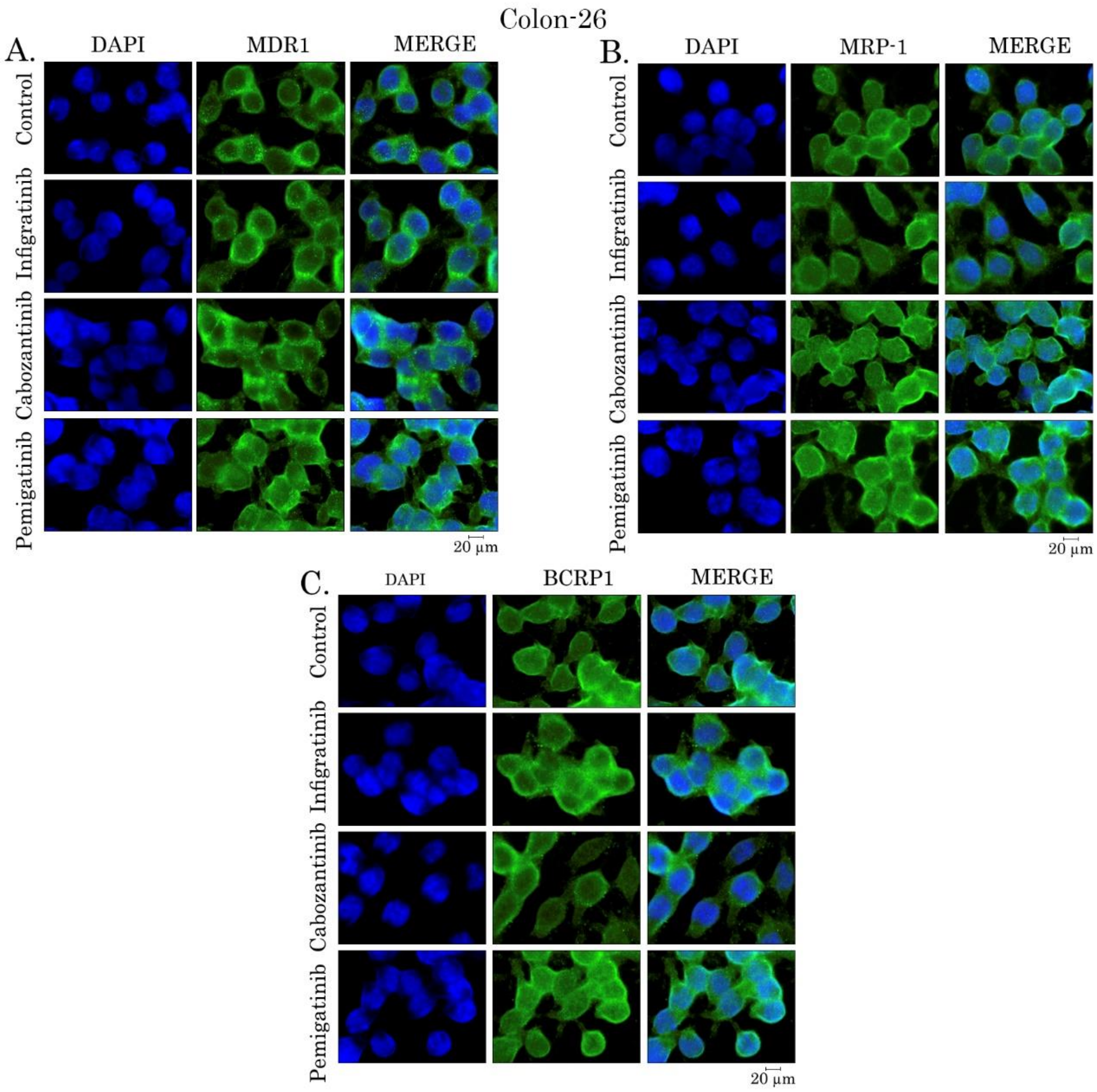

Figure S6. RTKI did not influence the subcellular localization of MDR1 (**A**), MRP-1 (**B**), and BCRP1 (**C**) in Colon-26 cells. Representative images of immunofluorescence staining of Colon-26 cancer cells incubated with DMSO (control) or Infigratinib, Cabozantinib, and Pemigatinib at 10 µM for 24 h are shown. Cell nuclei were outlined by staining with DAPI. Magnification 100x, scale bars 20 µm.

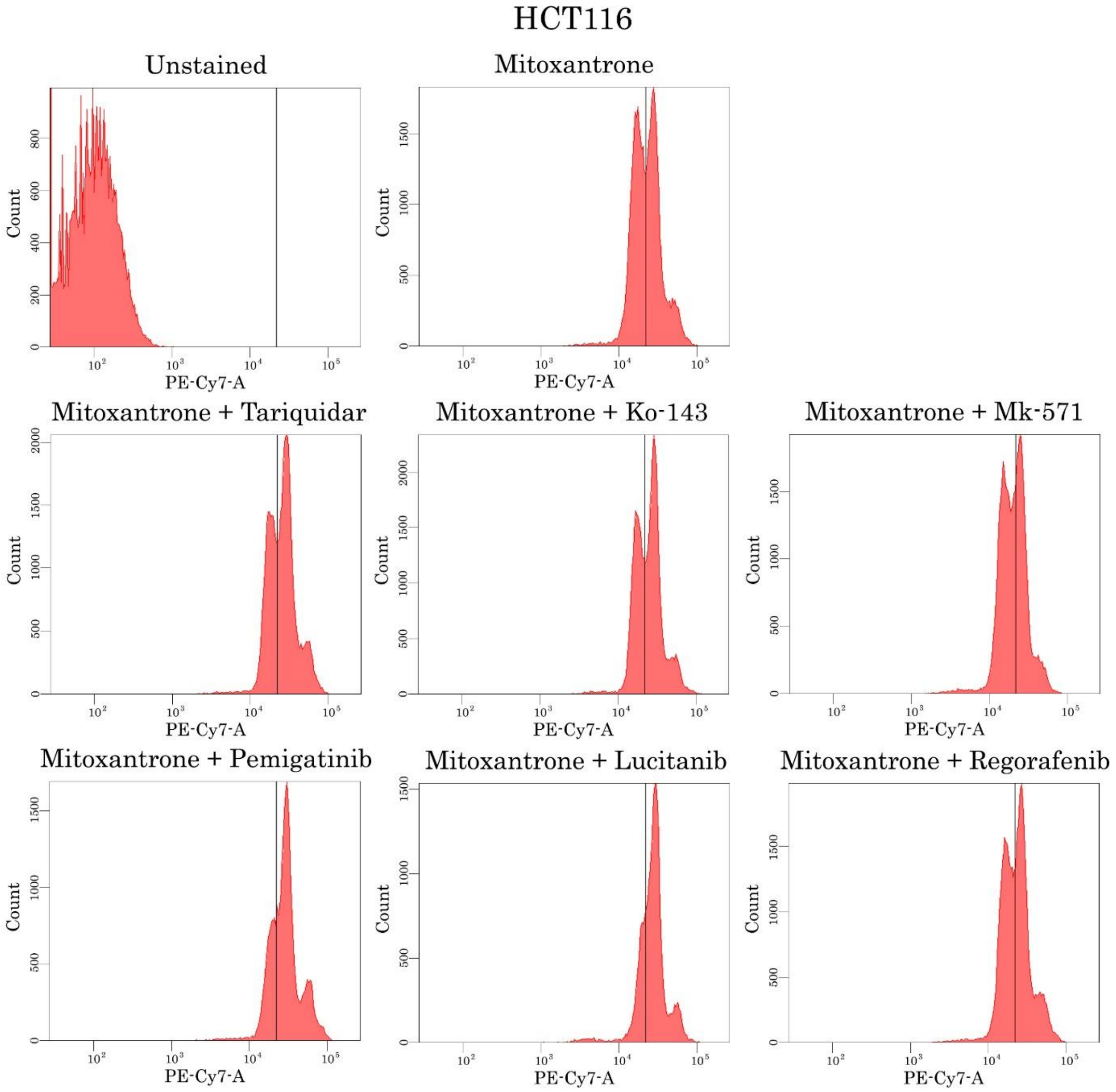

Figure S7. RTKIs increase the intracellular accumulation of Mitoxantrone in the HCT116 cancer cell line. The intracellular accumulation of Mitoxantrone in combination with ABC transporters inhibitors (Tariquidar, Ko-143, and Mk-571) and RTKIs (Pemigatinib, Lucitanib, and Regorafenib). The cells were treated with 40 µM of Mitoxantrone alone or with pre- and post-incubation with 20 µM of the aforementioned inhibitors. Representative histograms of at least 3 independent experiments are shown.

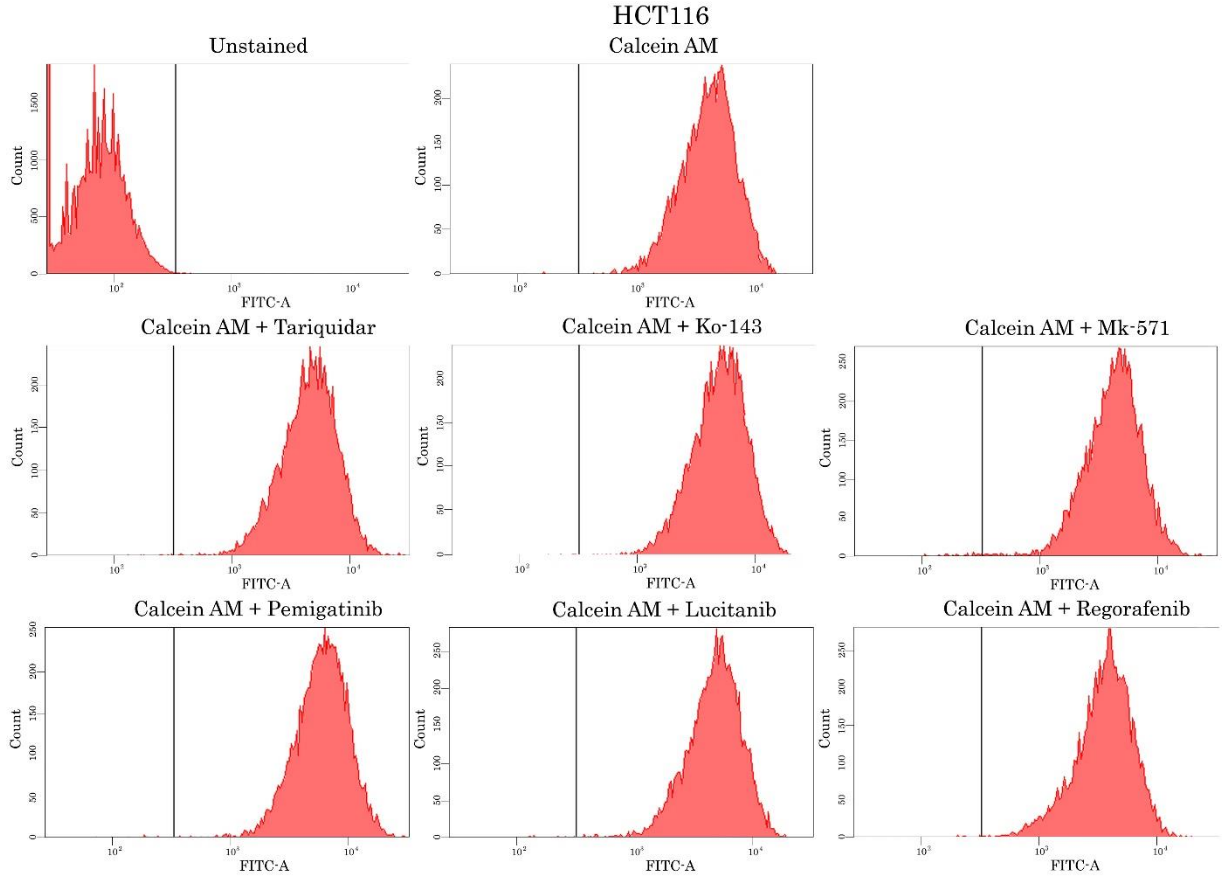

Figure S8. RTKIs increase the intracellular accumulation of Calcein AM, a specific MDR1 substrate, in the HCT116 cancer cell line. The intracellular accumulation of Calcein AM in combination with ABC transporters inhibitors (Tariquidar, Ko-143, and Mk-571) and RTKI (Pemigatinib, Lucitanib, Regorafenib). First, all cells were cultured with 100 nM Calcein AM alone, and then in culture medium with or without 20 μM of the aforementioned inhibitors. Representative histograms of at least 3 independent experiments are shown.

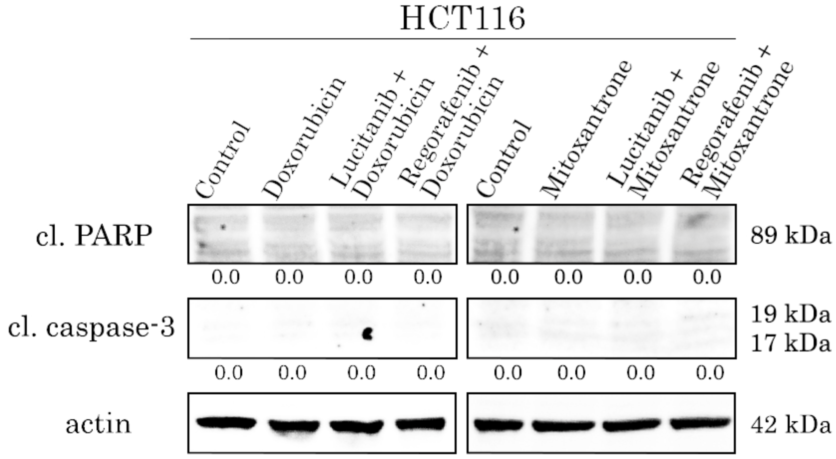

Figure S9. Lack of pro-apoptotic activity in HCT116 cells treated with Doxorubicin or Mitoxantron (40 µM) alone or in presence of Lucitanib and Regorafenib (20 µM) for 6 h. Cleaved poly-(ADP)-ribose-polymerase (PARP) and caspase-3 stain were used as apoptotic markers, whereas actin staining was used to show the comparable amounts of protein loaded into each sample.

Table S1. Molecular targets and clinical application of the RTKI.

	Molecular target	Clinical application	References ##
**Selective inhibitors of the FGFR signaling pathway**			
Infigratinib (BGJ398)	FGFR1, FGFR2 and FGFR3	Cholangiocarcinoma	[64,65]
Pemigatinib (INCB054828)	FGFR1, FGFR2, FGFR3 and FGFR4	Cholangiocarcinoma, Myeloid/Lymphoid Neoplasms	[66-68]
Alofanib (RPT835)	FGFR2	Gastric Cancer	[69]
**Non-selective RTKI targeting FGFR and/or VEGFR signaling pathways**			
Lucitanib (E3810)	VEGFR1, VEGFR2, VEGFR3, FGFR1 and FGFR2	Breast Cancer	[70-72]
Axitinib (AG-013736)	VEGFR1, VEGFR2, VEGFR3, PDGFRβ and c-Kit	Renal Cell Carcinoma	[73,74]
Cabozantinib (XL184)	VEGFR2, c-Met, Ret, Kit, Flt-1/3/4, Tie2, and AXL	Advanced Renal Cell Carcinoma, Hepatocellular Carcinoma, Thyroid Cancer, Pancreatic and Extrapancreatic Neuroendocrine Tumors	[75-78]
Regorafenib (BAY 73-4506)	VEGFR1, VEGFR2, VEGFR3, PDGFRβ, Kit (c-Kit), RET (c-RET) and Raf-1	Metastatic Colorectal Cancer, Imatinib- and Sunitinib-refractatory Gastrointestinal Stromal Tumors, Hepatocellular Carcinoma	[79-82]
Sunitinib (SU-11248)	VEGFR2, PDGFRβ and c-Kit	Pancreatic Neuroendocrine Tumors, Metastatic Renal Cell Carcinoma, Gastrointestinal Stromal Tumors	[83-85]

Table S2. Synergy scores (ZIP model) between the most potent RTKI and Doxorubicin or 5-Fluorouracil in colorectal cancer cell lines.

Colon-26		HCT116		RKO	
Doxorubicin + Infigratinib	15.09	Doxorubicin + Pemigatinib	-2.81	Doxorubicin + Cabozantinib	0.83
Doxorubicin + Cabozantinib	16.88	Doxorubicin + Lucitanib	2.15	Doxorubicin + Infigratinib	-3.98
Doxorubicin + Pemigatinib	11.54	Doxorubicin + Regorafenib	2.54	Doxorubicin + Alofanib	-2.75
5-Fluorouracil + Pemigatinib	-2.99	5-Fluorouracil + Sunitinib	-5.47	5-Fluorouracil + Lucitanib	-1.15
5-Fluorouracil + Infigratinib	N.D.	5-Fluorouracil + Pemigatinib	4.00	5-Fluorouracil + Cabozantinib	-1.30
5-Fluorouracil + Lucitanib	-0.27	5-Fluorouracil + Regorafenib	-7.39	5-Fluorouracil + Pemigatinib	-11.55

Table S3. The expression of *MDR1, MRP-1,* and *ABCG2 mRNA* in CRC cells when compared with chemo-resistant HCC1806 TxR triple-negative breast cancer cell sub-line. Values are averages of 3 experiments. Significance level * p<0.01, ** p<0.001, *** p<0.0001.

Genes	*MDR1*	*MRP1*	*ABCG2*
**HCT116**	0,3	5,730***	1,2
**RKO**	0,88	19,320***	0,262
**Colon-26**	0,84	100,130***	-

Table S4. The expression of *MDR1, MRP-1,* and *ABCG2 mRNA* in CRC cells when compared with chemoresistant SaOS-2 DoxR osteosarcoma cancer cell sub-line. Values are averages of 3 experiments. Significance level * p<0.01, ** p<0.001, *** p<0.0001.

Genes	*MDR1*	*MRP1*	*ABCG2*
**HCT116**	6,46**	16,36**	16,43***
**RKO**	19,05***	55,09***	3,58*
**Colon-26**	18,04***	285,56***	-

Table S5. The Mean Fluorescence Intensity of Mitoxantrone in HCT116 cells was determined from 3 independent experiments. The intracellular accumulation of Mitoxantrone (40 μM) alone and with pre- and post-incubation with ABC transporter inhibitors (Tariquidar, Ko-143, and Mk-571) and RTKIs (Pemigatinib, Lucitanib, and Regorafenib) at a concentration of 20 μM. Significance level * $p<0.05$, ** $p<0.01$.

**HCT116**	
Doxorubicin	2407 ± 163
Doxorubicin + Tariquidar	4129 ± 345 **
Doxorubicin + Ko-143	3068 ± 210 *
Doxorubicin + Mk-571	2329 ± 116
Doxorubicin + Pemigatinib	3187 ± 370 *
Doxorubicin + Lucitanib	3677 ± 494 **
Doxorubicin + Regorafenib	2188 ± 204

Table S6. The Mean Fluorescence Intensity of Calcein AM, a specific MDR1 substrate, in HCT116 cells was determined from 3 independent experiments. The intracellular accumulation of Calcein AM alone and in combination with ABC transporter inhibitors (Tariquidar, Ko-143, and Mk-571) and RTKI (Pemigatinib, Lucitanib, Regorafenib) in HCT116. Significance level * p<0.05, ** p<0.01.

**HCT116**	
Calcein AM	4601 ± 385
Calcein AM + Tariquidar	5849 ± 421 *
Calcein AM + Ko-143	5639 ± 486
Calcein AM + Mk-571	5234 ± 537
Calcein AM + Pemigatinib	7040 ± 574 **
Calcein AM + Lucitanib	5479 ± 497
Calcein AM + Regorafenib	4213 ± 402

Table S7. FACs analysis of cell cycle profile of Colon-26 cells treated with RTKIs (10 μM) or Doxorubicin (1 μM) for 72h.

	**Sub-G0/G1**	**G0/G1**	**S**	**G2/M**
Control	2.12 ± 0.14	62.65 ± 2.56	12.02 ± 0.94	21.97 ± 1.78
Doxorubicin	5.22 ± 0.32	63.89 ± 4.27	9.48 ± 0.86	19.20 ± 1.54
Infigratinib	1.97 ± 0.10	61.90 ± 3.65	11.95 ± 0.83	21.53 ± 2.05
Cabozantinib	4.11 ± 0.28	63.40 ± 5.38	9.88 ± 0.92	20.99 ± 0.97
